# Temporality of body mass index, blood tests, comorbidities and medication use as early markers for pancreatic ductal adenocarcinoma (PDAC): a nested case–control study

**DOI:** 10.1136/gutjnl-2021-326522

**Published:** 2022-06-27

**Authors:** Pui San Tan, Cesar Garriga, Ashley Clift, Weiqi Liao, Martina Patone, Carol Coupland, Rachael Bashford-Rogers, Shivan Sivakumar, Julia Hippisley-Cox

**Affiliations:** 1 Nuffield Department of Primary Care Health Sciences, University of Oxford, Oxford, UK; 2 Department of Oncology, Cancer Research UK Oxford Centre, University of Oxford, Oxford, UK; 3 Division of Primary Care, School of Medicine, University of Nottingham, Nottingham, UK; 4 Wellcome Centre for Human Genomics, University of Oxford, Oxford, UK; 5 Department of Oncology, University of Oxford, Oxford, UK; 6 Kennedy Institute of Rheumatology, University of Oxford, Oxford, UK; 7 Department of Oncology, Oxford University Hospitals NHS Foundation Trusts, Oxford, UK

**Keywords:** cancer epidemiology, pancreatic cancer

## Abstract

**Objective:**

Prior studies identified clinical factors associated with increased risk of pancreatic ductal adenocarcinoma (PDAC). However, little is known regarding their time-varying nature, which could inform earlier diagnosis. This study assessed temporality of body mass index (BMI), blood-based markers, comorbidities and medication use with PDAC risk.

**Design:**

We performed a population-based nested case–control study of 28 137 PDAC cases and 261 219 matched-controls in England. We described the associations of biomarkers with risk of PDAC using fractional polynomials and 5-year time trends using joinpoint regression. Associations with comorbidities and medication use were evaluated using conditional logistic regression.

**Results:**

Risk of PDAC increased with raised HbA1c, liver markers, white blood cell and platelets, while following a U-shaped relationship for BMI and haemoglobin. Five-year trends showed biphasic BMI decrease and HbA1c increase prior to PDAC; early-gradual changes 2–3 years prior, followed by late-rapid changes 1–2 years prior. Liver markers and blood counts (white blood cell, platelets) showed monophasic rapid-increase approximately 1 year prior. Recent diagnosis of pancreatic cyst, pancreatitis, type 2 diabetes and initiation of certain glucose-lowering and acid-regulating therapies were associated with highest risk of PDAC.

**Conclusion:**

Risk of PDAC increased with raised HbA1c, liver markers, white blood cell and platelets, while followed a U-shaped relationship for BMI and haemoglobin. BMI and HbA1c derange biphasically approximately 3 years prior while liver markers and blood counts (white blood cell, platelets) derange monophasically approximately 1 year prior to PDAC. Profiling these in combination with their temporality could inform earlier PDAC diagnosis.

**WHAT IS ALREADY KNOWN ON THIS TOPIC**

- Prior studies identified clinical factors and markers which are associated with increased risk of pancreatic ductal adenocarcinoma (PDAC) diagnosis. However, little is known regarding the time-varying nature of these risk associations, which may aid earlier diagnosis.

**WHAT THIS STUDY ADDS**

- In this large nested case–control study, we identified risk associations and 5-year trends in body mass index (BMI) and widely tested blood-based markers that differed between those that developed PDAC and those that did not. Risk of PDAC increased with raised HbA1c, liver markers, white blood cell and platelets, while following a U-shaped relationship for BMI and haemoglobin. BMI and HbA1c derange biphasically approximately 3 years prior while liver markers and blood counts (white blood cell, platelets) derange monophasically approximately 1 year prior to PDAC.

**HOW THIS STUDY MIGHT AFFECT RESEARCH, PRACTICE AND/OR POLICY**

- Temporal information on BMI, blood markers, comorbidities and medication initiation could be integrated into risk prediction tools to identify patients at increased risk of PDAC. This may help form an enriched population who could benefit from further investigations or screening to aid earlier diagnosis of PDAC.

## Introduction

Pancreatic ductal adenocarcinoma (PDAC) is the most common form of pancreatic malignancy and is associated with almost universally poor prognosis mainly attributed to late-stage diagnosis.^1 2^ Currently, there is no screening programme, and symptom-based early detection remains challenging due to the vague or asymptomatic nature of earlier stage disease.^3–5^

While understanding the symptom profiles of pancreatic cancer presents one opportunity for earlier diagnosis,^6 7^ there remains a need to robustly identify clinical and biochemical factor associations to inform the identification of higher risk subpopulations for earlier intervention with imaging or recruitment to screening studies.

An earlier small study suggested that fasting blood glucose was significantly elevated beginning 3 years prior to pancreatic cancer diagnosis and BMI significantly decreased beginning 1 year prior.^8^ However, besides being limited by its relatively small sample size, the majority of patients in this study had only one observation up to 12 months prior to diagnosis, and hence, would not serve as a robust assessment for longer time trends. Further, evidence on other commonly used blood tests that is, liver markers and blood counts and their temporal associations relating to pancreatic cancer remains limited. In addition, some research has identified chronic pancreatitis, prior cancers, type 2 diabetes (T2D) mellitus and antidiabetic medications to be associated with increased risk of PDAC diagnosis, but limited studies on their temporal trends are available.^9–14^

Nonetheless, although the association with T2D is of uncertain directionality or causality,^10–14^ the National Institute for Health and Care Excellence guidelines in England recommend an urgent, 2-week wait CT scan for those aged 60 years and over with new-onset diabetes and weight loss.^15^ However, there are approximately 200 000 new diagnoses of T2D per year in England,^16^ and it remains challenging to universally perform CT scans on this population for pancreatic cancer. Hence, understanding the magnitude and temporality of additional risk factors could help to identify enriched populations at high risk of pancreatic cancer who could have greater benefit from screening.

Here, we use a matched case–control study nested within a population-level electronic healthcare database in England (UK) to assess temporal associations between BMI, blood-based markers, comorbidities and medication use with risk of PDAC diagnosis. These could be used to better identify those that may benefit from expedited or targeted investigations and enhanced surveillance.

## Methods

### Study design and population

A nested case–control study was carried out using the QResearch primary care database (V. 45) with individual-level linkages to the national cancer registry (Public Health England), Hospital Episode Statistics (HES) and Office for National Statistics death registry in England. Adult patients aged 18 years and above were eligible for inclusion and entered the study at the latest of: date of registration with practice plus 1 year, date general practice started contributing data to QResearch plus 1 year.

### Selection of cases and controls

Cases were defined as patients diagnosed with PDAC as recorded in any of the primary care, cancer registry, hospital records (HES) or death registry from January 2000 to October 2020. PDAC was identified using READ/SNOMED codes for general practitioner (GP) records and ICD-10 codes for cancer, hospital and death registries records. ICD-10 codes and ICD-0 codes (cancer registry) used to identify PDAC cases are provided in online supplemental list S1. Histological codes as recorded in the cancer registry were reviewed to avoid the inclusion of non-PDAC tumours, such as neuroendocrine neoplasms. If two or more cancer records were present, only the first primary pancreatic cancer was included in the analysis. Incidence density sampling was used to match each case to up to 10 controls (with no PDAC diagnosis) by age, sex, practice and calendar year of diagnosis. The index date was defined as PDAC diagnosis date for cases, and the corresponding matched date for controls.

10.1136/gutjnl-2021-326522.supp1 Supplementary data

### Definitions of exposures

We investigated time trends of the following markers^7 17 18^: body mass index (BMI) and blood-based markers (8) (HbA1c (haemoglobin A1c), ALT (alanine transaminase), AST (aspartate aminotransferase), GGT (gamma-glutamyl transferase), bilirubin, haemoglobin (Hb), white blood cell (WBC), platelets) at 3-monthly intervals for up to 5 years prior to the index date by T2D status (early-onset, long-standing, none). Recent onset was defined as first recorded T2D within 3 years before index date and long-standing was defined as first recorded T2D more than 3 years prior to index date.

Further, we identified 18 medical conditions^7 8 19–22^ and 16 medications^23–32^ from prior literature and evaluated their association with risk of PDAC diagnosis—detailed list is provided in online supplemental list S2.

Exposure variables were further categorised as: recent onset/initiation (first recorded within 3 years before index date), long-standing (more than 3 years prior to index date), versus none.

Presence of medical conditions was based on first recorded diagnosis while medication use was based on first recorded prescription in primary care records. Blood test values were those recorded in the primary care database, which covers tests undertaken in primary care, or results requested in primary care but undertaken elsewhere and reported back. BMI was obtained using recorded values of height and weight from GP records. They were measured during consultations and keyed into the primary care database by clinical staff.

### Confounders

We assessed potential confounders based on directed acyclic graphs (DAGs) (online supplemental figure S1) and included demographics and lifestyle variables as confounder adjustments in the comorbidities regression model: demographic (ethnic group, Townsend Deprivation Score)^33^ and lifestyle (BMI, smoking and alcohol consumption). In the medications regression model, demographics, lifestyle factors and comorbidities were included for confounder adjustment. Most recent measurements for BMI, smoking status and alcohol use prior to index date were used.

### Statistical analysis

#### Fractional polynomials and temporal trends analysis

First, we evaluated the associations of BMI and biomarker levels with risk of PDAC. We evaluated non-linear trends using fractional polynomial with up to two terms for measurements taken within 3 years and 1 year prior to the index date. If more than one measurement was available during the period, the reading closest to the cut-off time that is, 3 and 1 year was taken.

Then, we evaluated the temporal trends of BMI and blood-based markers (HbA1c, ALT, AST, GGT, bilirubin, Hb, WBC, platelets) up to 5 years prior to the index date by T2D status (recent onset, long-standing, no T2D). Joinpoint regression assuming constant variance and uncorrelated errors was used to evaluate 5-year time trends of mean BMI and blood marker values in 3-monthly time intervals prior to index month.^34–36^ Joinpoint regression or change-point regression is commonly used to assess changes in time trends.^34–36^ It evaluates the number of joinpoints needed, that is, when linear trends start to have different intercept and slope—beginning with the simplest model (0 joinpoints, ie, straight line), and tests if addition of joinpoints (change in trend) is significant and needed using Monte-Carlo Permutation significance test.^34–36^ Joinpoints were placed in locations where trends of biomarker values become significantly different to produce the best fit line, with corresponding slopes after each joinpoint illustrated in each figure legend.

Multiple imputation was performed using chained equations under the missing at random assumption to impute missing values of observations. Fifty imputations were performed using a comprehensive model including outcome, demographics, comorbidities and medications. The number of complete observations included for each biomarker in the 5-year time trend analysis is detailed in online supplemental table S1.

#### Temporal associations analysis

Conditional logistic regression was used to estimate the association of PDAC risk and each temporal category of the comorbidities and medications use variables adjusted for confounders, with BMI modelled using restricted cubic spline terms with three knots. Associations with comorbidities and medications use were modelled separately; the comorbidities model included adjustments for demographics and lifestyle factors, while the medications use model included adjustments for demographics, lifestyle factors, and comorbidities, as guided by DAGs (online supplemental figure S1).

Associations were estimated in terms of adjusted ORs and corresponding 95% CI. Multiple imputation was performed using chained equations under the missing at random assumption to impute missing values of ethnicity (31.8%), deprivation (0.2%), BMI (17.6%), smoking (8.6%) and alcohol consumption (17.1%). Ten imputations were performed using a comprehensive model including outcome, exposures and confounder variables. Fitted model coefficients and standard errors were pooled using Rubin’s rules.^37^

#### Sensitivity analyses

We further performed three sensitivity analyses to: (1) evaluate longitudinal trends using joinpoint analysis in complete-case dataset, (2) evaluate longitudinal trends using fractional polynomials and corresponding 95% CI at 3-monthly time intervals using both multiply imputed and complete-case dataset, and (3) evaluate associations of comorbidities and medication use within 2 years prior to the index date with risk of PDAC. In contrast with joinpoint analysis, two-way fractional polynomials find the best fit curve using a polynomial functional form to model biomarker values over time.^38^

We ran additional post-hoc subgroup analyses for associations of comorbidities and medications with risk of PDAC by categories of age, sex, year of diagnosis, BMI, smoking status and T2D status. BMI, smoking status and T2D status were not matching variables in the case–control study design, hence, these subgroup analysis needed to be performed without matching and analysed using mixed effects logistic regression with practice as a random-effect variable and additionally adjusted for other matching factors (age, sex, year of diagnosis).

We computed E-values to measure the amount of potential unmeasured confounding for all statistically significant risk factors.^39 40^ Large E-values imply a relatively large unmeasured confounding effect is needed to explain away residual confounding, while small E-values imply a small unmeasured confounding effect is needed to explain away any residual confounding)^39 40^

Finally, while our work was descriptive in intention there may be the issue of multiple significance testing, so we evaluated associations that remained significant at the 5% level after a Bonferroni correction. All statistical analyses were performed using STATA V.17.0^41^ and time trend analyses were performed using JoinPoint software.^34^

### Patient and public involvement

We involved participation of lay members of the Pancreatic Cancer UK Research Involvement Network to review and comment on the lay summary of the protocol which is now published on the QResearch website.^42^

## Results

A total of 2 89 356 patients were included in the study, comprising 28 137 incident PDAC cases and 261 219 matched controls. Cases had a mean age of 72.8 years at diagnosis and 50.1% were female. T2D was approximately twice as common in cases than controls (24.6% vs 12.6%, respectively), and recent-onset T2D was approximately four times as common (9.7% vs 2.5%, respectively). Detailed characteristics of cases and controls are provided in table 1 with onset and initiation time of comorbidities as well as medication use provided in online supplemental table S2.

**Table 1:** Characteristics of study population with PDAC cases and controls matched by age, sex, practice and calendar year of diagnosis

Characteristics		PDAC cases	Controls
Total, N		28 137	261 219
Age at index date	Mean (SD)	72.8 (11.9)	72.0 (11.7)
Sex	Female	14 106 (50.1)	131 338 (50.3)
	Male	14 031 (49.9)	129 881 (49.7)
Ethnicity	White	14 531 (51.6)	167 751 (64.2)
	Indian	213 (0.8)	3038 (1.2)
	Pakistani	132 (0.5)	1376 (0.5)
	Bangladeshi	63 (0.2)	806 (0.3)
	Other Asian	104 (0.4)	1464 (0.6)
	Black Caribbean	287 (1.0)	2606 (1.0)
	Black African	142 (0.5)	1646 (0.6)
	Chinese	43 (0.2)	555 (0.2)
	Other	223 (0.8)	2257 (0.9)
	Not recorded	12 399 (44.1)	79 720 (30.5)
Deprivation quintile	1 (least deprived)	8281 (29.4)	81 857 (31.3)
	2	6922 (24.6)	66 688 (25.5)
	3	5688 (20.2)	50 582 (19.4)
	4	4217 (15.0)	36 442 (14.0)
	5 (most deprived)	2968 (10.5)	25 246 (9.7)
	Not recorded	61 (0.2)	404 (0.2)
Body mass index (kg/m^2^)	<18.5	807 (2.9)	3919 (1.5)
	18.5 to <25	8923 (31.7)	73 050 (28.0)
	25 to <30	8735 (31.0)	85 354 (32.7)
	30 to <35	3738 (13.3)	36 753 (14.1)
	35 to <40	1125 (4.0)	11 368 (4.4)
	≥40	385 (1.4)	4203 (1.6)
	Not recorded	4424 (15.7)	46 572 (17.8)
Smoking status	Non-smoker	12 557 (44.6)	129 951 (49.7)
	Ex-smoker	8238 (29.3)	76 820 (29.4)
	Light smoker (1–10 cigarettes/day)	2791 (9.9)	17 868 (6.8)
	Moderate smoker (11–19/day)	1371 (4.9)	8302 (3.2)
	Heavy smoker (≥20/day)	1037 (3.7)	5539 (2.1)
	Not recorded	2143 (7.6)	22 739 (8.7)
Alcohol consumption	Non-drinker	9416 (33.5)	77 786 (29.8)
	Trivial <1 unit/day	7126 (25.3)	69 850 (26.7)
	Light 1–2 units/day	3208 (11.4)	32 104 (12.3)
	Moderate 3–6 units/day	3607 (12.8)	32 665 (12.5)
	Heavy 7–9 units/day	343 (1.2)	2522 (1.0)
	Very heavy >9 units/day	171 (0.6)	1068 (0.4)
	Not recorded	4266 (15.2)	45 224 (17.3)
Comorbidities	Type 2 diabetes	6925 (24.6)	32 962 (12.6)
	Pre-diabetes	1672 (5.9)	11 167 (4.3)
	Acute pancreatitis	737 (2.6)	1568 (0.6)
	Chronic pancreatitis	321 (1.1)	356 (0.1)
	Hypercholesterolaemia	4911 (17.5)	45 179 (17.3)
	Venous thromboembolism	1646 (5.8)	9058 (3.5)
	Asthma	2990 (10.6)	26 659 (10.2)
	Inflammatory bowel disease	337 (1.2)	2709 (1.0)
	Coeliac disease	143 (0.5)	857 (0.3)
	Breast cancer*	771 (2.7)	5957 (2.3)
	Ovarian cancer†	82 (0.3)	411 (0.2)
	Prostate cancer‡	678 (2.4)	5230 (2.0)
	Pancreatic cyst	192 (0.7)	115 (0.0)
	Rheumatoid arthritis	540 (1.9)	4710 (1.8)
	Systemic lupus erythematosus	24 (0.1)	238 (0.1)
	Multiple sclerosis	72 (0.3)	634 (0.2)
	AIDS/HIV	24 (0.1)	123 (0.0)
	Psoriatic arthritis	75 (0.3)	719 (0.3)
Medications	Insulin	1984 (7.1)	5977 (2.3)
	Sulphonylureas	3805 (13.5)	15 528 (5.9)
	Metformin	5143 (18.3)	24 082 (9.2)
	Alphaglucosidase	158 (0.6)	850 (0.3)
	Meglitinide	121 (0.4)	524 (0.2)
	DPP4-inihibitor	1028 (3.7)	4004 (1.5)
	Thiazolidinedione	952 (3.4)	4551 (1.7)
	GLP-1 agonist	161 (0.6)	782 (0.3)
	SGLT-2 inhibitor	200 (0.7)	718 (0.3)
	Proton-pump inhibitor	17 367 (61.7)	109 837 (42.0)
	Histamine-2 receptor blocker	6526 (23.2)	45 174 (17.3)
	Aspirin	10 985 (39.0)	89 650 (34.3)
	Statin	11 940 (42.4)	100 164 (38.3)
	Bisphosphonate	2642 (9.4)	22 445 (8.6)
	Immunosupressant	230 (0.8)	1597 (0.6)
	Digoxin	1441 (5.1)	10 315 (3.9)

Figures are numbers (%) unless otherwise specified.

*Breast cancer (female population): Overall 6723 (4.6%); PDAC 771 (5.5%); controls 5952 (4.5%).

†Ovarian cancer (female population): Overall 491 (0.3%); PDAC 82 (0.6%); controls 409 (0.3%).

‡Prostate cancer (male population): Overall 5907 (4.1%); PDAC 678 (4.8%); controls 5229 (4.0%).

PDAC, pancreatic ductal adenocarcinoma.

### Temporal trends of BMI and blood-based markers

**Figure 1:**
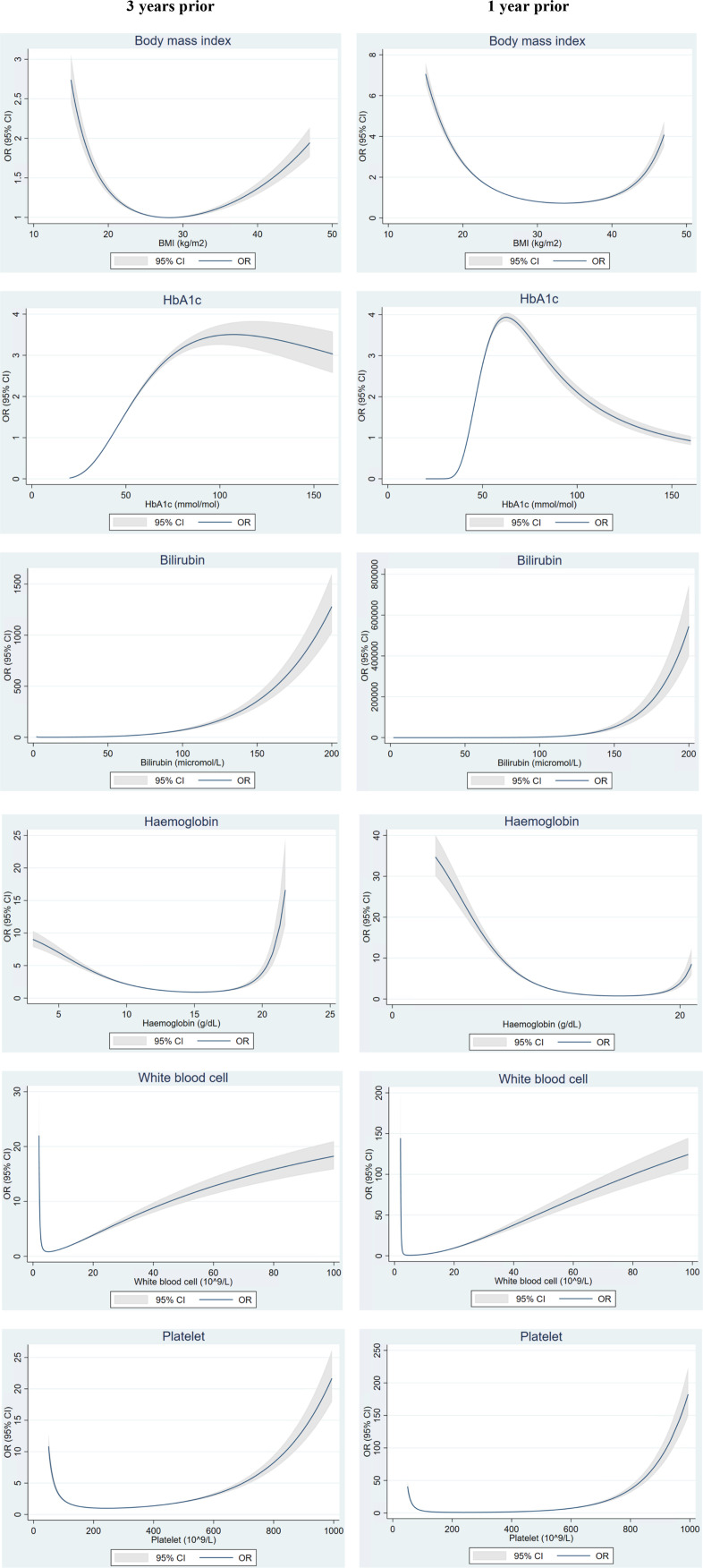
Fractional polynomial plots for the association of BMI and biomarker levels with risk of PDAC up to 3 years vs 1 year prior to index date. BMI, body mass index; HbA1c, haemoglobin A1c; PDAC, pancreatic ductal adenocarcinoma.

Figure 1 shows non-linear relationships between BMI and biomarkers with risk of PDAC. Results showed the risk of PDAC was increased with raised HbA1c, liver markers (online supplemental figure S2a), WCC and platelets. A U-shaped relationship was observed with BMI and Hb; higher risk in patients with low and high values. Generally, trends were similar comparing 3 years prior vs 1 year prior to index date, but magnitude of risks were higher for markers measured closer to the index date, that is, 1 year compared with 3 years prior.

**Figure 2:**
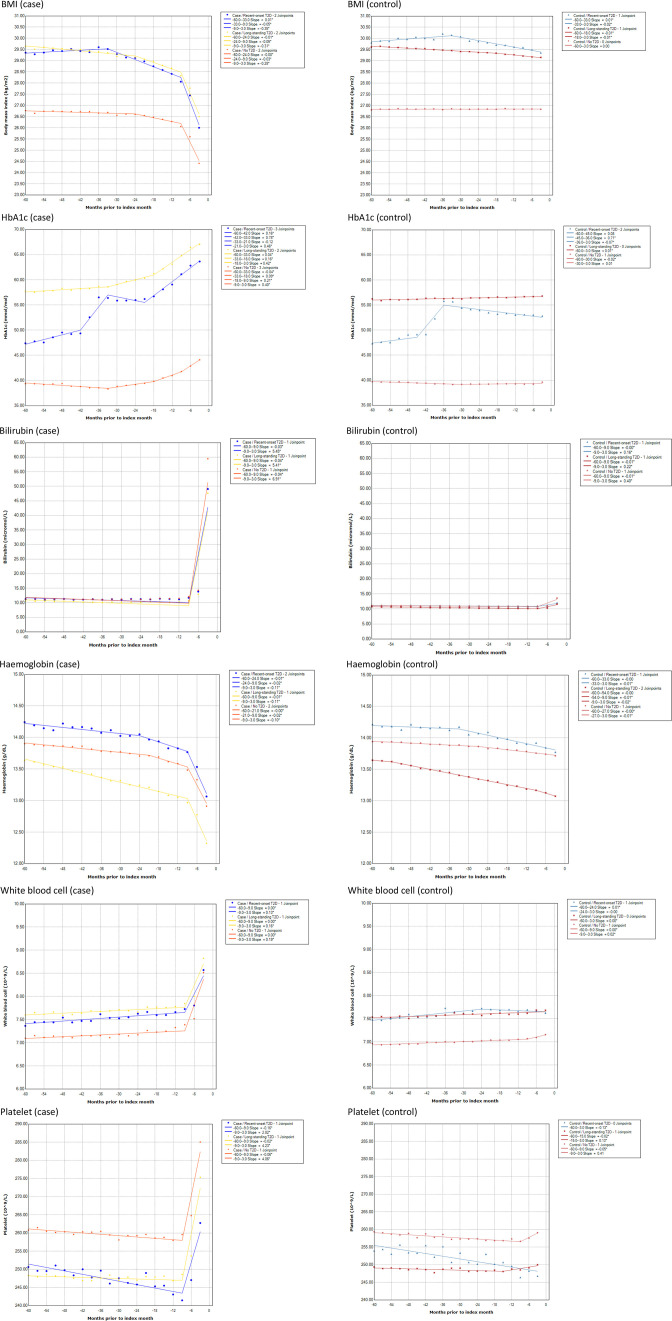
Five-year time trends of mean BMI and blood markers (HbA1c, bilirubin, haemoglobin, white blood cell and platelets) prior to index month in PDAC cases versus controls by type 2 diabetes status (recent onset, long-standing, none). BMI, body mass index; HbA1c, haemoglobin A1c; PDAC, pancreatic ductal adenocarcinoma.

Five-year trends prior to the index month showed evidence of a biphasic decrease in BMI among PDAC cases but not in controls. This differential biphasic decrease in BMI was evident across all three T2D groups—recent onset, long-standing and no T2D; starting with an early gradual decline phase at 33 months (recent-onset T2D) to 24 months prior (long-standing and no T2D), followed by a late rapid decline phase from 9 months prior to the index month regardless of T2D status (figure 2).

Similarly, results supported evidence of at least a biphasic increase in HbA1c among PDAC cases but not in controls. This differential biphasic increase in HbA1c was mostly evident in long-standing and no T2D groups; starting with an early gradual increase phase at 33 months, followed by a late rapid increase phase beginning 18 months prior to the index month. In patients with recent-onset T2D, results showed evidence of increase in HbA1c beginning 42 and 45 months prior followed by a gradual decline for both case and control groups, respectively, likely corresponding to new-diagnosis of T2D and subsequent glucose control measures introduced. However, in the case group with recent-onset T2D, HbA1c began to increase rapidly again 21 months prior to the index month, which contrasted with the continual decline in control group (figure 2).

Liver function markers (ALT, AST, GGT, bilirubin) and blood counts (WBC, platelets) showed a monophasic late rapid increase in PDAC cases but not in controls. This phase began 9 months prior to the index month, and was consistent across early-onset, long-standing and no T2D PDAC cases (figure 1 and online supplemental figure S2b). Hb levels also showed a rapid descent 9 months prior to the index month, although there was some indication of a gradual descent beginning approximately 24 months prior to PDAC diagnosis.

In addition, time trends of mean BMI and blood markers prior to index month for PDAC cases versus controls and their corresponding 95% CIs provided in online supplemental figure S4 further corroborate above findings of when time trends began to separate and do not overlap.

### Temporal associations of comorbidities and risk of PDAC

**Figure 3:**
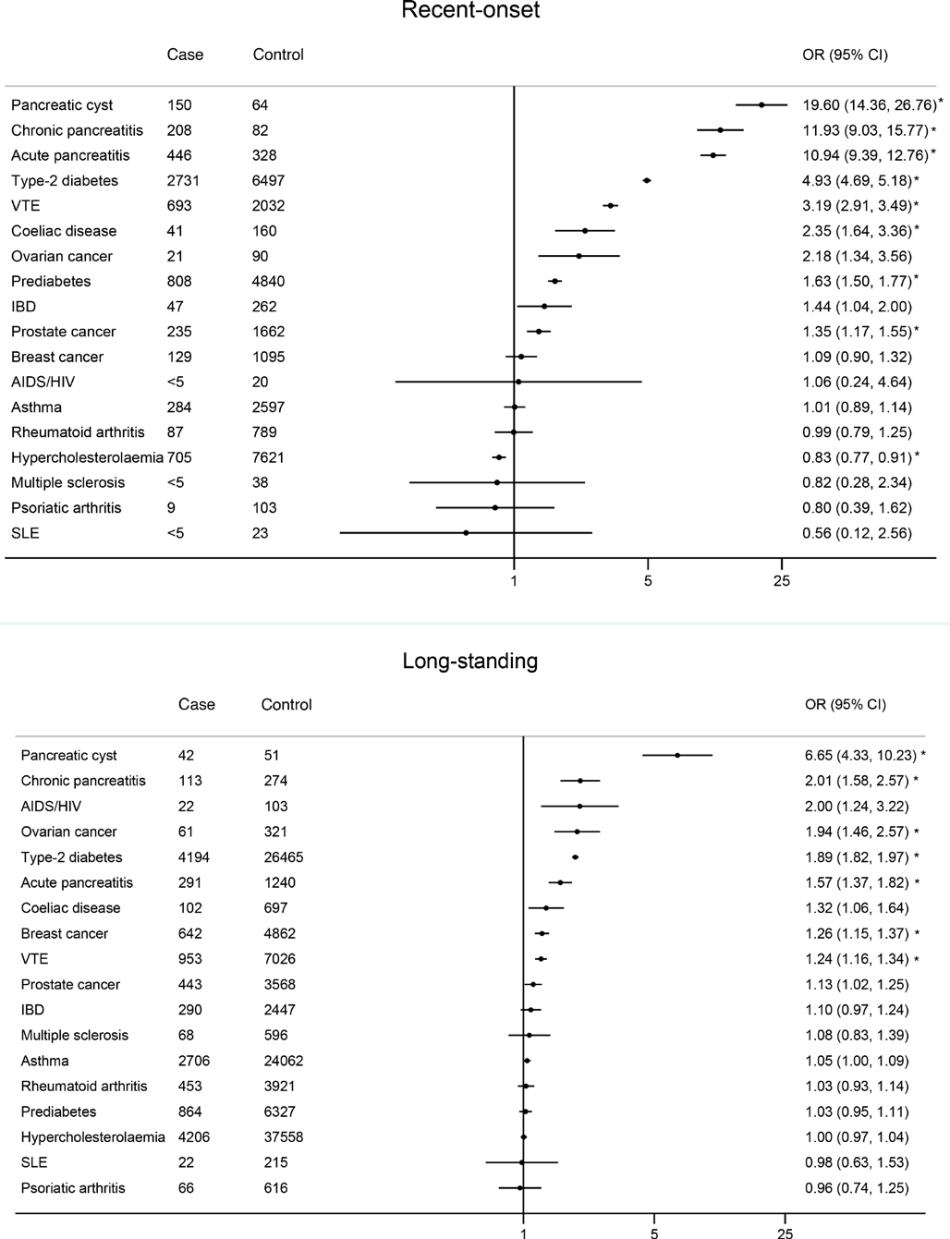
Association of comorbidities with risk of PDAC by onset time; recent onset defined as within 3 years prior to index date, long-standing defined as more than 3 years prior to index date.^a, b a^Estimates are ORs adjusted for ethnicity, deprivation quintile, BMI, smoking and alcohol consumption. Reference group represent those with no corresponding comorbidities. ^b^Associations that remained significant after Bonferonni correction at the 5% level are marked with *(p values are provided in online supplemental table S4). BMI, body mass index; IBD, inflammatory bowel disease; SLE, systemic lupus erythematosus; VTE, venous thromboembolism.

Recent-onset pancreatic cyst (adjusted OR 19.60; 95% CI 14.36 to 26.76), chronic pancreatitis (aOR 11.93; 95% CI 9.03 to 15.77), acute pancreatitis (aOR 10.94; 95% CI 9.39 to 12.76), T2D (aOR 4.93; 95% CI 4.69 to 5.18), venous thromboembolism (VTE) (aOR 3.19; 95% CI 2.91 to 3.49), coeliac disease (aOR 2.35; 95% CI 1.64 to 3.36), ovarian cancer (aOR 2.18; 95% CI 1.34 to 3.56), pre-diabetes (aOR 1.63; 95% CI 1.50 to 1.77), inflammatory bowel disease (aOR 1.44; 95% CI 1.04 to 2.00) and prostate cancer (aOR 1.35; 95% CI 1.17 to 1.55) showed significant associations with increased risks of PDAC diagnosis. These risks remained elevated (although with reduced magnitude) for long-standing pancreatic cyst (aOR 6.65; 95% CI 4.33 to 10.23), chronic pancreatitis (aOR 2.01; 95% CI 1.58 to 2.57), acute pancreatitis (aOR 1.57; 95% CI 1.37 to 1.82), ovarian cancer (aOR 1.94; 95% CI 1.46 to 2.57), T2D (aOR 1.89; 95% CI 1.82 to 1.97), coeliac disease (aOR 1.32; 95% CI 1.06 to 1.64), VTE (aOR 1.24; 95% CI 1.16 to 1.34) and prostate cancer (aOR 1.13; 95% CI 1.02 to 1.25). Other long-standing conditions that showed an association with increased PDAC risk include AIDS/HIV (aOR 2.00; 95% CI 1.24 to 3.22) and breast cancer (aOR 1.26; 95% CI 1.15 to 1.37). These results are presented in figure 3. After Bonferroni correction, increased risk associations that remained significant were (1) recent-onset pancreatic cyst, acute pancreatitis, chronic pancreatitis, T2D, VTE, coeliac disease, pre-diabetes, prostate cancer and (2) long-standing pancreatic cyst, chronic pancreatitis, acute pancreatitis, T2D, VTE, ovarian cancer and breast cancer (figure 3 and online supplemental table S4).

### Temporal associations of medications and risk of PDAC

**Figure 4:**
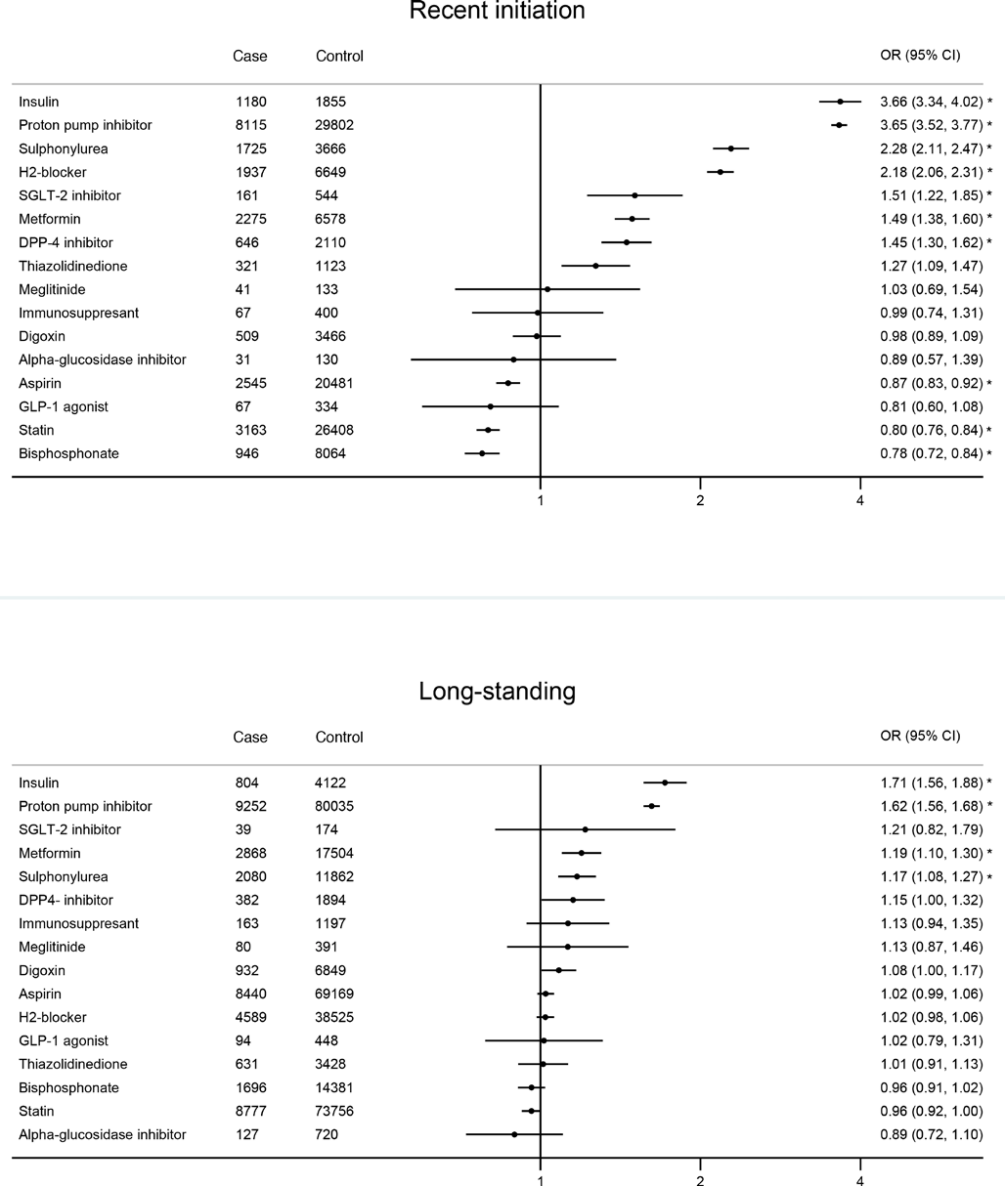
Association of medications with risk of PDAC by initiation time; recent onset defined as within 3 years prior to index date, long-standing defined as more than 3 years prior to index date.^a, b a^Estimates are ORs adjusted for ethnicity, deprivation quintile, BMI, smoking, alcohol consumption and comorbidities. Reference group represent those with no corresponding medication. ^b^Associations that remained significant after Bonferonni correction at the 5% level are marked with *(p values are provided in online supplemental table S4).). BMI, body mass index; PDAC, pancreatic ductal adenocarcinoma.

In terms of medication use, recent initiation of glucose-lowering therapies insulin (aOR 3.66; 95% CI 3.34 to 4.02), sulphonylurea (aOR 2.28; 95% CI 2.11 to 2.47), SGLT-2 inhibitor (aOR 1.51; 95% CI 1.22 to 1.85), metformin (aOR 1.49; 95% CI 1.38 to 1.60), DPP4-inhibitor (aOR 1.45; 95% CI 1.30 to 1.62) and thiazolidinedione (aOR 1.27; 95% CI 1.09 to 1.47) were associated with increased risk of PDAC. Recent initiation of acid-regulating therapies PPI (aOR 3.65; 95% CI 3.52 to 3.77) and histamine-2 receptor blocker (aOR 2.18; 95% CI 2.06 to 2.31) were also associated with increased risk of PDAC. These risks remained elevated (although with reduced magnitude) in patients with long-standing initiation of insulin (aOR 1.71; 95% CI 1.56 to 1.88), sulphonylurea (aOR 1.17; 95% CI 1.08 to 1.27), metformin (aOR 1.19; 95% CI 1.10 to 1.30) and PPI (aOR 1.62; 95% CI 1.56 to 1.68). These results are presented in figure 4. After Bonferroni correction, increased risk associations that remained significant were (1) recent initiation of glucose lowering therapies (insulin, sulphonylurea, metformin, SGLT-2 inhibitors, DPP4-inhibitors) and acid-regulating therapies (proton pump inhibitors, histamine-2 blockers) and (2) long-standing use of insulin, metformin, sulphonylurea and proton pump inhibitors (figure 4 and online supplemental table S4).

## Sensitivity analyses

We performed sensitivity analyses for the joinpoint analysis using complete-case data and results generally showed similar trends compared with the multiply imputed analysis with few differences; lesser number of joinpoint changes in the following markers/subgroups: BMI in recent-onset T2D, HbA1c in long-standing T2D, Hb in no T2D and WBC showed a linear increase across all T2D subgroups (sensitivity analysis 1: online supplemental figure S3).

In addition, we evaluated 5-year time trends of BMI and blood markers using fractional polynomial terms with corresponding 95% CI. Longitudinal trends were similar compared with the main findings using joinpoint regression for both multiply imputed data as well as complete-case data (sensitivity analysis 2a–b: online supplemental figure S4 and S5).

Further sensitivity analysis for comorbidities and medications using a definition of recent-onset or initiation as 2 years prior to index date showed similar associations to the main analysis, with mainly a marginal increase in effect size for onset of comorbidities and initiation of medications in recent 2 years prior to index date (sensitivity analysis 3a–b: online supplemental figure S6 and S7).

Post hoc subgroup analysis by age showed stronger associations of risk factors with PDAC in the younger population (age<60 years), particularly with recent-onset pancreatic cyst, pancreatitis, ovarian cancer and VTE (online supplemental figure S8a, b). Subgroup associations by sex and year of diagnosis showed little difference (online supplemental figure S9 and S10a, b). Further subgroup analyses by BMI, smoking status and T2D status showed broadly similar risk associations except although there might be a weak suggestion that magnitude of some risk factors (eg, pancreatic cyst) were mostly stronger in individuals with lower BMI, non-smokers and without T2D (online supplemental figure S14-S19a, b).

Lastly, we computed E-values^39 40^ reported in online supplemental table S3 for all risk factors that were found to be statistically significant. Many risk factors showed an E-value of above 2, except for a few risk factors which showed an E-value of less than two which could be more susceptible to residual confounding (online supplemental table S3).

## Discussion

In this large nested case–control study, we identified risk associations and 5-year time trends in BMI and widely tested blood-based markers that differed between those that developed and did not develop PDAC. Risk of PDAC increased with raised HbA1c, liver markers, WBC and platelets, while following a U-shaped relationship for BMI and Hb.

A U-shaped relationship between PDAC and BMI suggests that high BMI could be a risk factor for PDAC while low BMI potentially manifests subclinical PDAC, that is, weight loss prior to cancer diagnosis, which became more evident 1 year prior compared with 3 years prior. The increased risk of PDAC with raised Hb 3 years prior might be related to other non-PDAC conditions, while increased risk of PDAC with low Hb levels 1 year prior could be suggestive of an anaemic state related to PDAC. However, risks associated with PDAC up to 3 years prior to diagnosis remain considerable for most markers and may serve as a window of opportunity for early detection. Across T2D status, BMI and HbA1c derange biphasically beginning with early gradual changes 2–3 years prior, followed by late rapid changes 1–2 years prior to PDAC diagnosis. Liver markers and blood counts (WBC, platelets) derange monophasically approximately 1 year prior to PDAC. We also noted that risk associations towards extreme values of some blood markers varied for small number of patients—which may potentially involve other, non-PDAC disease aetiologies or factors.

While our findings largely align with and extend epidemiological evidence regarding medical conditions associated with increased PDAC risk, for medication use it remains challenging to eliminate indication bias and reverse causation. For example, the association for some of the medications with recent initiation may be related to reserve causation, that is, use of glucose-lowering therapies may indicate deteriorating glucose control potentially driven by underlying developing PDAC, or use of acid-regulating agents may be partly driven by misattribution to reflux of upper gastrointestinal symptoms predicated by a developing cancer.^9^

Besides confirming previously known associations between pancreatic cysts, pancreatitis, T2D, VTE and breast cancer on PDAC risk,^6 7 10^ we identified associations with *BRCA*-related cancers, that is, ovarian and prostate cancer, which to the best of our knowledge, is the first time this has been identified in a population level study. These findings build on earlier research suggesting possible shared links of *BRCA* mutations in pancreatic cancer^43^ with those in ovarian, breast and prostate cancer.^44–46^ In addition, it is also important to note that our study further confirms the association between coeliac disease and increased PDAC risk where a recent Swedish study found about a 2-fold increased risk of pancreatic cancer which persisted after 1 year of coeliac disease diagnosis.^47^

Strengths of our study include the size of the population-level dataset, and the use of individual-level linkages across several national data sources which benefitted case ascertainment and accurate identification of relevant exposures and confounders. However, as with other observational studies, our study is potentially susceptible to residual confounding from variables that we have no records of that is, information on former alcohol consumption as well as duration of smoking and alcohol consumption. Large E-values imply a relatively large unmeasured confounding effect is needed to explain away residual confounding, while small E-values imply small unmeasured confounding effect is needed to explain away any residual confounding.^39 40^ Most significant risk factors reported in our study showed an E-value of above 2, except for a few risk factors which showed an E-value of less than two which might be more susceptible to residual confounding. Further, our study is less likely to be susceptible to selection bias as we have used a large and representative population-based primary care routinely collected database. We minimised ascertainment bias of PDAC cases by using cases captured across linked databases that is, primary care, cancer registry, hospital and death records.

Other limitations include the fact that individual coefficients in the models do not have a causal interpretation (indeed, we suggest that our results may inform associative risk prediction tools), and the risk of misascertainment of conditions such as pre-diabetes which may be underdiagnosed in the wider population as they are not routinely screened for. Another limitation includes the unavailability of genomic data in current electronic health records to ascertain *BRCA* status and other genomic markers of pancreatic, ovarian, breast and prostate cancers, which could be an important risk factor for pancreatic cancer for future study. Finally, we were also not able to evaluate potential time-varying nature of exposures using a case–control design. As more pancreatic cancer cases accrue with time, we may be able to evaluate this prospectively in a future study using a cohort study design. In future studies, it will be interesting to assess whether the biomarker trends associated with PDAC found in this study are unique to PDAC compared with other cancers.

To our knowledge, this study is the first to describe the longitudinal trends in widely used biochemical markers, clearly quantifying derangement time and magnitude. By identifying derangements in accessible blood markers up to 3 years prior to PDAC diagnosis, and by extending binary exposure–outcome relationships to stratifying by duration, we believe our results provide more robust data for future studies to identify at-risk subpopulations for PDAC. This could then incorporate data regarding time trends of blood-based markers in combination with temporality and duration of comorbidities and prescriptions into multivariable risk prediction tools.

In conclusion, the risk of PDAC increased with raised HbA1c, liver markers, WBC and platelets, while following a U-shaped relationship for BMI and Hb. Some of these derangements in primary care are detectable up to 3 years prior to diagnosis of PDAC. Across T2D status, BMI and HbA1c derange biphasically beginning with early gradual changes 2–3 years prior followed by late rapid changes 1–2 years prior to PDAC diagnosis. Liver markers and blood counts (WBC, platelet) derange monophasically approximately 1 year prior to PDAC. Profiling these in combination with temporality of candidate risk factor comorbidities and medication initiation could be integrated to inform strategies for earlier pancreatic cancer diagnosis.

## Data Availability

To guarantee the confidentiality of personal and health information, only the authors have had access to the data during the study in accordance with the relevant license agreements. Access to QResearch data is according to the information on the QResearch website (www.qresearch.org).
