# Supplementary material for: Temporality of body mass index, blood tests, comorbidities and medication use as early markers for pancreatic ductal adenocarcinoma (PDAC): a nested case–control study
Source: Gut. 2022 Jun 27;72(3):512–21. doi: 10.1136/gutjnl-2021-326522 (PMC9933161; doi:10.1136/gutjnl-2021-326522)
Supplement: Supplementary data [file gutjnl-2021-326522supp001.pdf]

BMJ Publishing Group Limited (BMJ) disclaims all liability and responsibility arising from any reliance placed on this supplemental material which has been supplied by the author(s)

Supplement

Table S1: Completeness of biomarker values used in five-year time trend analysis for each observation time period reported in number of observations (n) and percent (%) of total cohort (n=289,356)

Months prior to index month	BMI	%	HbA1c	%	ALT	%	AST	%	GGT	%	Bilirubin	%	Hb	%	WBC	%	Platelet	%
0 to <3	36,370	12.57	25,873	8.94	50,519	17.46	9,505	3.28	17,719	6.12	55,465	19.17	55,435	19.16	55,335	19.12	55,280	19.10
3 to <6	34,966	12.08	24,253	8.38	41,931	14.49	7,826	2.70	13,695	4.73	45,927	15.87	45,615	15.76	45,485	15.72	45,434	15.70
6 to <9	34,395	11.89	23,353	8.07	39,575	13.68	7,245	2.50	13,079	4.52	43,398	15.00	42,471	14.68	42,376	14.64	42,303	14.62
9 to <12	33,805	11.68	22,174	7.66	38,202	13.20	7,186	2.48	12,556	4.34	42,150	14.57	41,314	14.28	41,214	14.24	41,155	14.22
12 to <15	33,021	11.41	21,546	7.45	37,010	12.79	7,101	2.45	12,500	4.32	40,960	14.16	39,801	13.76	39,728	13.73	39,694	13.72
15 to <18	32,495	11.23	20,645	7.13	36,097	12.47	6,889	2.38	12,276	4.24	39,915	13.79	38,873	13.43	38,793	13.41	38,749	13.39
18 to <21	32,273	11.15	19,803	6.84	35,052	12.11	6,675	2.31	11,989	4.14	38,745	13.39	37,420	12.93	37,336	12.90	37,353	12.91
21 to <24	31,637	10.93	19,070	6.59	34,071	11.77	6,594	2.28	11,726	4.05	37,787	13.06	36,462	12.60	36,423	12.59	36,393	12.58
24 to <27	31,147	10.76	18,409	6.36	33,278	11.50	6,555	2.27	11,588	4.00	37,000	12.79	35,670	12.33	35,622	12.31	35,625	12.31
27 to <30	30,473	10.53	17,368	6.00	32,031	11.07	6,502	2.25	11,188	3.87	35,784	12.37	34,470	11.91	34,425	11.90	34,397	11.89
30 to <33	30,160	10.42	16,930	5.85	31,451	10.87	6,315	2.18	11,042	3.82	35,077	12.12	33,704	11.65	33,644	11.63	33,634	11.62
33 to <36	29,496	10.19	16,066	5.55	30,749	10.63	6,260	2.16	10,966	3.79	34,450	11.91	32,938	11.38	32,860	11.36	32,854	11.35
36 to <39	29,364	10.15	15,661	5.41	29,699	10.26	6,165	2.13	10,605	3.67	33,263	11.50	31,797	10.99	31,772	10.98	31,757	10.98
39 to <42	28,657	9.90	14,783	5.11	28,873	9.98	6,074	2.10	10,487	3.62	32,491	11.23	30,981	10.71	30,964	10.70	30,936	10.69
42 to <45	28,231	9.76	14,127	4.88	28,025	9.69	6,030	2.08	10,127	3.50	31,643	10.94	29,897	10.33	29,867	10.32	29,821	10.31
45 to <48	27,595	9.54	13,563	4.69	27,298	9.43	5,770	1.99	10,004	3.46	30,807	10.65	29,302	10.13	29,274	10.12	29,259	10.11
48 to <51	27,292	9.43	12,998	4.49	26,614	9.20	5,788	2.00	9,824	3.40	30,220	10.44	28,211	9.75	28,206	9.75	28,213	9.75
51 to <54	26,758	9.25	12,471	4.31	25,687	8.88	5,641	1.95	9,705	3.35	29,216	10.10	27,480	9.50	27,477	9.50	27,464	9.49
54 to <57	26,721	9.23	11,983	4.14	24,709	8.54	5,566	1.92	9,182	3.17	28,161	9.73	26,626	9.20	26,573	9.18	26,549	9.18
57 to <60	25,937	8.96	11,068	3.83	23,988	8.29	5,450	1.88	9,129	3.15	27,462	9.49	25,716	8.89	25,666	8.87	25,666	8.87

Tan PS, *et al. Gut* 2023; 72:512–521. doi: 10.1136/gutjnl-2021-326522

Table S2: Onset of comorbidities and initiation time of medication in PDAC cases versus controls

	Onset/initiation time	PDAC cases	Controls
Total, N		28137	261219
**Comorbidities**			
Type 2 diabetes	None	21212 (75.4)	228257 (87.4)
	Start <=3 years ago	2731 (9.7)	6497 (2.5)
	Start >3 years ago	4194 (14.9)	26465 (10.1)
Prediabetes	None	26465 (94.1)	250052 (95.7)
	Start <=3 years ago	808 (2.9)	4840 (1.9)
	Start >3 years ago	864 (3.1)	6327 (2.4)
Acute pancreatitis	None	27400 (97.4)	259651 (99.4)
	Start <=3 years ago	446 (1.6)	328 (0.1)
	Start >3 years ago	291 (1.0)	1240 (0.5)
Chronic pancreatitis	None	27816 (98.9)	260863 (99.9)
	Start <=3 years ago	208 (0.7)	82 (0.0)
	Start >3 years ago	113 (0.4)	274 (0.1)
Hypercholesterolaemia	None	23226 (82.5)	216040 (82.7)
	Start <=3 years ago	705 (2.5)	7621 (2.9)
	Start >3 years ago	4206 (14.9)	37558 (14.4)
Venous thromboembolism	None	26491 (94.2)	252161 (96.5)
	Start <=3 years ago	693 (2.5)	2032 (0.8)
	Start >3 years ago	953 (3.4)	7026 (2.7)
Asthma	None	25147 (89.4)	234560 (89.8)
	Start <=3 years ago	284 (1.0)	2597 (1.0)
	Start >3 years ago	2706 (9.6)	24062 (9.2)
Inflammatory bowel disease	None	27800 (98.8)	258510 (99.0)
	Start <=3 years ago	47 (0.2)	262 (0.1)
	Start >3 years ago	290 (1.0)	2447 (0.9)
Celiac disease	None	27994 (99.5)	260362 (99.7)
	Start <=3 years ago	41 (0.1)	160 (0.1)
	Start >3 years ago	102 (0.4)	697 (0.3)
Breast cancer	None	27366 (97.3)	255262 (97.7)
	Start <=3 years ago	129 (0.5)	1095 (0.4)
	Start >3 years ago	642 (2.3)	4862 (1.9)
Ovarian cancer	None	28055 (99.7)	260808 (99.8)
	Start <=3 years ago	21 (0.1)	90 (0.0)
	Start >3 years ago	61 (0.2)	321 (0.1)
Prostate cancer	None	27459 (97.6)	255989 (98.0)
	Start <=3 years ago	235 (0.8)	1662 (0.6)
	Start >3 years ago	443 (1.6)	3568 (1.4)
Pancreatic cyst	None	27945 (99.3)	261104 (100.0)
	Start <=3 years ago	150 (0.5)	64 (0.0)
	Start >3 years ago	42 (0.1)	51 (0.0)
Rheumatoid arthritis	None	27597 (98.1)	256509 (98.2)
	Start <=3 years ago	87 (0.3)	789 (0.3)
	Start >3 years ago	453 (1.6)	3921 (1.5)
Systemic lupus erythematosus	None	28113 (99.9)	260981 (99.9)
	Start <=3 years ago	<5	23 (0.0)

Tan PS, *et al. Gut* 2023; 72:512–521. doi: 10.1136/gutjnl-2021-326522

	Start >3 years ago	22 (0.1)	215 (0.1)
Multiple sclerosis	None	28065 (99.7)	260585 (99.8)
	Start <=3 years ago	<5	38 (0.0)
	Start >3 years ago	68 (0.2)	596 (0.2)
AIDS/HIV	None	28113 (99.9)	261096 (100.0)
	Start <=3 years ago	<5	20 (0.0)
	Start >3 years ago	22 (0.1)	103 (0.0)
Psoriatic arthritis	None	28062 (99.7)	260500 (99.7)
	Start <=3 years ago	9 (0.0)	103 (0.0)
	Start >3 years ago	66 (0.2)	616 (0.2)
**Medications**			
Insulin	None	26153 (92.9)	255242 (97.7)
	Start <=3 years ago	1180 (4.2)	1855 (0.7)
	Start >3 years ago	804 (2.9)	4122 (1.6)
Sulphonylurea	None	24332 (86.5)	245691 (94.1)
	Start <=3 years ago	1725 (6.1)	3666 (1.4)
	Start >3 years ago	2080 (7.4)	11862 (4.5)
Biguanides	None	22994 (81.7)	237137 (90.8)
	Start <=3 years ago	2275 (8.1)	6578 (2.5)
	Start >3 years ago	2868 (10.2)	17504 (6.7)
Alpha-glucosidase inhibitor	None	27979 (99.4)	260369 (99.7)
	Start <=3 years ago	31 (0.1)	130 (0.0)
	Start >3 years ago	127 (0.5)	720 (0.3)
Meglitinide	None	28016 (99.6)	260695 (99.8)
	Start <=3 years ago	41 (0.1)	133 (0.1)
	Start >3 years ago	80 (0.3)	391 (0.1)
Dipeptidyl-peptidase 4 inhibitor	None	27109 (96.3)	257215 (98.5)
	Start <=3 years ago	646 (2.3)	2110 (0.8)
	Start >3 years ago	382 (1.4)	1894 (0.7)
Thiazolidinedione	None	27185 (96.6)	256668 (98.3)
	Start <=3 years ago	321 (1.1)	1123 (0.4)
	Start >3 years ago	631 (2.2)	3428 (1.3)
Glucagon-like peptide-1	None	27976 (99.4)	260437 (99.7)
	Start <=3 years ago	67 (0.2)	334 (0.1)
	Start >3 years ago	94 (0.3)	448 (0.2)
Sodium-glucose transport protein 2 (SGLT2) inhibitors	None	27937 (99.3)	260501 (99.7)
	Start <=3 years ago	161 (0.6)	544 (0.2)
	Start >3 years ago	39 (0.1)	174 (0.1)
Proton pump inhibitor	None	10770 (38.3)	151382 (58.0)
	Start <=3 years ago	8115 (28.8)	29802 (11.4)
	Start >3 years ago	9252 (32.9)	80035 (30.6)
Histamine-2 receptor blocker	None	21611 (76.8)	216045 (82.7)
	Start <=3 years ago	1937 (6.9)	6649 (2.5)
	Start >3 years ago	4589 (16.3)	38525 (14.7)
Aspirin	None	17152 (61.0)	171569 (65.7)
	Start <=3 years ago	2545 (9.0)	20481 (7.8)
	Start >3 years ago	8440 (30.0)	69169 (26.5)

Tan PS, et al. Gut 2023; 72:512–521. doi: 10.1136/gutjnl-2021-326522

Statin	None	16197 (57.6)	161055 (61.7)
	Start <=3 years ago	3163 (11.2)	26408 (10.1)
	Start >3 years ago	8777 (31.2)	73756 (28.2)
Bisphosphonate	None	25495 (90.6)	238774 (91.4)
	Start <=3 years ago	946 (3.4)	8064 (3.1)
	Start >3 years ago	1696 (6.0)	14381 (5.5)
Immunosuppressant	None	27907 (99.2)	259622 (99.4)
	Start <=3 years ago	67 (0.2)	400 (0.2)
	Start >3 years ago	163 (0.6)	1197 (0.5)
Digoxin	None	26696 (94.9)	250904 (96.1)
	Start <=3 years ago	509 (1.8)	3466 (1.3)
	Start >3 years ago	932 (3.3)	6849 (2.6)

Sex-specific population

	Onset/initiation time	PDAC cases	Controls
Breast cancer (female)	None	13335 (94.5)	125386 (95.5)
	Start <=3 years ago	129 (0.9)	1094 (0.8)
	Start >3 years ago	642 (4.6)	4858 (3.7)
Ovarian cancer (female)	None	14024 (99.4)	130929 (99.7)
	Start <=3 years ago	21 (0.1)	90 (0.1)
	Start >3 years ago	61 (0.4)	319 (0.2)
Prostate cancer (male)	None	13353 (95.2)	124652 (96.0)
	Start <=3 years ago	235 (1.7)	1661 (1.3)
	Start >3 years ago	443 (3.2)	3568 (2.7)

Tan PS, *et al. Gut* 2023; 72:512–521. doi: 10.1136/gutjnl-2021-326522

Table S3: E-values – sensitivity analysis for potential unmeasured confounding of statistically significant risk factors (large E-values imply a relatively large unmeasured confounding effect is needed to explain away residual confounding, whilst small E-values[39,40] imply small unmeasured confounding effect is needed to explain away any residual confounding)

	OR (95% CI)	E-value (point estimate)	E-value (lower CI to span 1)
**Comorbidities**			
**Recent-onset**			
Pancreatic cyst	19.60 (14.36-26.76)	38.69	28.21
Chronic pancreatitis	11.93 (9.03-15.77)	23.35	17.55
Acute pancreatitis	10.94 (9.39-12.76)	21.37	18.27
Type 2 diabetes	4.93 (4.69-5.18)	9.33	8.85
Venous thromboembolism	3.19 (2.91-3.49)	5.83	5.27
Coeliac disease	2.35 (1.64-3.36)	4.13	2.66
Ovarian cancer	2.18 (1.34-3.56)	3.78	2.01
Prediabetes	1.63 (1.50-1.77)	2.64	2.37
Inflammatory bowel disease	1.44 (1.04-2.00)	2.24	1.24
Prostate cancer	1.35 (1.17-1.55)	2.04	1.62
Hypercholesterolemia	0.83 (0.77-0.91)	1.70	1.43
**Long-standing**			
Pancreatic cyst	6.65 (4.33-10.23)	12.78	8.13
Chronic pancreatitis	2.01 (1.58-2.57)	3.43	2.54
AIDS/HIV	2.00 (1.24-3.22)	3.41	1.79
Ovarian cancer	1.94 (1.46-2.57)	3.29	2.28
Type 2 diabetes	1.89 (1.82-1.97)	3.19	3.04
Acute pancreatitis	1.57 (1.37-1.82)	2.52	2.08
Coeliac disease	1.32 (1.06-1.64)	1.97	1.31
Breast cancer	1.26 (1.15-1.37)	1.83	1.57
Venous thromboembolism	1.24 (1.16-1.34)	1.79	1.59
Prostate cancer	1.13 (1.02-1.25)	1.51	1.16
**Medications**			
**Recent-initiation**			
Insulin	3.66 (3.34-4.02)	6.78	6.14
Proton pump inhibitor	3.65 (3.52-3.77)	6.76	6.50
Sulphonylurea	2.28 (2.11-2.47)	3.99	3.64
Histamine-2 receptor blocker	2.18 (2.06-2.31)	3.78	3.54
SGLT-2 inhibitor	1.51 (1.22-1.85)	2.39	1.74
Metformin	1.49 (1.38-1.60)	2.34	2.10
DPP-4 inhibitor	1.45 (1.30-1.62)	2.26	1.92
Thiazolidinedione	1.27 (1.09-1.47)	1.86	1.40
Meglitinide	1.03 (0.69-1.54)	1.21	1.00
Aspirin	0.87 (0.83-0.92)	1.56	1.39
Statin	0.80 (0.76-0.84)	1.81	1.67
Bisphosphonate	0.78 (0.72-0.84)	1.88	1.67
**Long-standing**			
Insulin	1.71 (1.56-1.88)	2.81	2.49
Proton pump inhibitor	1.62 (1.56-1.68)	2.62	2.49
SGLT-2 inhibitor	1.21 (0.82-1.79)	1.71	1.00
Metformin	1.19 (1.10-1.30)	1.67	1.43
Sulphonylurea	1.17 (1.08-1.27)	1.62	1.37

Table S4: Risk factors that remained significant after Bonferroni correction and corresponding p-values

Comorbidities

Bonferroni corrected p-value: 0.05/(18*2) tests = 0.0014

	p
Recent-onset	
Type-2 diabetes	<0.00001
Acute pancreatitis	<0.00001
VTE	<0.00001
Pancreatic cyst	<0.00001
Chronic pancreatitis	<0.00001
Pre-diabetes	<0.00001
Coeliac disease	<0.00001
Hypercholesterolaemia	0.000016
Prostate cancer	0.000050
Long-standing	
Type-2 diabetes	<0.00001
Pancreatic cyst	<0.00001
Acute pancreatitis	<0.00001
VTE	<0.00001
Chronic pancreatitis	<0.00001
Breast cancer	<0.00001
Ovarian cancer	<0.00001

Medications

Bonferroni correction: 0.05/(16*2) tests = 0.0016

Recent initiation	p
Proton-pump inhibitor	<0.00001
Insulin	<0.00001
Histamine-2 blocker	<0.00001
Sulphonylurea	<0.00001
Biguanide (metformin)	<0.00001
Statin	<0.00001
DPP4-inhibitor	<0.00001
Bisphosphonate	<0.00001
Aspirin	<0.00001
SGLT-2 inhibitor	0.000109
Long-standing	
Biguanide (metformin)	0.000046
Proton-pump inhibitor	<0.00001
Insulin	<0.00001
Sulphonylurea	0.000149

Tan PS, *et al. Gut* 2023; 72:512–521. doi: 10.1136/gutjnl-2021-326522

List S1: ICD-10 codes and ICD-0 codes used for PDAC cases

ICD-10 codes:
C250
C251
C252
C253
C257
C258
C259

ICD-O histology codes:
8000
8001
8003
8010
8020
8021
8022
8031
8140
8141
8210
8211
8230
8310
8430
8440
8450
8452
8470
8480
8481
8490
8500
8503
8550
8551
8560
8570

List S2: Medical conditions and medications included as exposure variables in association analyses with risk of PDAC

a. Medical conditions (18)[7,8,19-22]: pancreatic cyst, chronic pancreatitis, acute pancreatitis, type-2 diabetes mellitus (T2D), prediabetes, venous thromboembolism (VTE), hypercholesterolemia breast cancer, ovarian cancer, prostate cancer, asthma, autoimmune conditions (coeliac disease, inflammatory bowel disease (IBD), rheumatoid arthritis, AIDS/HIV, multiple sclerosis, psoriatic arthritis, and systemic lupus erythematosus (SLE))

b. Medications (16)[23-32]: insulin, metformin, sulphonylurea, SGLT2-inhibitor, DPP4-inhibitor, GLP-1 agonist, thiazolidinedione, meglitinide, alpha-glucosidase inhibitor, proton-pump inhibitor (PPI), histamine-2-receptor antagonist (H2RA), immunosuppressant, digoxin, aspirin, statin, and bisphosphonate.

Tan PS, *et al. Gut* 2023; 72:512–521. doi: 10.1136/gutjnl-2021-326522

Figure S1: Directed acyclic graphs (DAGs) for exposures (i) comorbidities and (ii) medications.

(i) Comorbidities

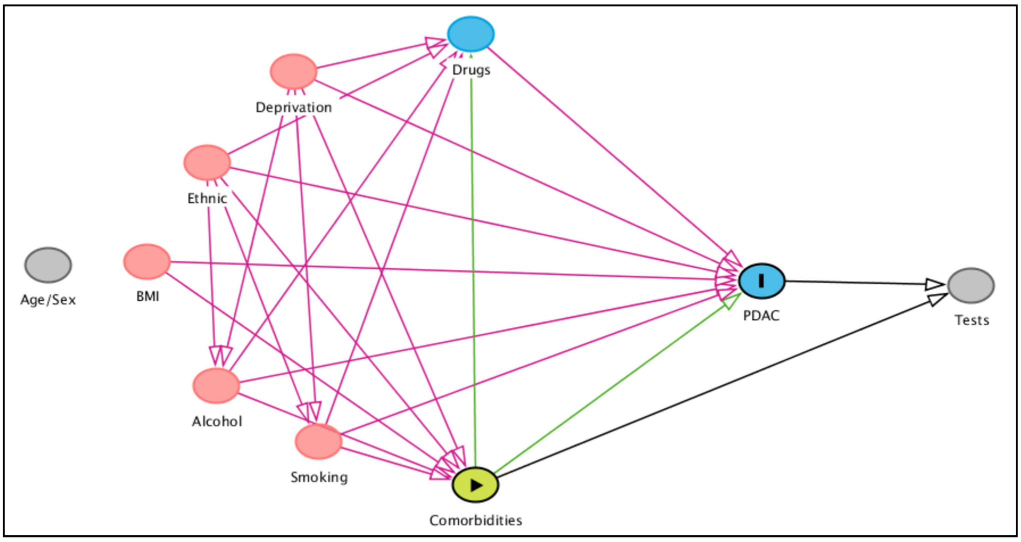

(ii) Medications

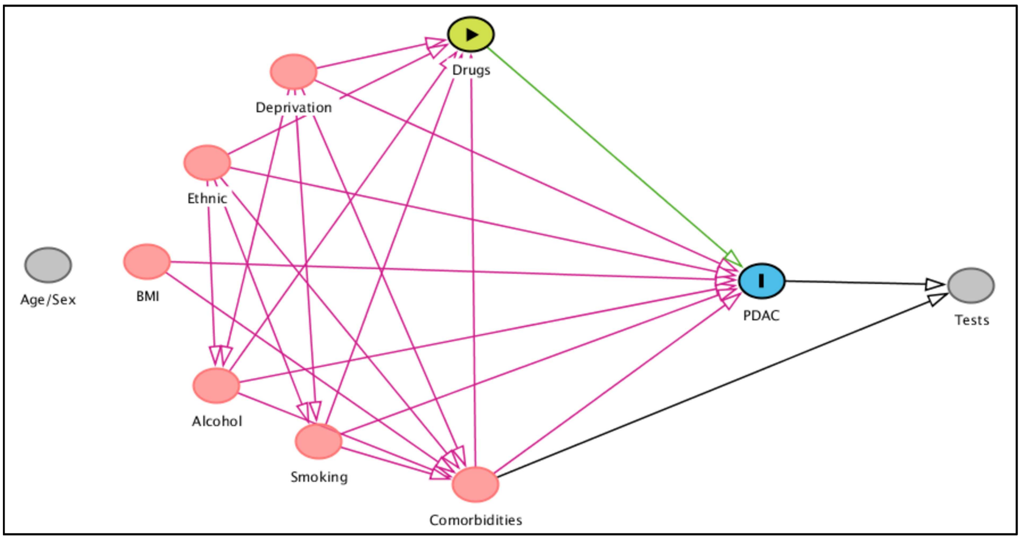

Figure S2 (a): Fractional polynomial plots for the association of BMI and biomarker levels with risk of PDAC up to 3 years versus 1 year prior to index date for other liver function markers (ALT, AST, GGT)

3 years prior | **1 year prior**

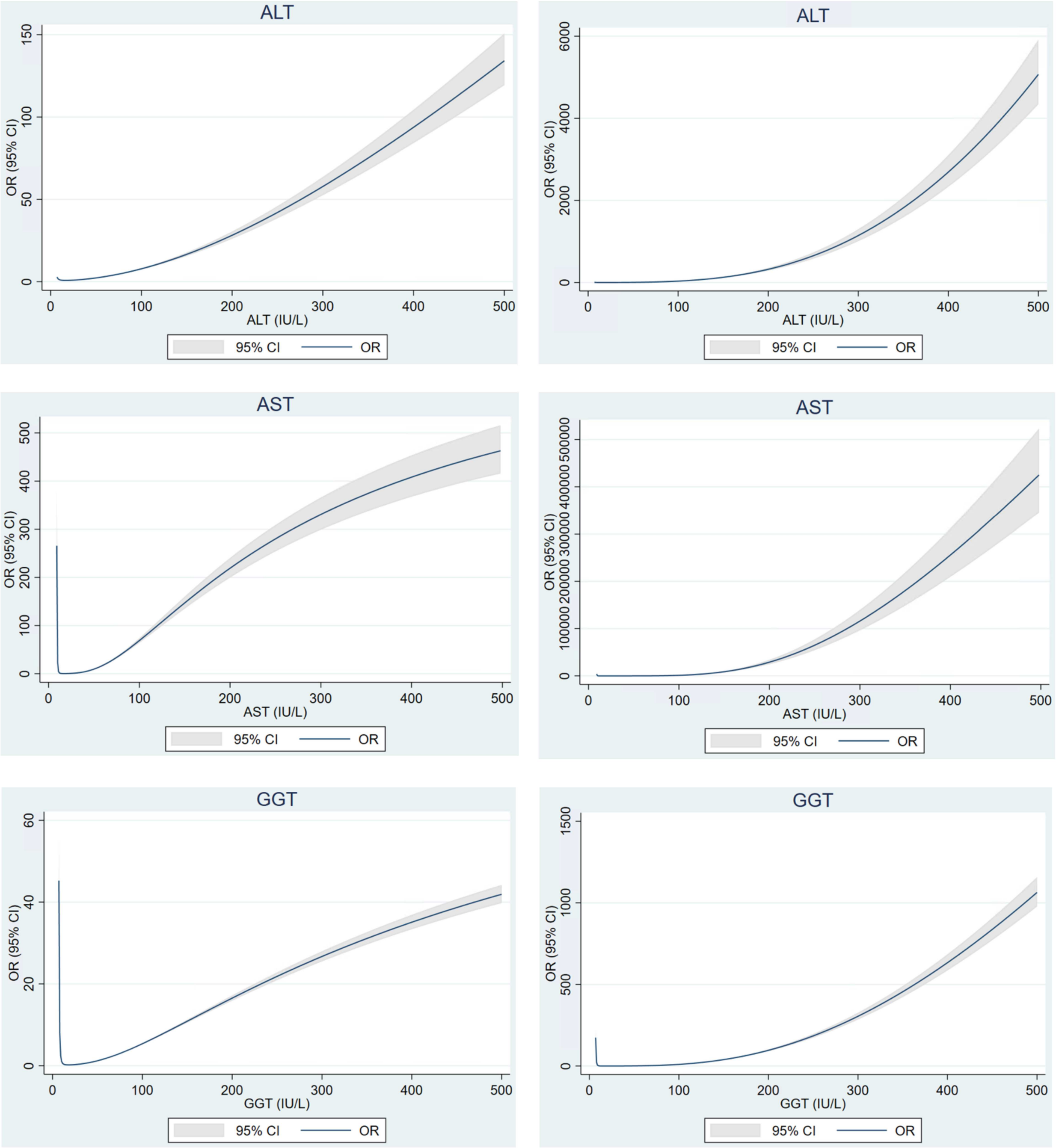

Tan PS, *et al. Gut* 2023; 72:512–521. doi: 10.1136/gutjnl-2021-326522

Figure S2 (b): Five year time-trends of mean liver function markers (ALT, AST, GGT) prior to index month in PDAC cases versus controls by type-2 diabetes status (recent-onset, long-standing, none) (imputed dataset)

ALT (case)

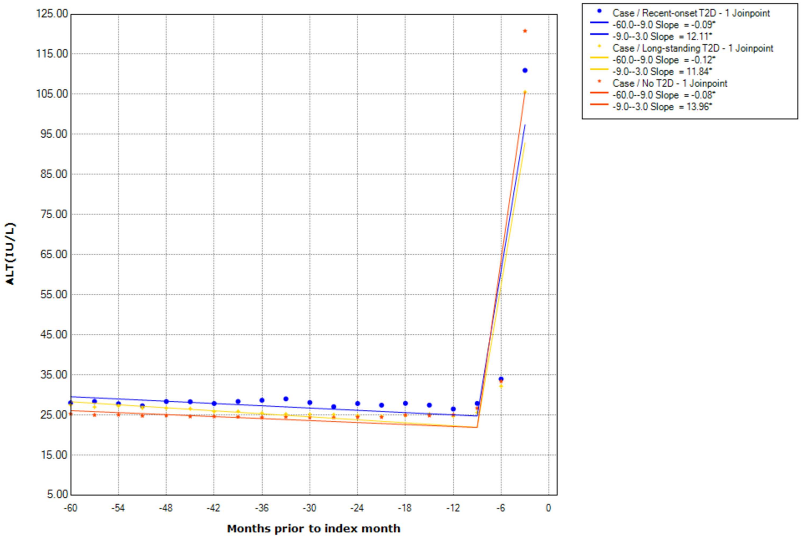

ALT (control)

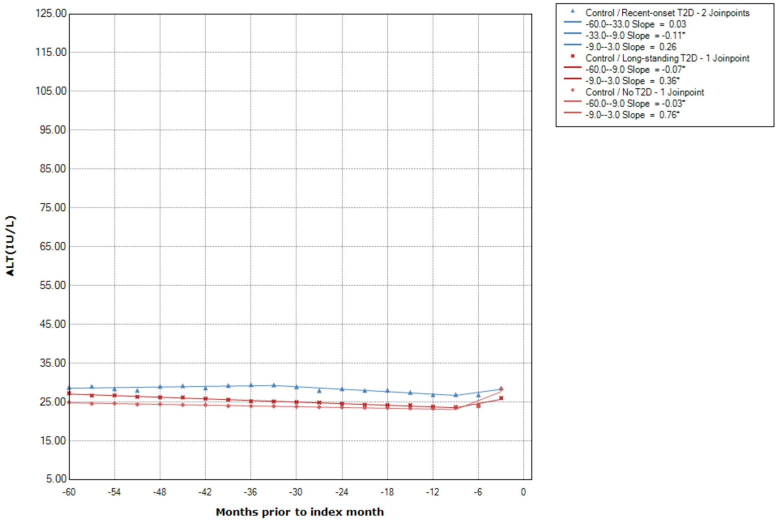

AST (case)

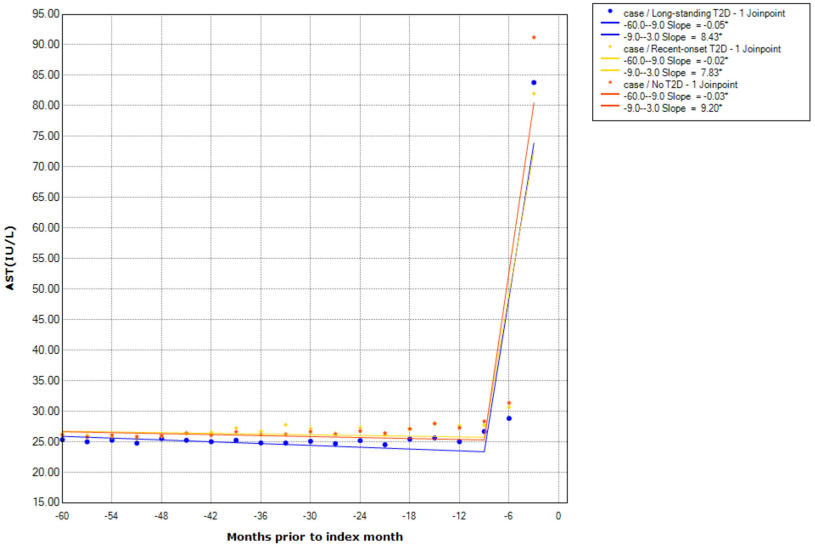

AST (control)

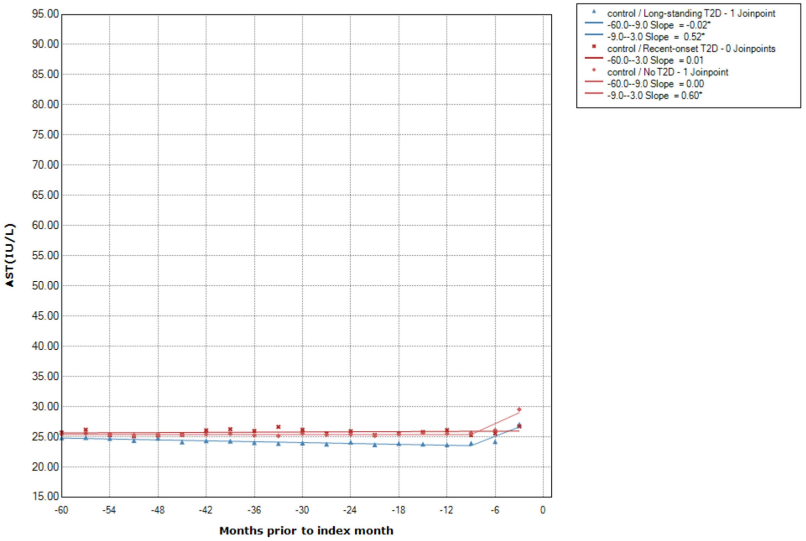

GGT (case)

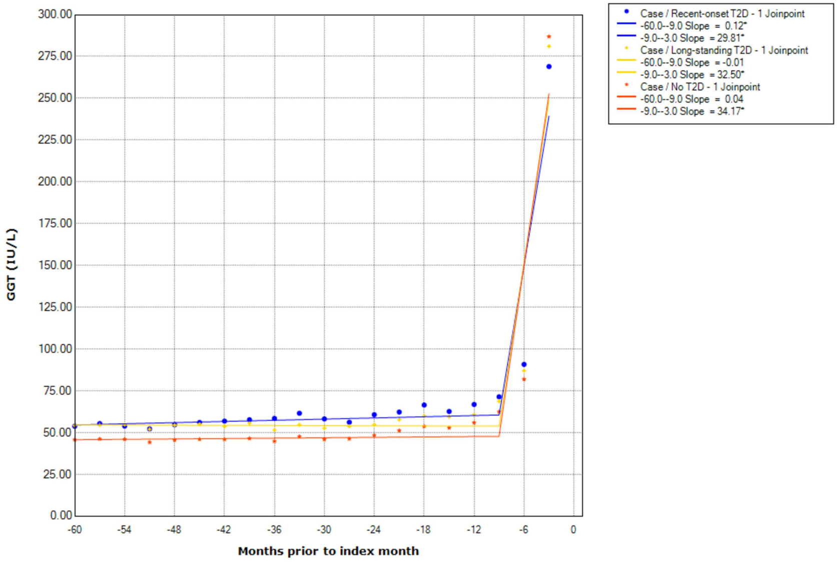

GGT (control)

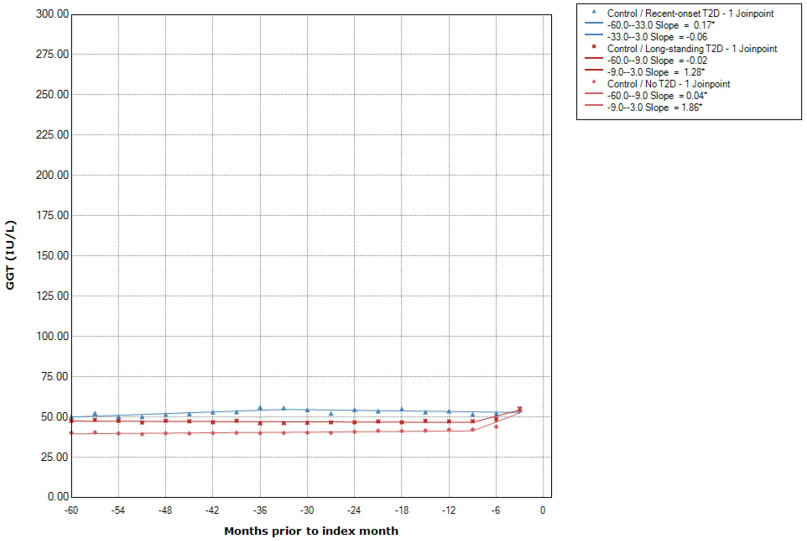

Figure S3: Five year time-trends of mean BMI and blood markers prior to index month in PDAC cases versus controls by type-2 diabetes status (recent-onset, long-standing, none) (complete-case dataset)

BMI (case)

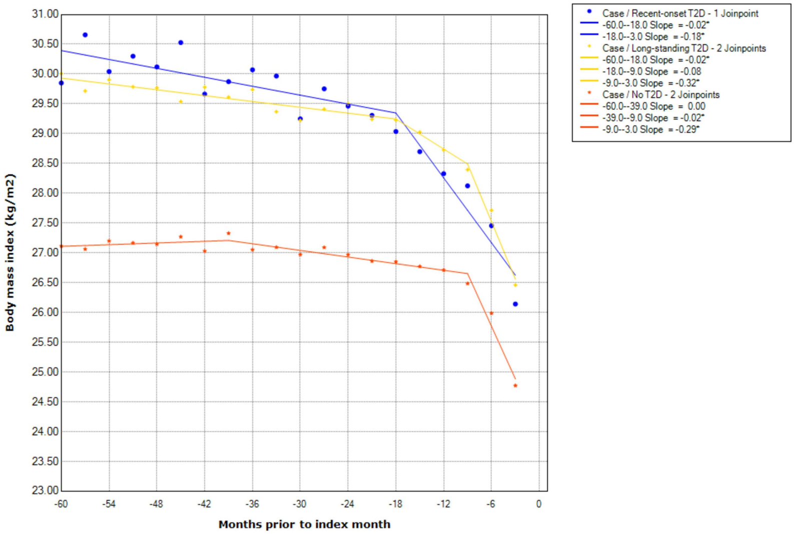

BMI (control)

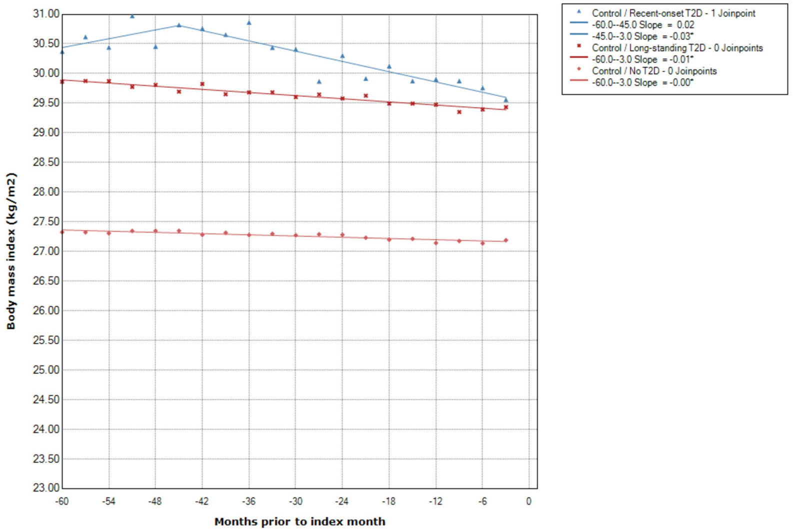

HbA1c (case)

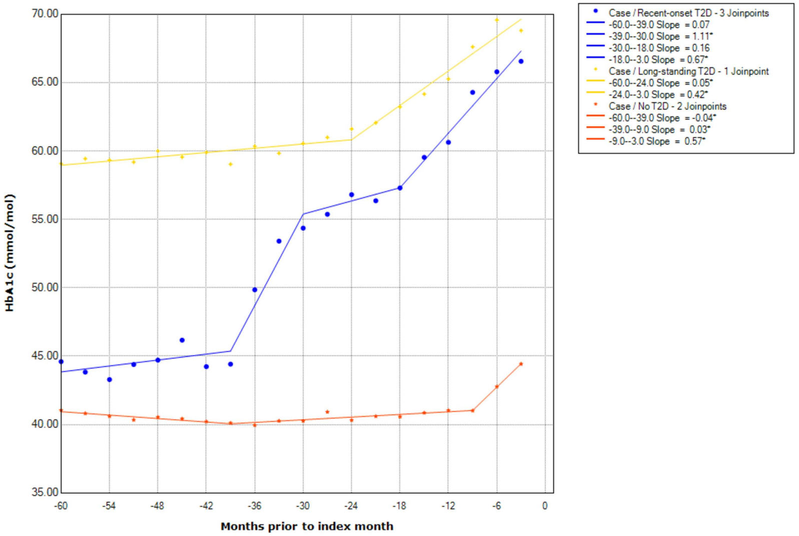

HbA1c (control)

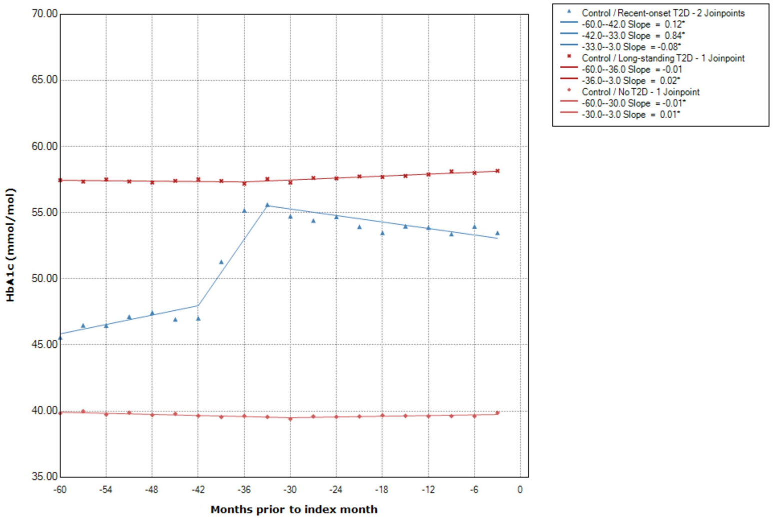

Bilirubin (case)

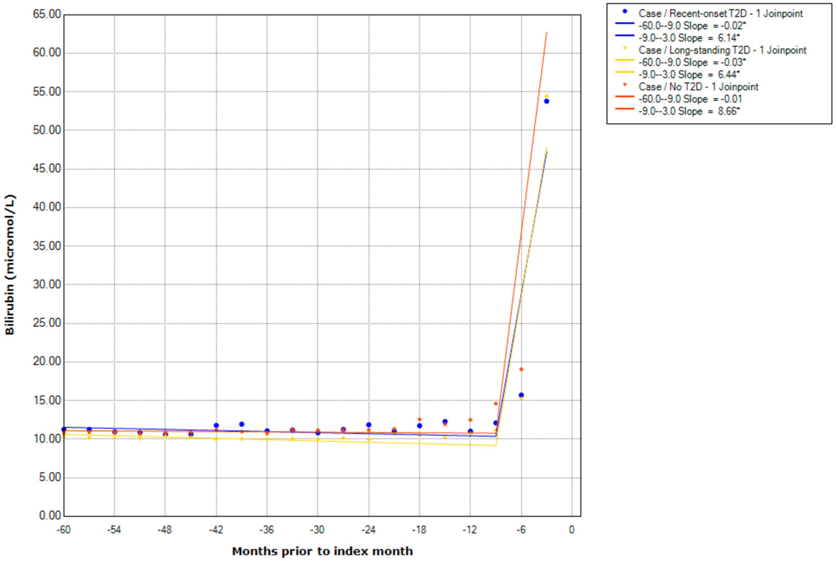

Bilirubin (control)

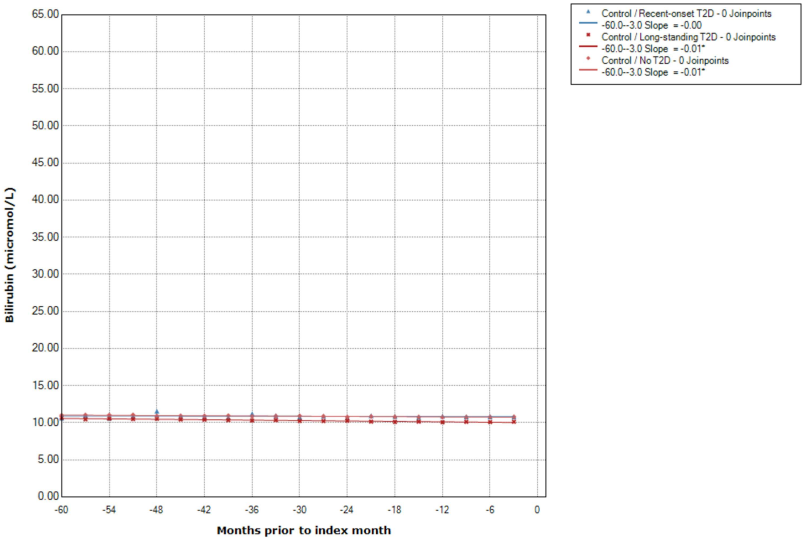

ALT (case)

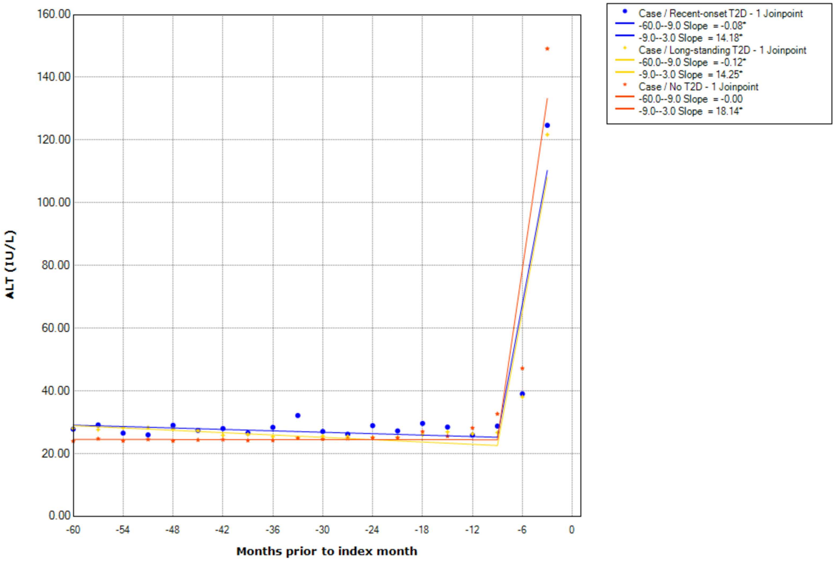

ALT (control)

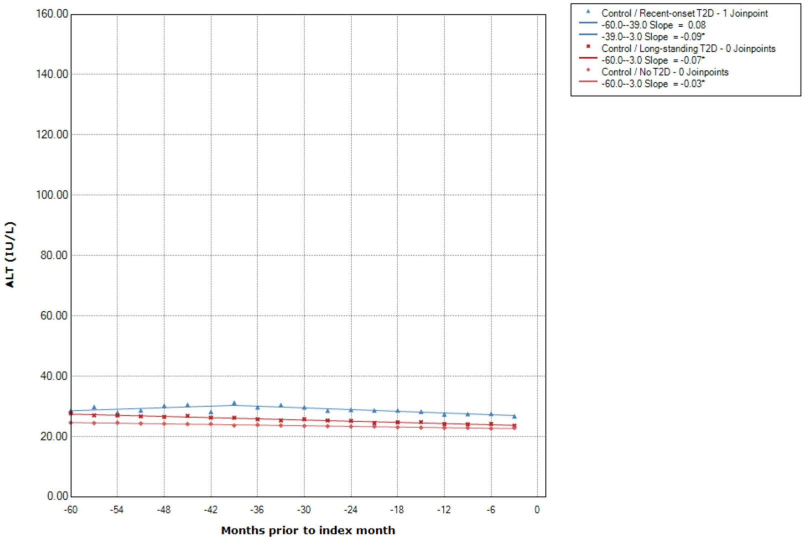

14

AST (case)

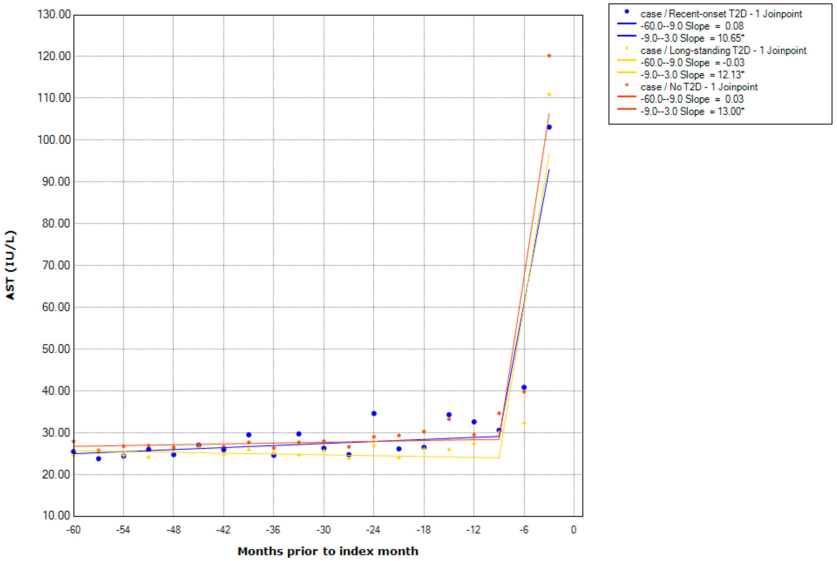

AST (control)

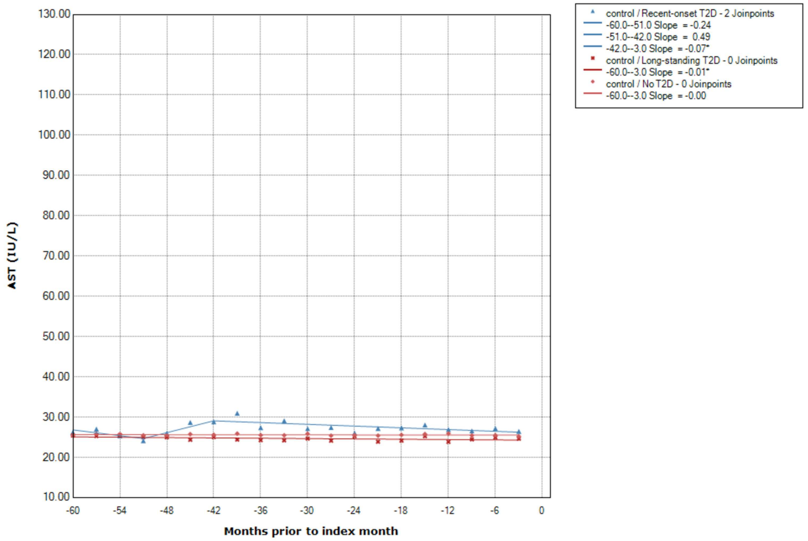

GGT (case)

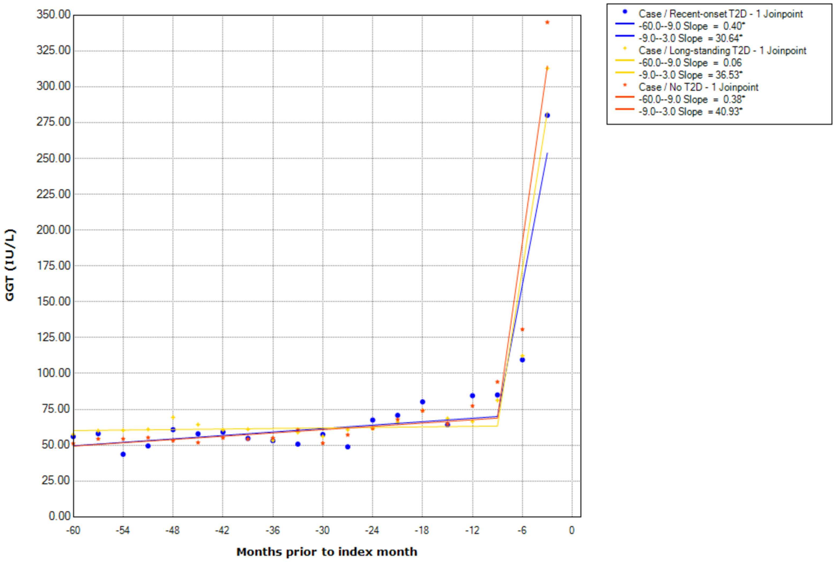

GGT (control)

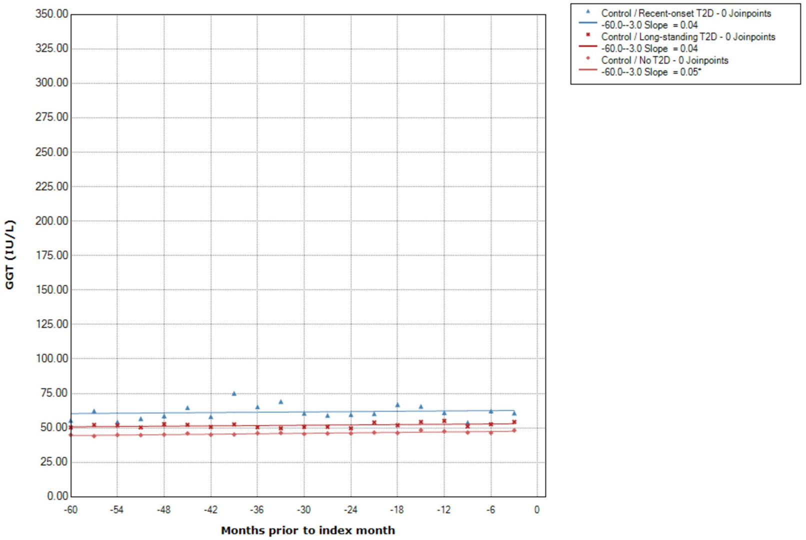

Haemoglobin (case)

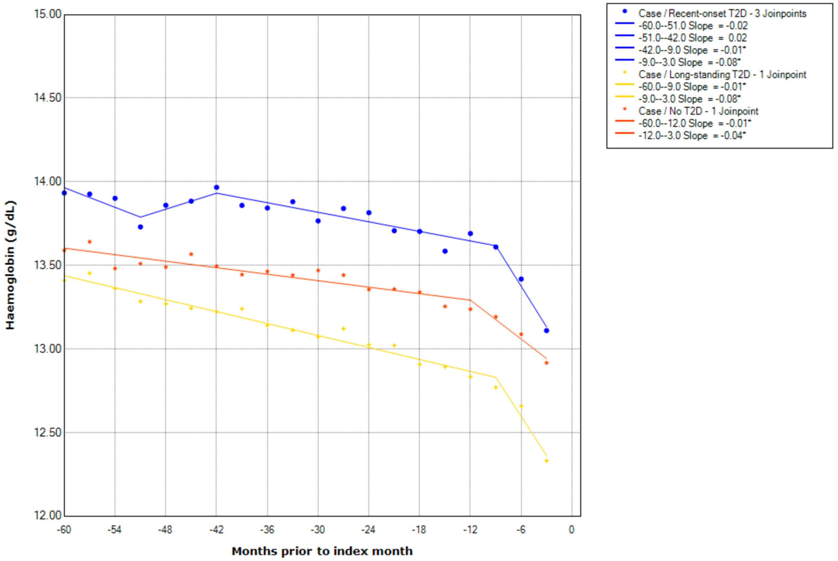

Haemoglobin (control)

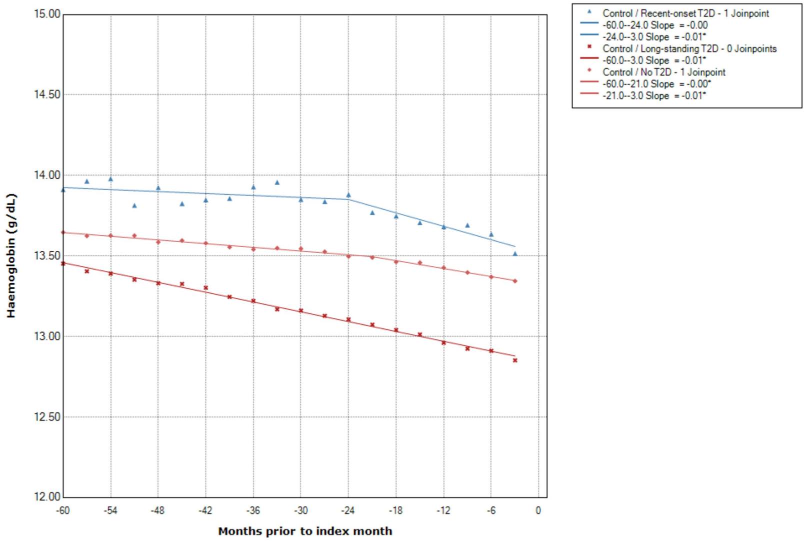

White blood cell (case)

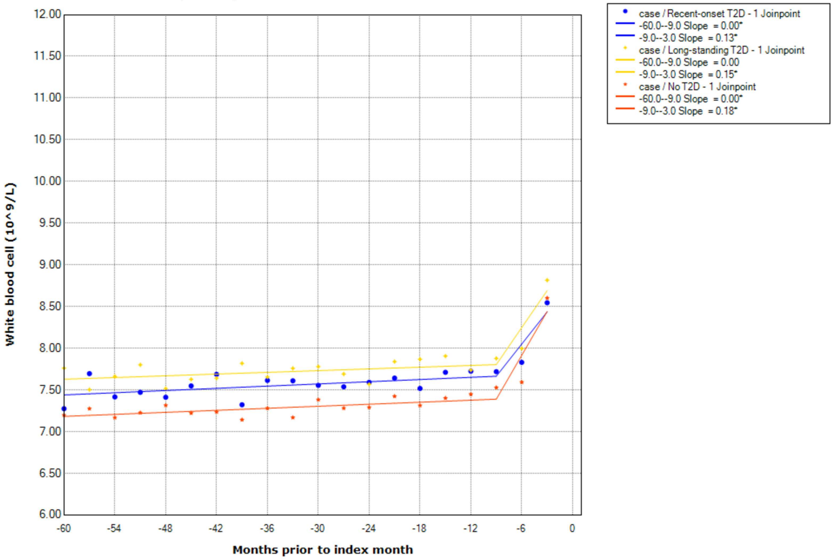

White blood cell (control)

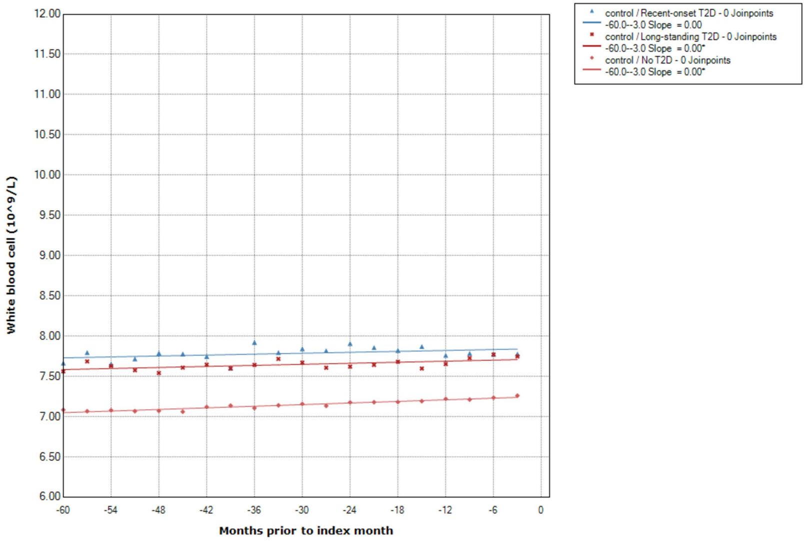

Platelet (case)

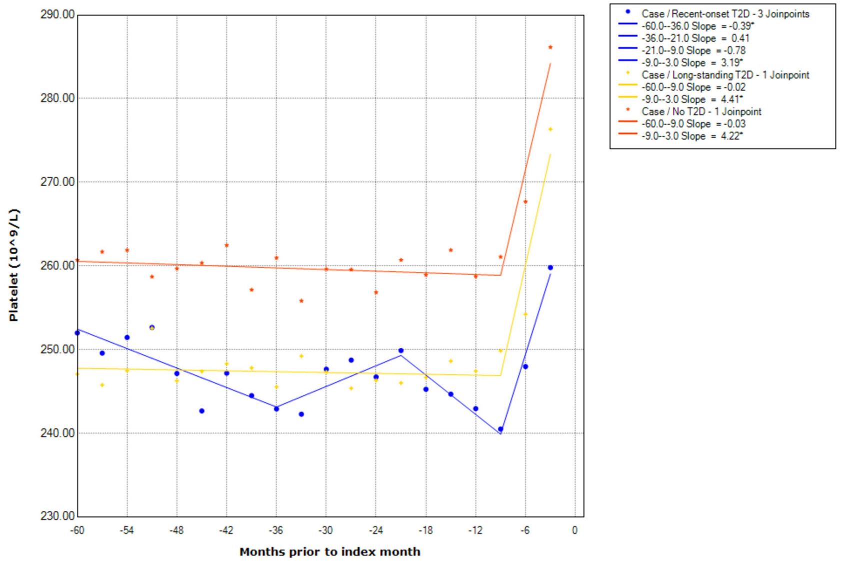

Platelet (control)

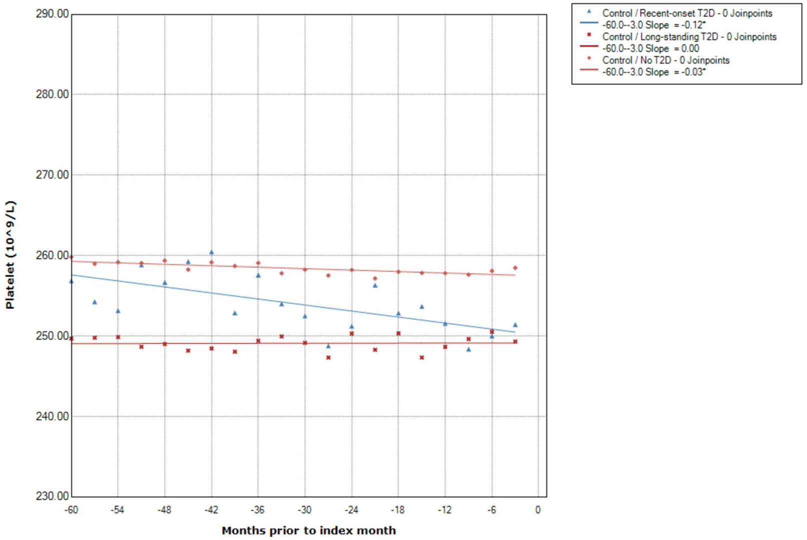

17

Figure S4: Five year time-trends of mean BMI and blood markers prior to index month for PDAC cases/controls by type-2 diabetes status using fractional polynomial fit and corresponding 95% CI (multiply imputed)

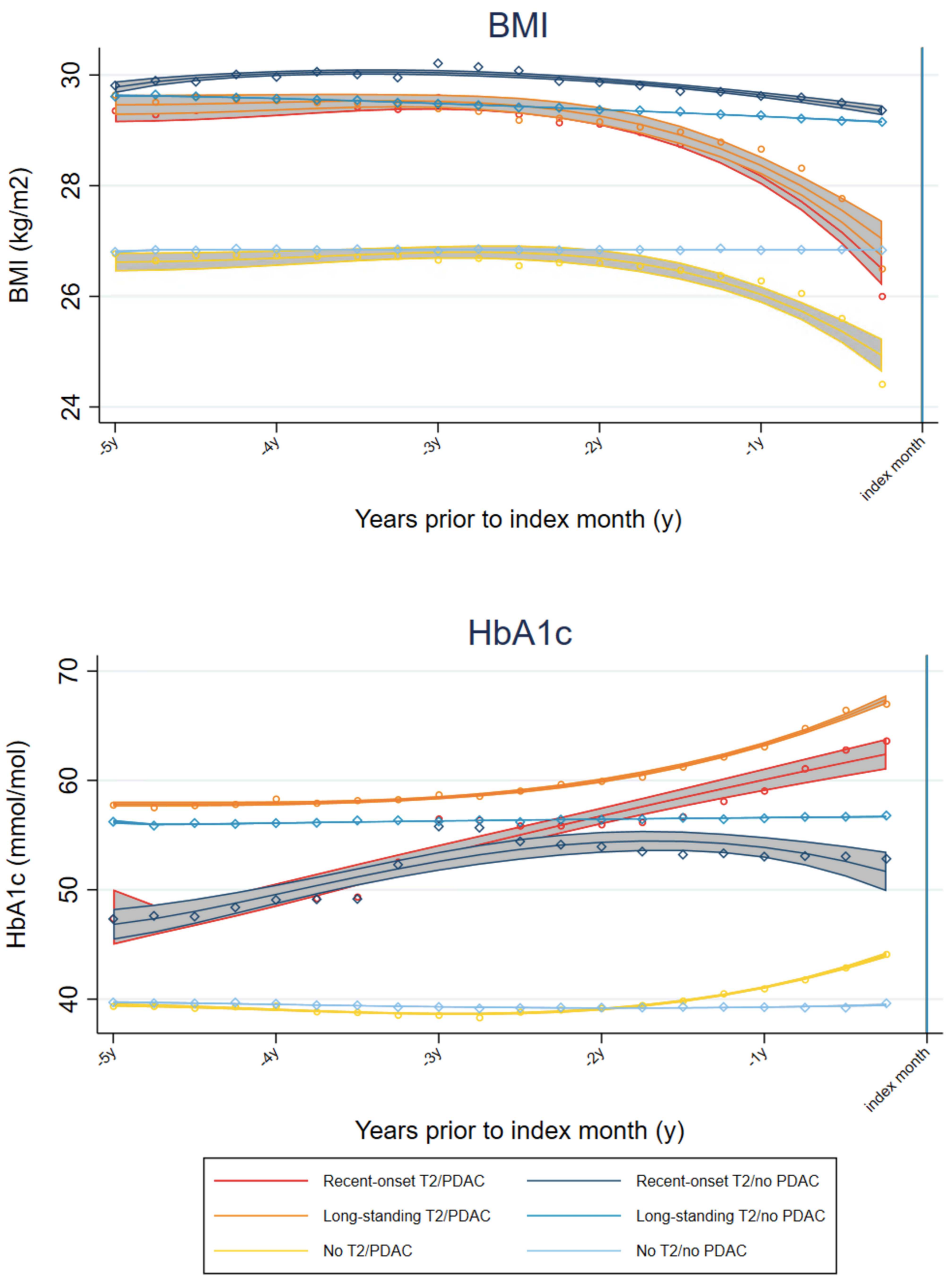

Tan PS, *et al. Gut* 2023; 72:512–521. doi: 10.1136/gutjnl-2021-326522

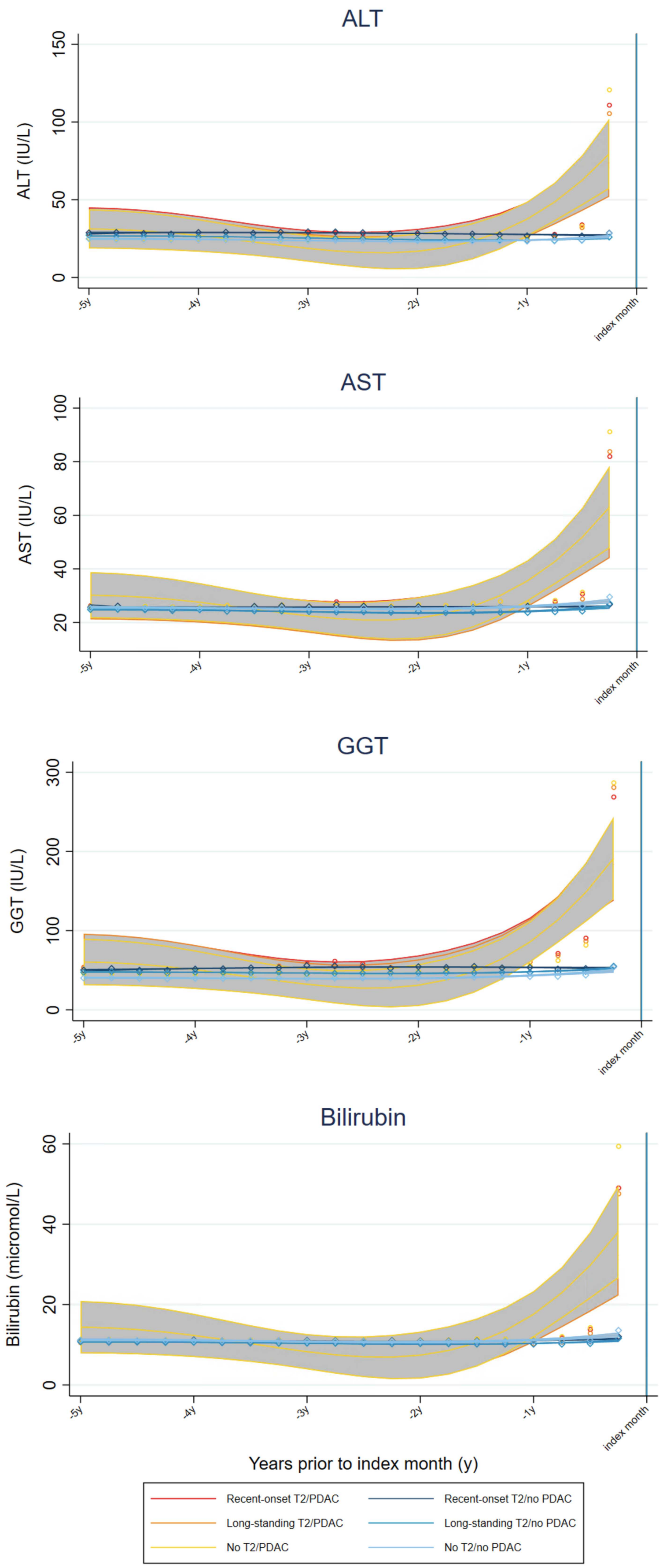
ALT
ALT (IU/L)
0
50
100
150
-5y
-4y
-3y
-2y
-1y
index month
AST
AST (IU/L)
20
40
60
80
100
GGT
GGT (IU/L)
0
100
200
300
Bilirubin
Bilirubin (micromol/L)
0
20
40
60
Years prior to index month (y)
Recent-onset T2/PDAC
Long-standing T2/PDAC
No T2/PDAC
Recent-onset T2/no PDAC
Long-standing T2/no PDAC
No T2/no PDAC

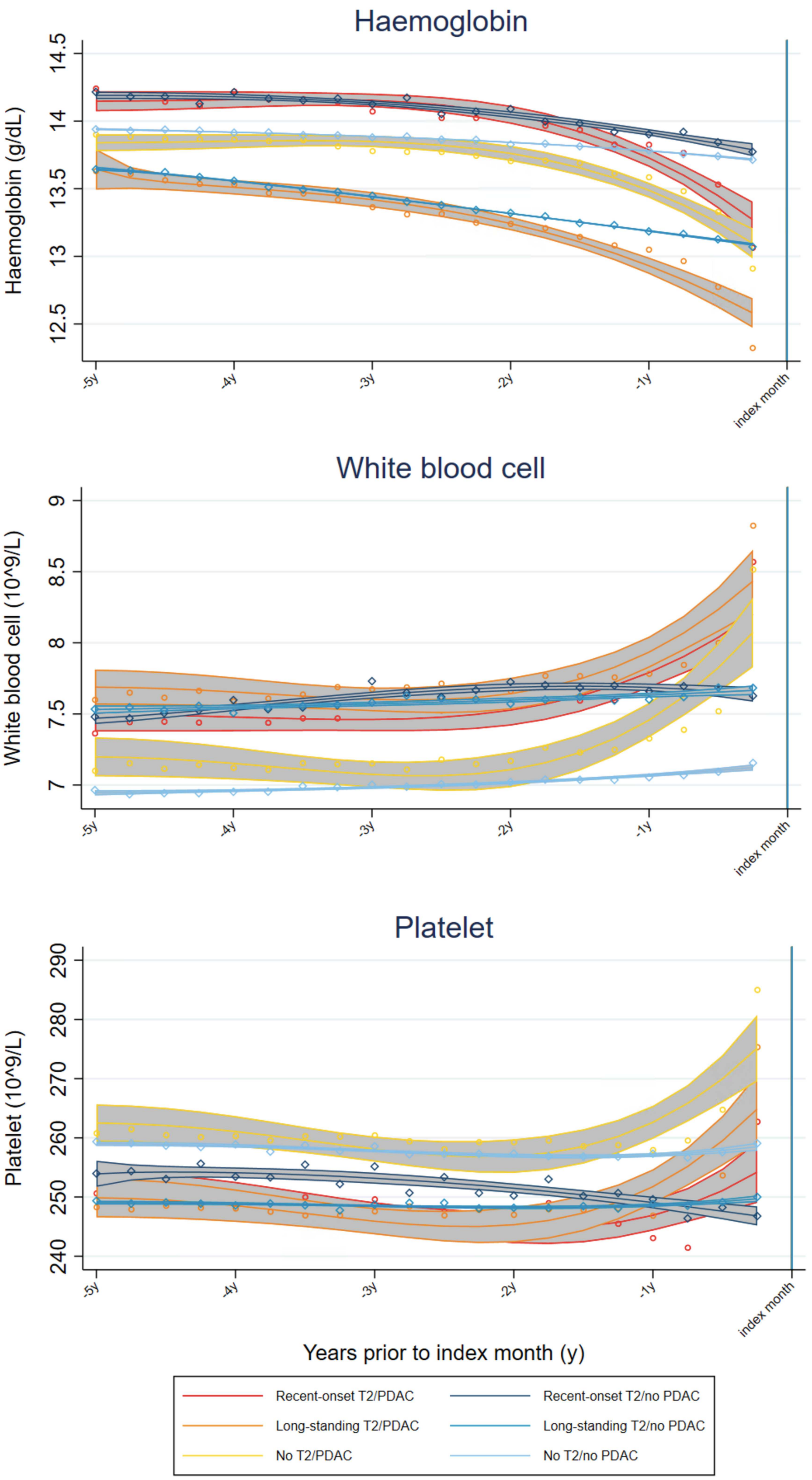

Tan PS, *et al.* *Gut* 2023; 72:512–521. doi: 10.1136/gutjnl-2021-326522

Figure S5: Five year time-trends of mean blood counts (haemoglobin, white blood cell, platelet) prior to index month for PDAC cases/controls by type-2 diabetes status using fractional polynomial fit and corresponding 95% Ci (complete-case)

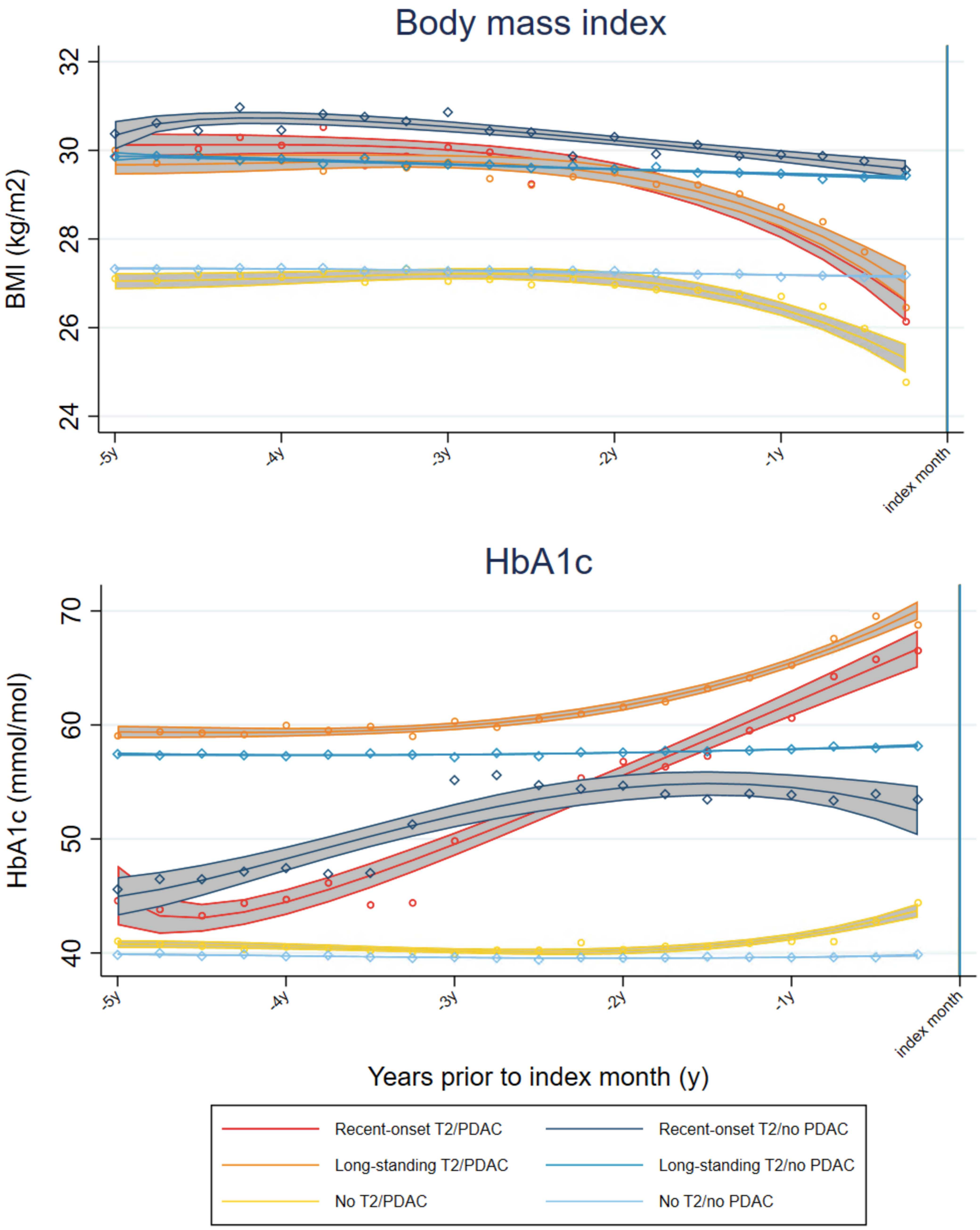

Tan PS, *et al. Gut* 2023; 72:512–521. doi: 10.1136/gutjnl-2021-326522

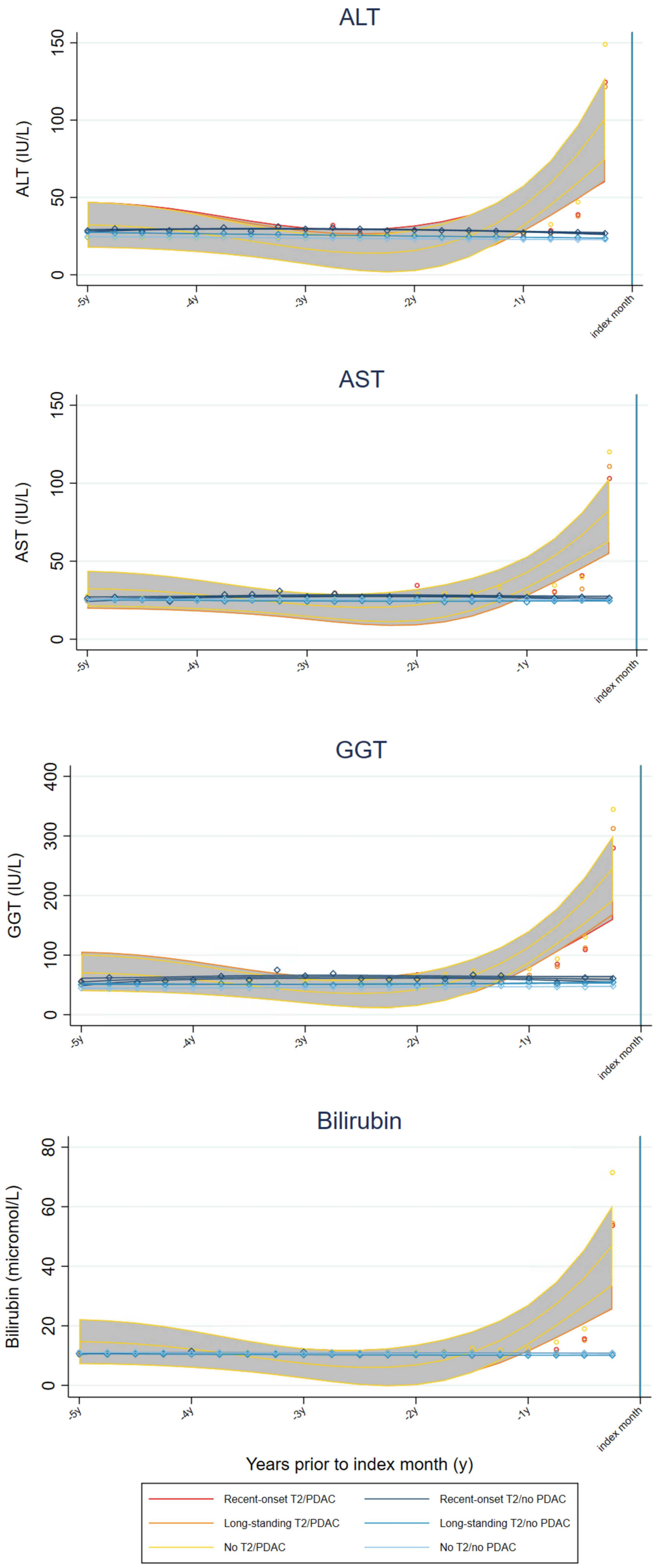
ALT
ALT (IU/L)
AST
AST (IU/L)
GGT
GGT (IU/L)
Bilirubin
Bilirubin (micromol/L)
-5y
-4y
-3y
-2y
-1y
index month
Years prior to index month (y)
Recent-onset T2/PDAC
Long-standing T2/PDAC
No T2/PDAC
Recent-onset T2/no PDAC
Long-standing T2/no PDAC
No T2/no PDAC

22

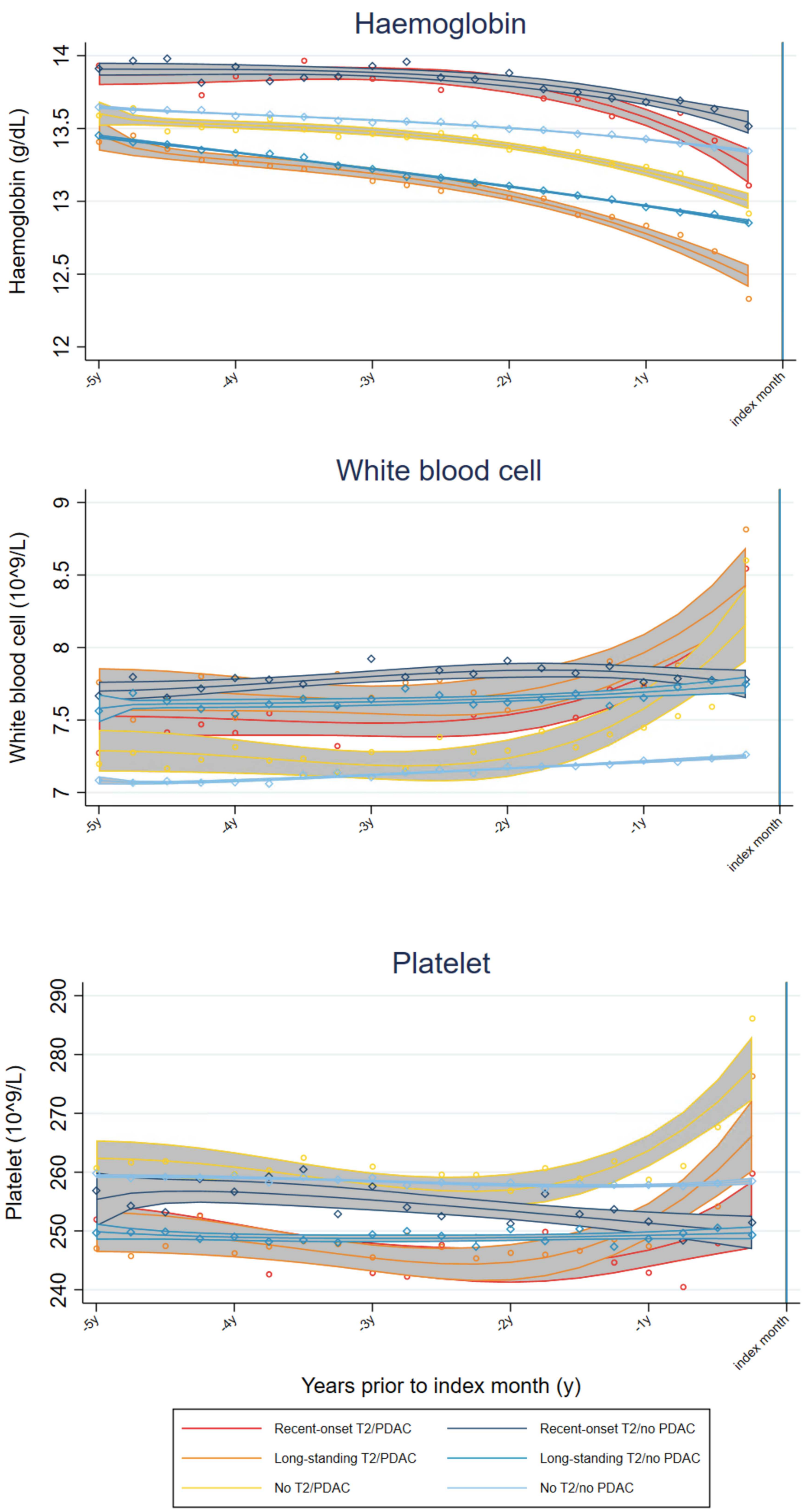
Haemoglobin
Haemoglobin (g/dL)
14
13.5
13
12.5
12
-5y
-4y
-3y
-2y
-1y
index month
White blood cell
White blood cell (10^9/L)
9
8.5
8
7.5
7
Platelet
Platelet (10^9/L)
290
280
270
260
250
240
Years prior to index month (y)
Recent-onset T2/PDAC
Recent-onset T2/no PDAC
Long-standing T2/PDAC
Long-standing T2/no PDAC
No T2/PDAC
No T2/no PDAC

Figure S6: Association of comorbidities with risk of PDAC by onset time; recent-onset defined as within 2 years prior to index date, long-standing defined as more than 2 years prior to index date.[a]

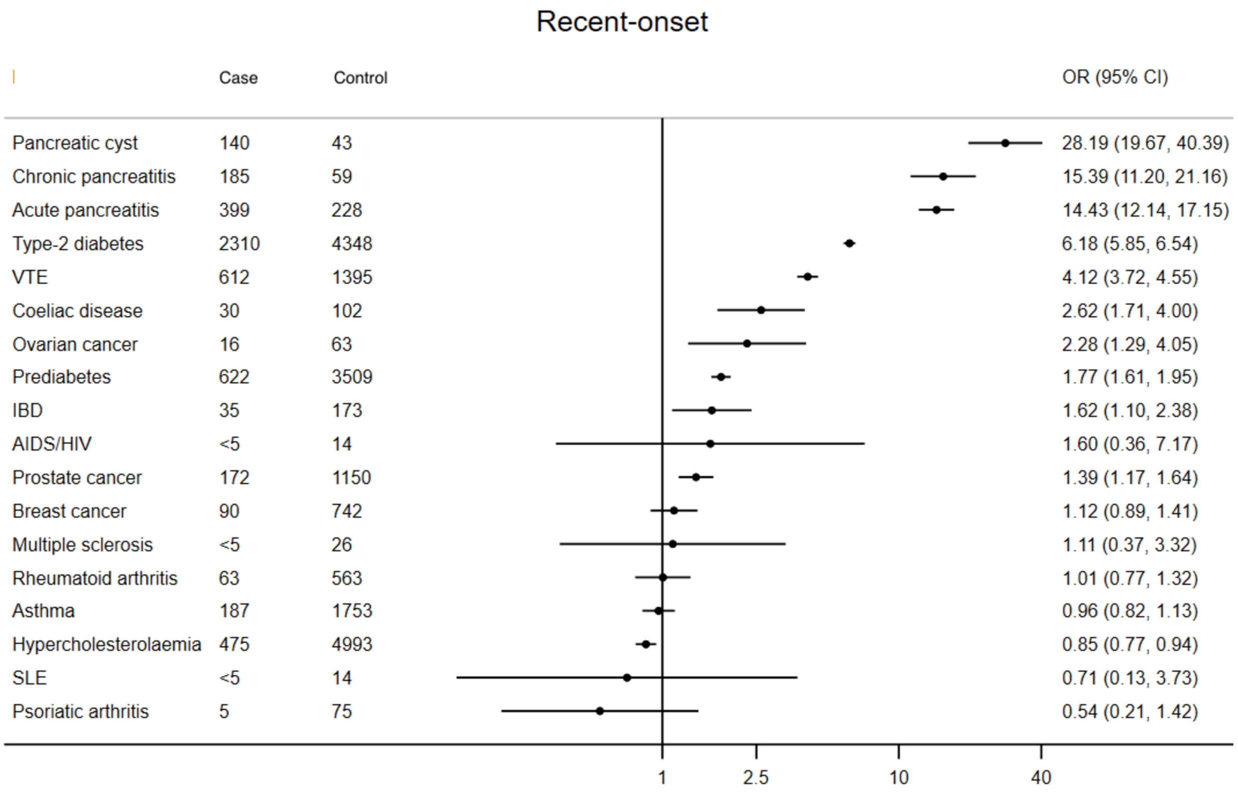

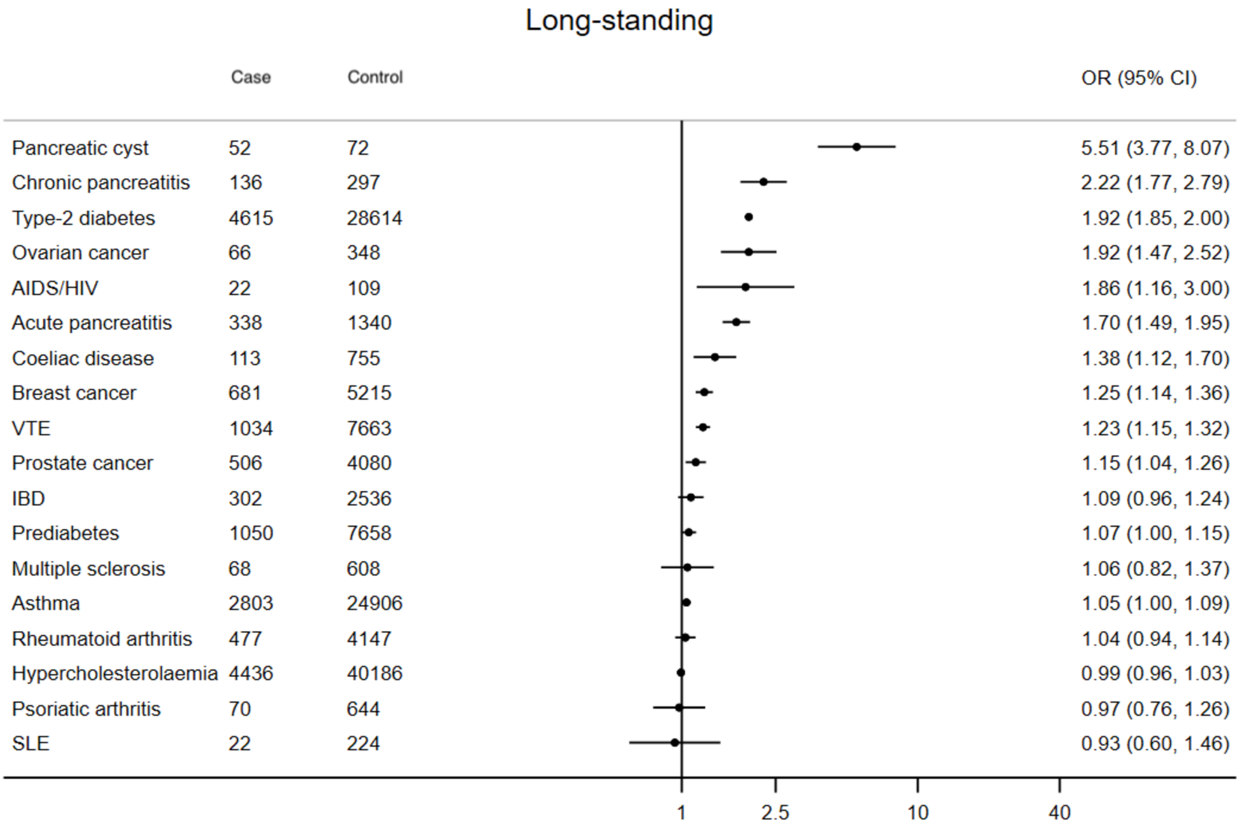

[a]Estimates are odds ratios adjusted for ethnic group, deprivation quintile, BMI, smoking and alcohol consumption. Reference group represent those with no corresponding comorbidities.

Figure S7: Association of medications with risk of PDAC by onset time; recent-onset defined as within 2 years prior to index date, long-standing defined as more than 2 years prior to index date.[a]

Recent-onset

	Case	Control	OR (95% CI)
Proton pump inhibitor	7052	20462	4.56 (4.40, 4.73)
Insulin	1018	1299	4.16 (3.76, 4.61)
Histamine-2 receptor blocker	1652	4498	2.74 (2.57, 2.93)
Sulphonylurea	1474	2446	2.64 (2.42, 2.88)
SGLT-2 inhibitor	143	422	1.76 (1.40, 2.20)
DPP-4 inhibitor	547	1521	1.66 (1.47, 1.87)
Metformin	1871	4466	1.61 (1.48, 1.75)
Alpha-glucosidase inhibitor	27	80	1.57 (0.95, 2.61)
Thiazolidinedione	257	724	1.51 (1.27, 1.79)
Meglitinide	32	92	1.23 (0.77, 1.98)
Digoxin	377	2460	0.97 (0.86, 1.09)
Aspirin	1743	13695	0.83 (0.78, 0.88)
Immunosuppresant	44	277	0.79 (0.55, 1.13)
GLP-1 agonist	44	236	0.78 (0.54, 1.13)
Bisphosphonate	701	5671	0.78 (0.72, 0.85)
Statin	2186	17848	0.75 (0.71, 0.80)

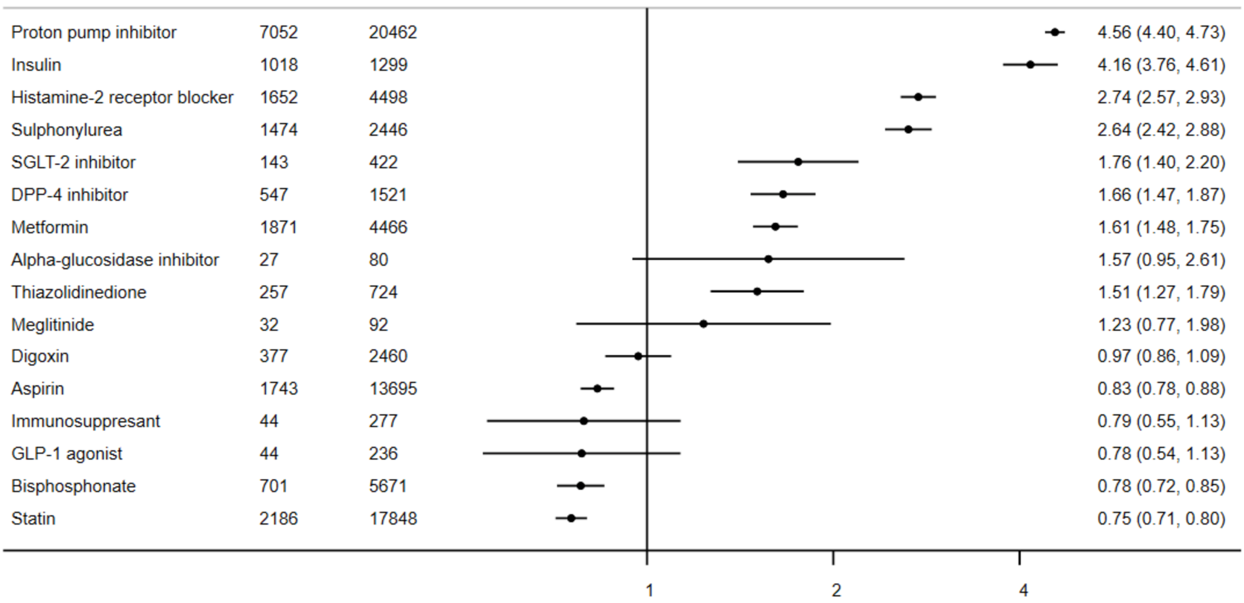

Long-standing

	Case	Control	OR (95% CI)
Insulin	966	4678	1.75 (1.60, 1.91)
Proton pump inhibitor	10315	89375	1.59 (1.53, 1.64)
Metformin	3272	19616	1.21 (1.11, 1.31)
Sulphonylurea	2331	13082	1.17 (1.09, 1.27)
Immunosuppresant	186	1320	1.17 (0.99, 1.39)
Meglitinide	89	432	1.10 (0.85, 1.41)
DPP4- inhibitor	481	2483	1.09 (0.97, 1.24)
Digoxin	1064	7855	1.09 (1.01, 1.17)
GLP-1 agonist	117	546	1.06 (0.84, 1.33)
Histamine-2 receptor blocker	4874	40676	1.04 (1.00, 1.08)
Aspirin	9242	75955	1.02 (0.99, 1.06)
SGLT-2 inhibitor	57	296	1.02 (0.74, 1.40)
Thiazolidinedione	695	3827	0.98 (0.88, 1.09)
Statin	9754	82316	0.96 (0.93, 1.00)
Bisphosphonate	1941	16774	0.95 (0.90, 1.00)
Alpha-glucosidase inhibitor	131	770	0.83 (0.68, 1.02)

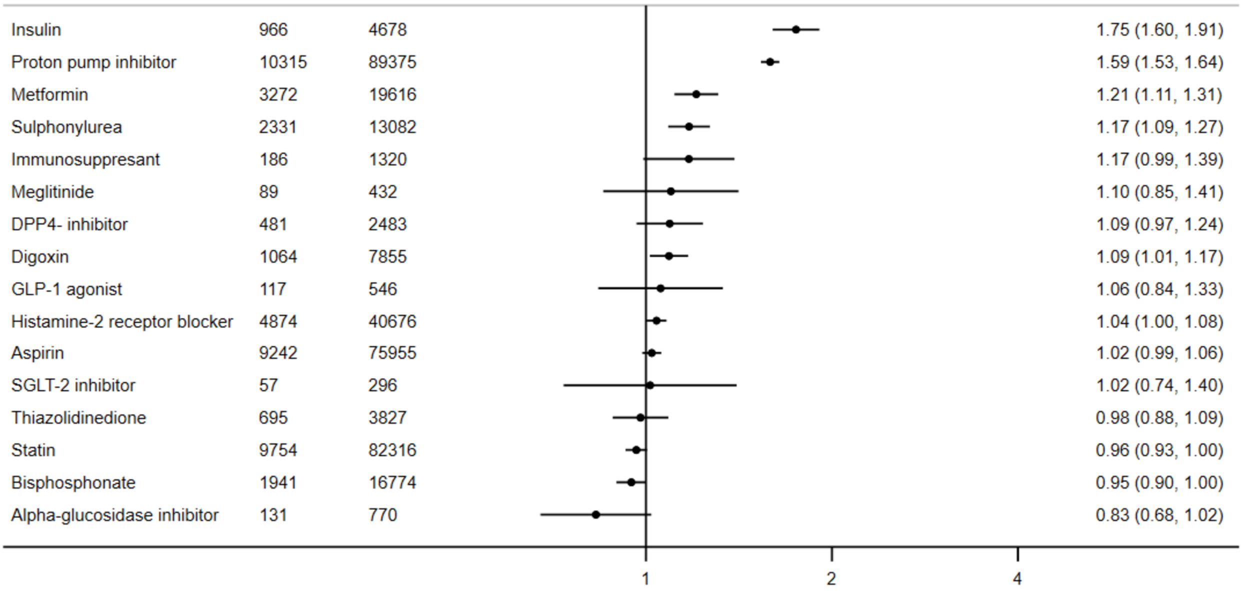

[a]Estimates are odds ratios adjusted for ethnic group, deprivation quintile, BMI, smoking, alcohol consumption and comorbidities. Reference group represent those with no corresponding medications.

Figure S8 (a): Subgroup analysis by age - association of comorbidities and risk of PDAC in age < 60 at index date

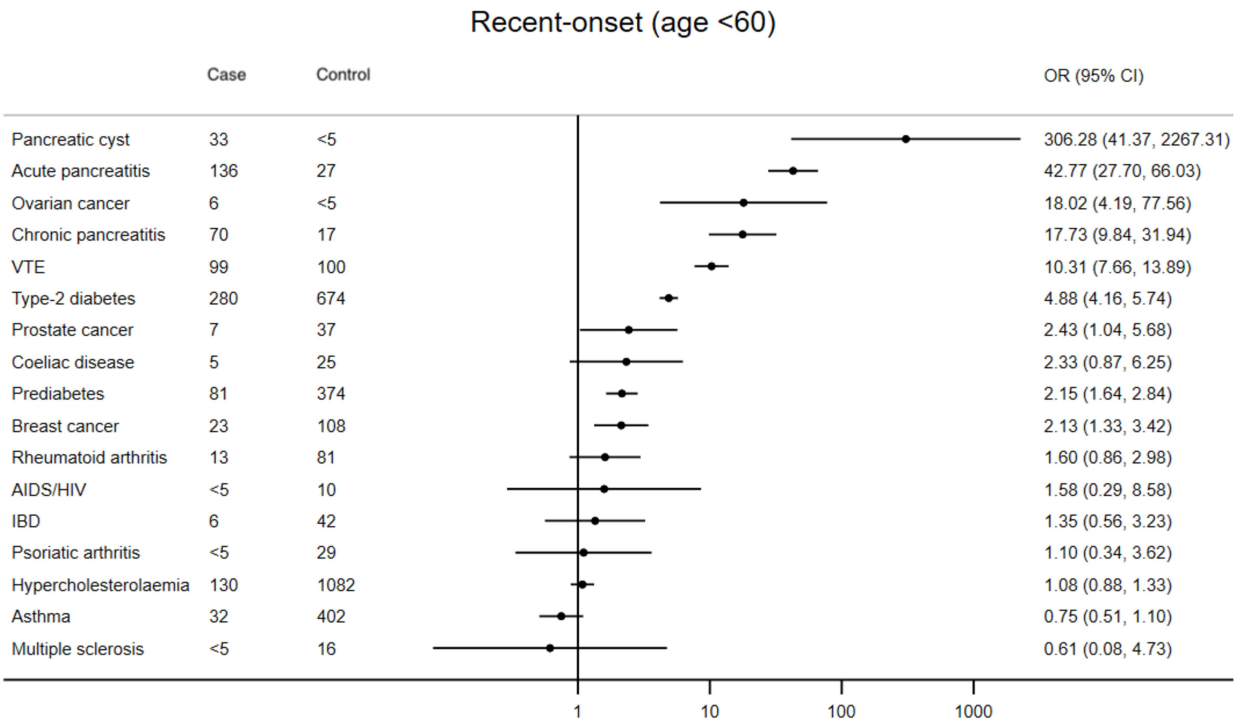

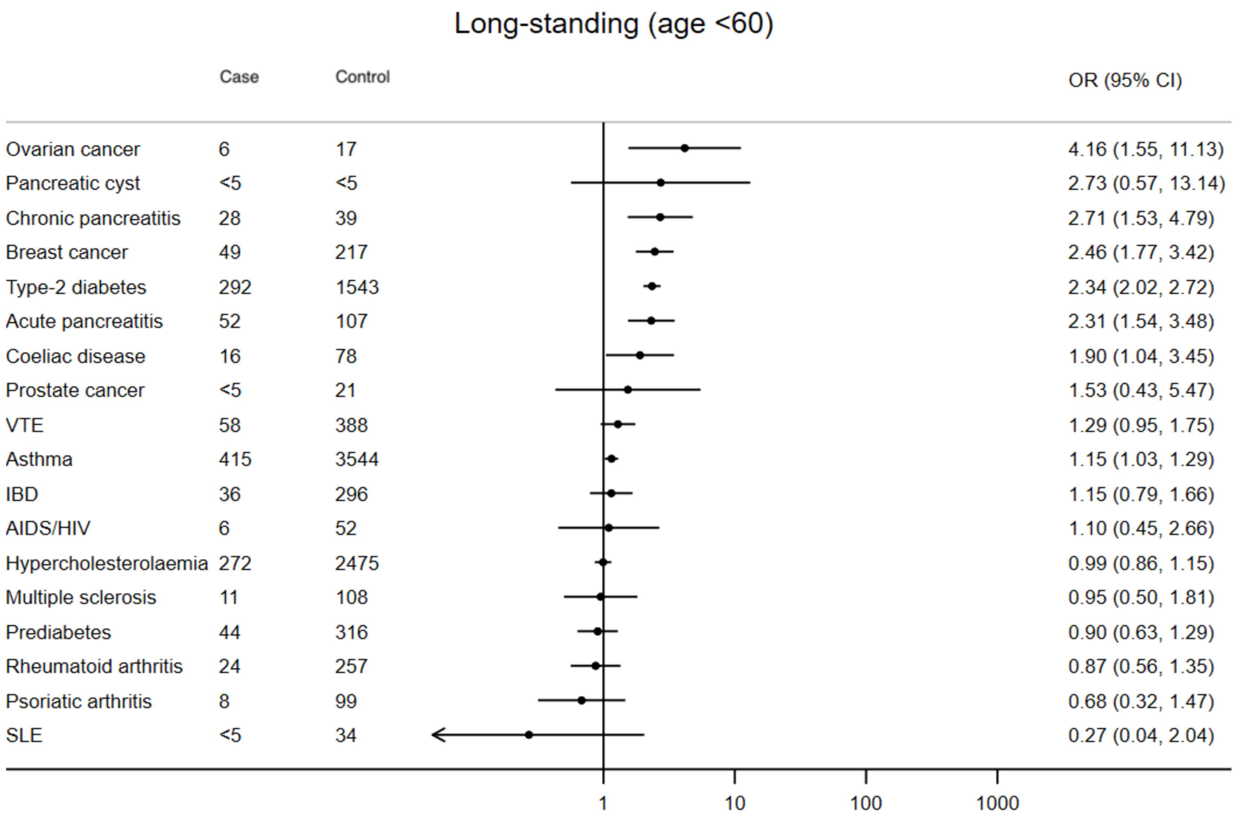

Tan PS, *et al. Gut* 2023; 72:512–521. doi: 10.1136/gutjnl-2021-326522

Figure S8 (b): Subgroup analysis by age - association of comorbidities and risk of PDAC in age >=60 at index date

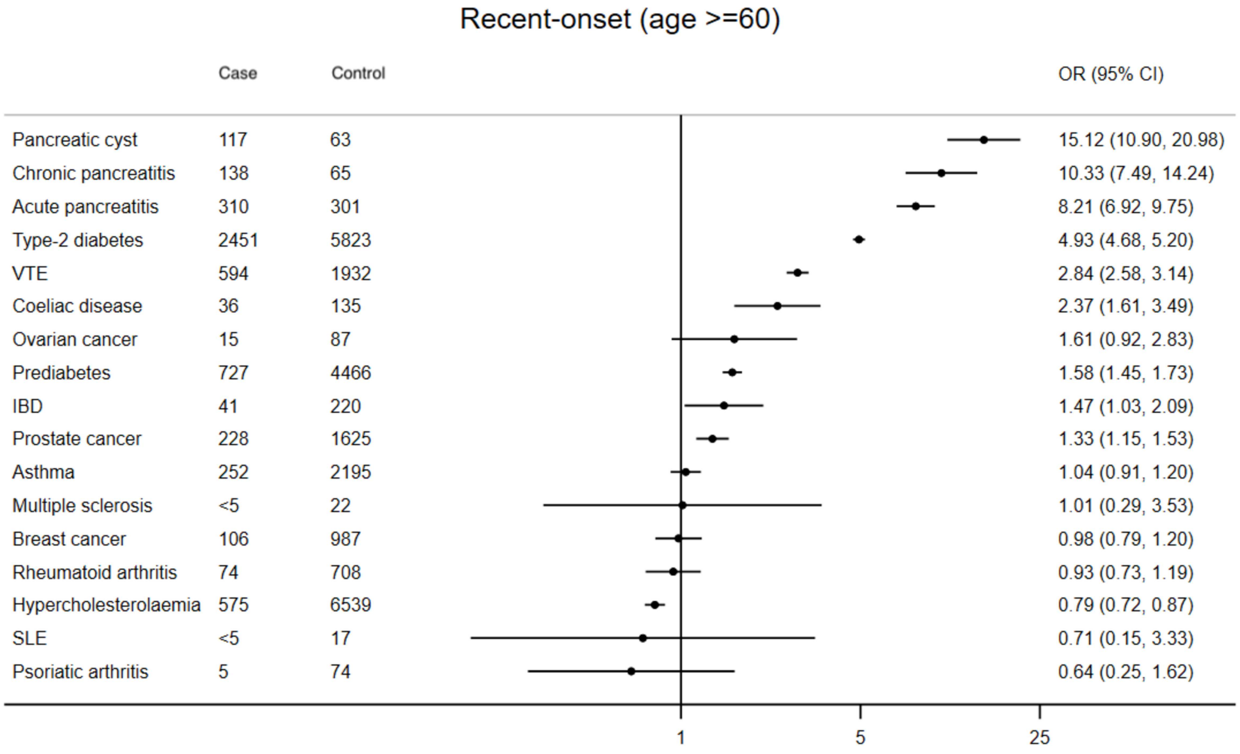

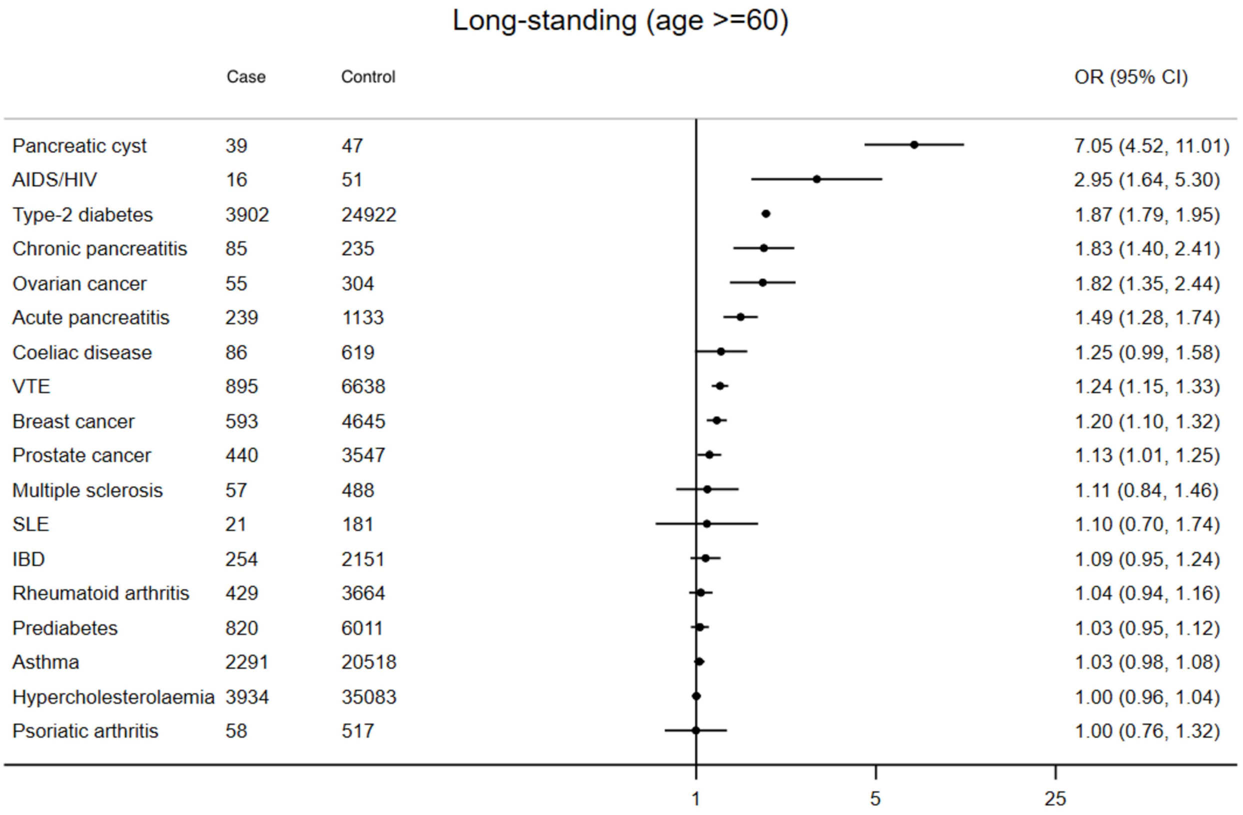

Tan PS, *et al. Gut* 2023; 72:512–521. doi: 10.1136/gutjnl-2021-326522

Figure S9 (a): Subgroup analysis by sex - association of comorbidities and risk of PDAC in female

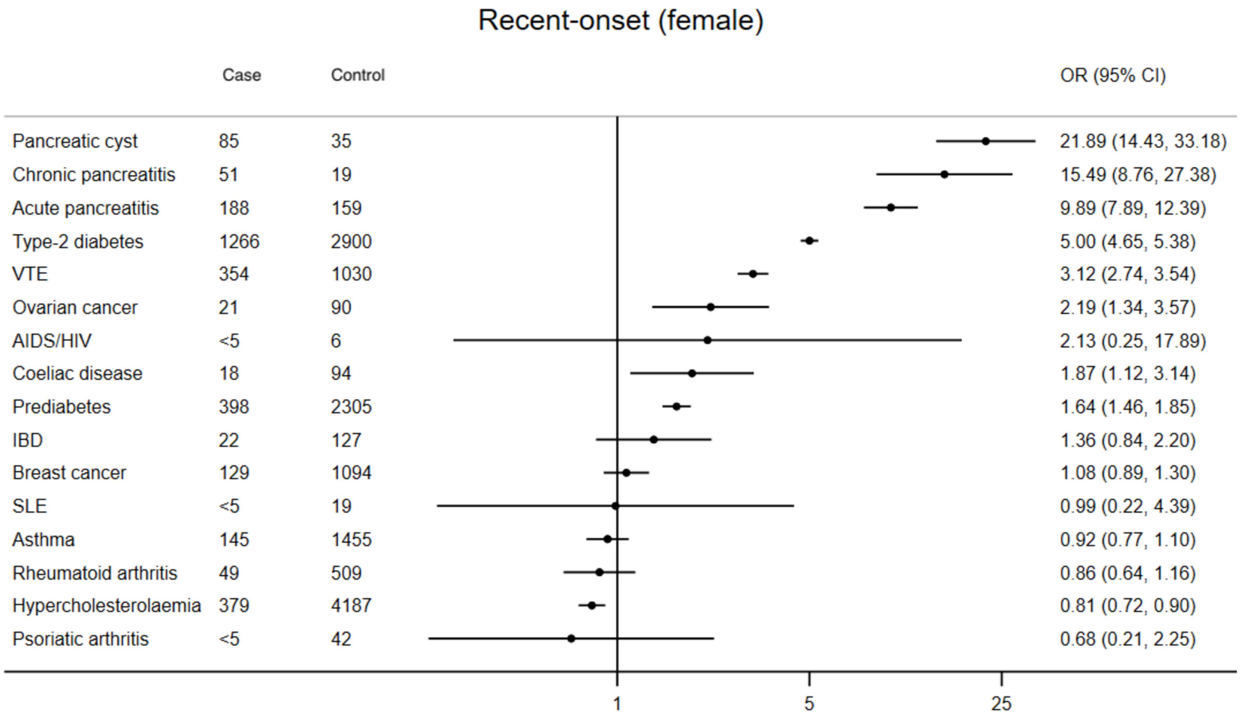

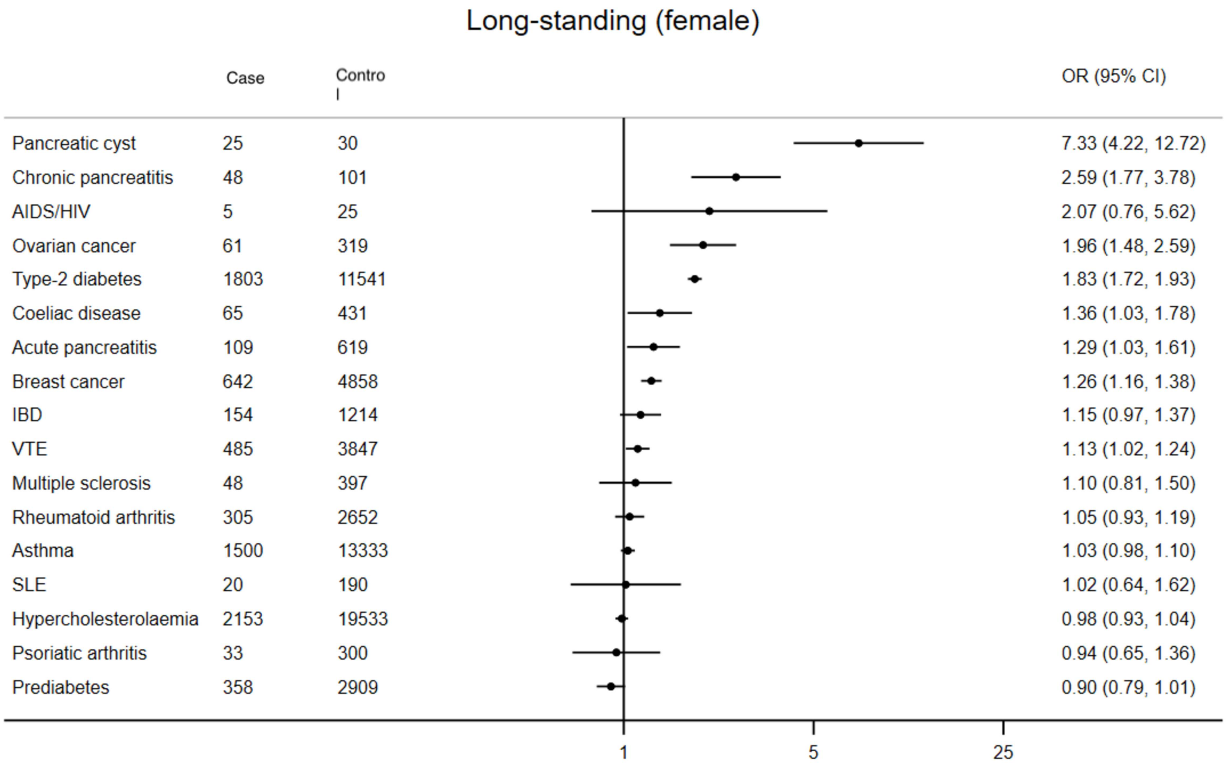

Figure S9 (b): Subgroup analysis by sex - association of comorbidities and risk of PDAC in male

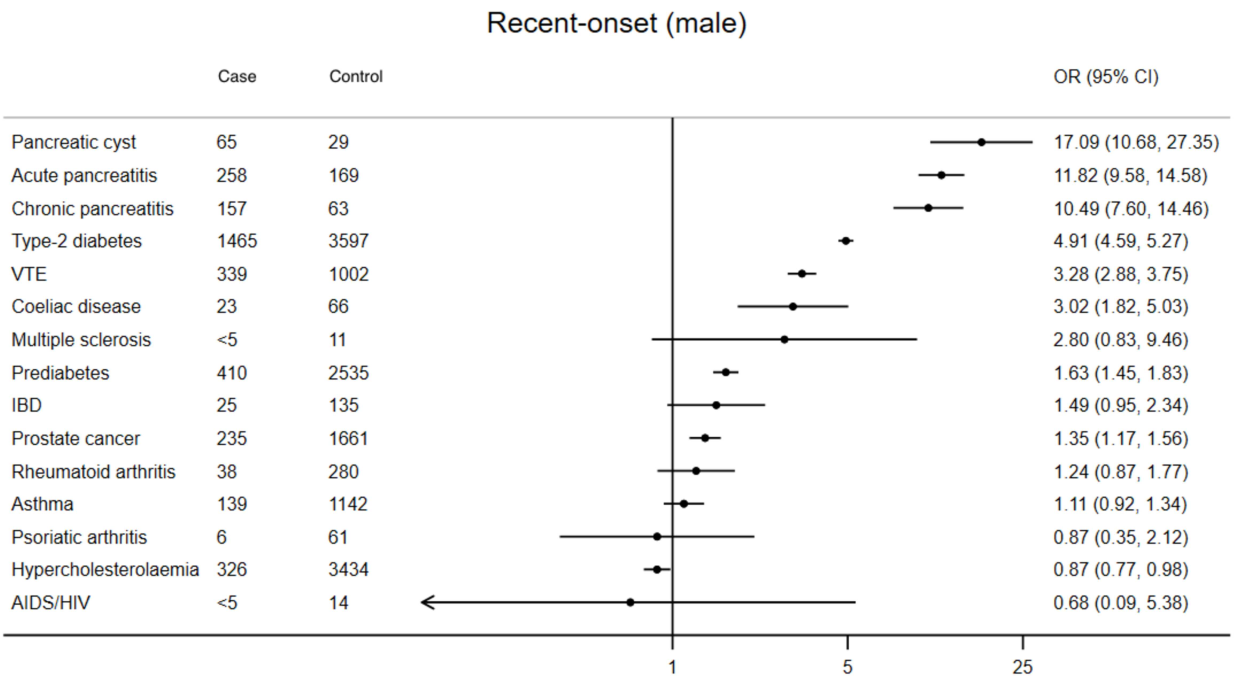

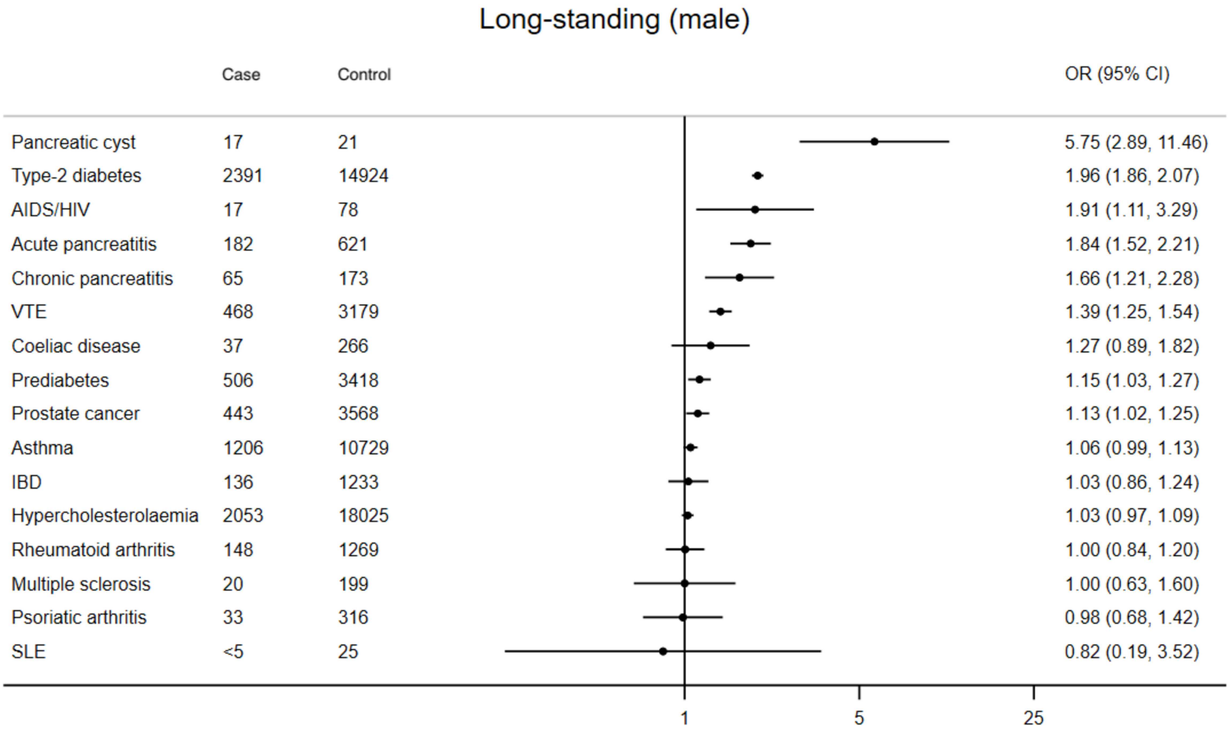

Figure S10 (a): Subgroup analysis by year of diagnosis - association of comorbidities and risk of PDAC in diagnosis year 2000-2010 (or matching year in controls)

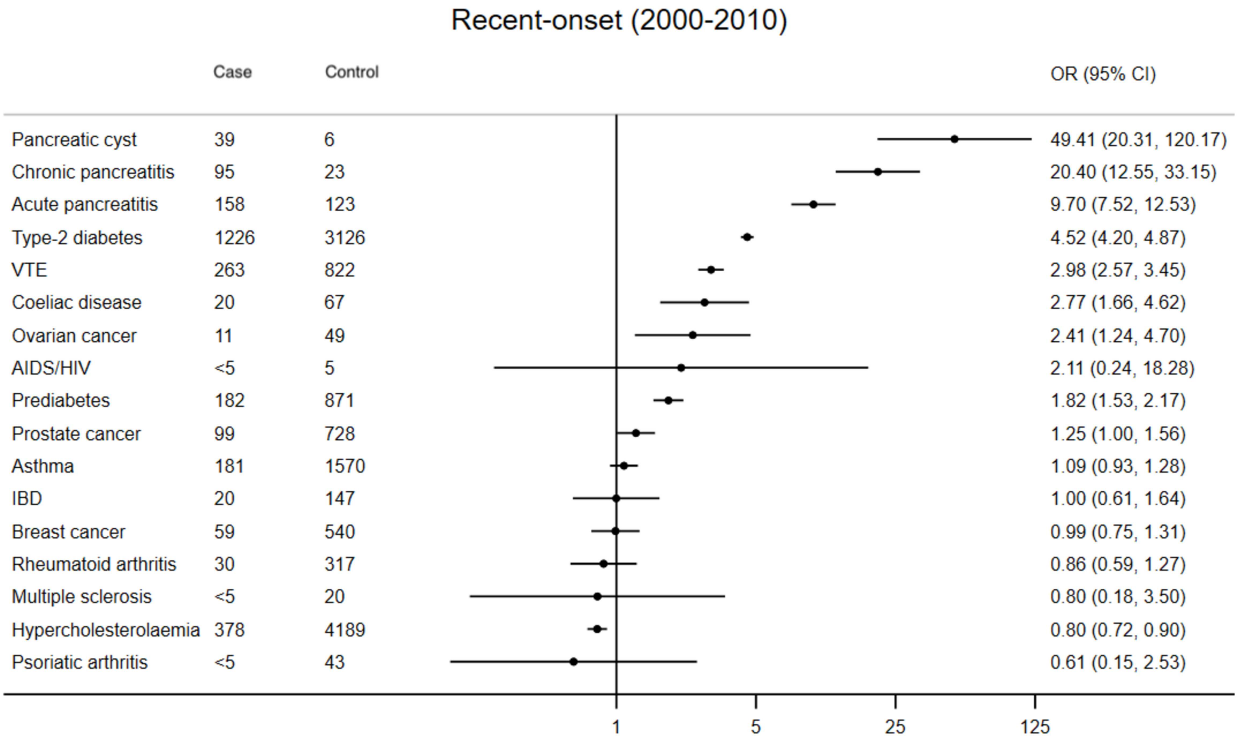

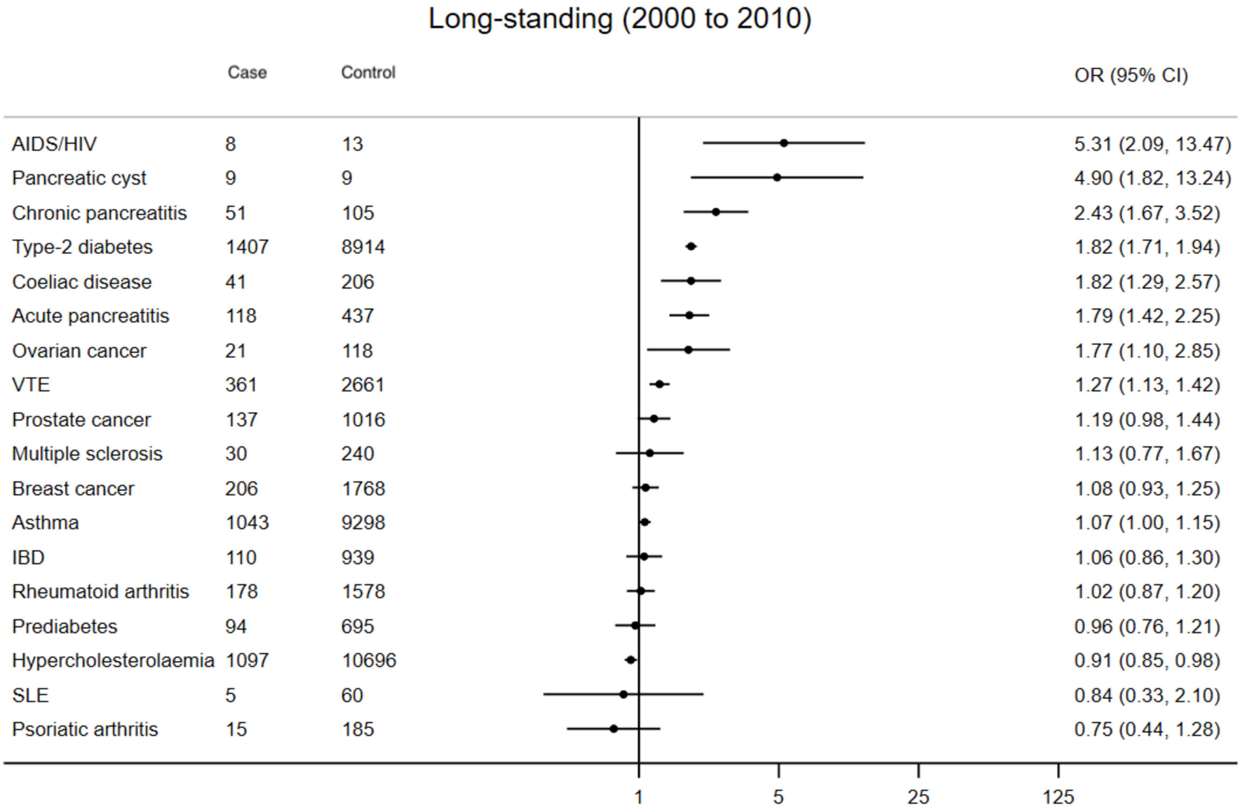

Figure S10 (b): Subgroup analysis by year of diagnosis- association of comorbidities and risk of PDAC in diagnosis year 2011-2020 (or matching year in controls)

Recent-onset (2011-2020)

	Case	Control	OR (95% CI)
Pancreatic cyst	111	58	16.60 (11.83, 23.29)
Acute pancreatitis	288	205	11.79 (9.72, 14.29)
Chronic pancreatitis	113	59	8.49 (6.00, 12.03)
Type-2 diabetes	1505	3371	5.34 (4.98, 5.72)
VTE	430	1210	3.34 (2.97, 3.75)
IBD	27	115	2.06 (1.33, 3.20)
Coeliac disease	21	93	2.02 (1.21, 3.35)
Ovarian cancer	10	41	1.93 (0.94, 3.95)
Prediabetes	626	3969	1.59 (1.44, 1.74)
Prostate cancer	136	934	1.43 (1.18, 1.72)
Breast cancer	70	555	1.20 (0.92, 1.55)
Rheumatoid arthritis	57	472	1.09 (0.82, 1.45)
SLE	<5	15	0.99 (0.21, 4.63)
Asthma	103	1027	0.89 (0.72, 1.09)
Psoriatic arthritis	7	60	0.88 (0.39, 2.02)
Hypercholesterolaemia	327	3432	0.88 (0.78, 0.99)
Multiple sclerosis	<5	18	0.86 (0.19, 3.94)
AIDS/HIV	<5	15	0.67 (0.09, 5.31)

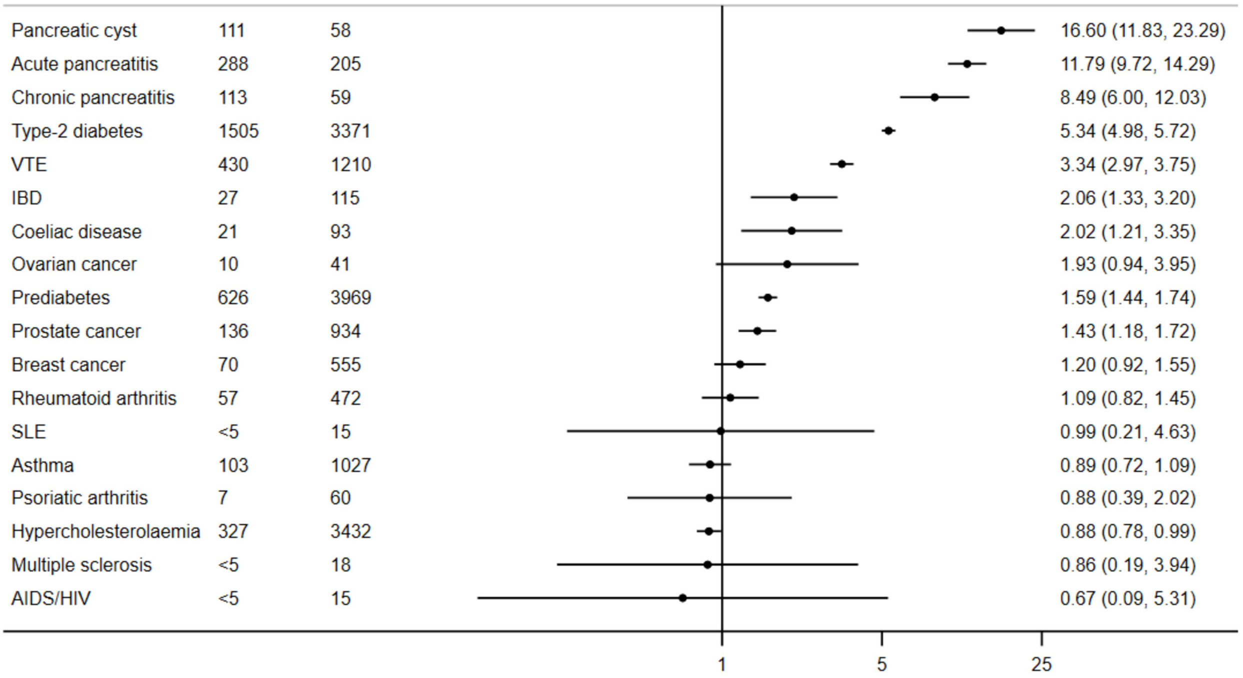

Long-standing (2011-2010)

	Case	Control	OR (95% CI)
Pancreatic cyst	33	42	7.19 (4.47, 11.59)
Ovarian cancer	40	203	2.05 (1.45, 2.91)
Type-2 diabetes	2787	17551	1.95 (1.85, 2.05)
Chronic pancreatitis	62	169	1.77 (1.28, 2.45)
AIDS/HIV	14	90	1.47 (0.82, 2.63)
Acute pancreatitis	173	803	1.47 (1.22, 1.76)
Breast cancer	436	3094	1.37 (1.23, 1.52)
VTE	592	4365	1.23 (1.12, 1.35)
IBD	180	1508	1.11 (0.94, 1.30)
Prostate cancer	306	2552	1.11 (0.98, 1.26)
Coeliac disease	61	491	1.10 (0.83, 1.46)
Hypercholesterolaemia	3109	26862	1.05 (1.00, 1.10)
Psoriatic arthritis	51	431	1.04 (0.77, 1.41)
Rheumatoid arthritis	275	2343	1.04 (0.91, 1.19)
SLE	17	155	1.03 (0.62, 1.72)
Asthma	1663	14764	1.03 (0.97, 1.09)
Multiple sclerosis	38	356	1.02 (0.73, 1.44)
Prediabetes	770	5632	1.02 (0.94, 1.11)

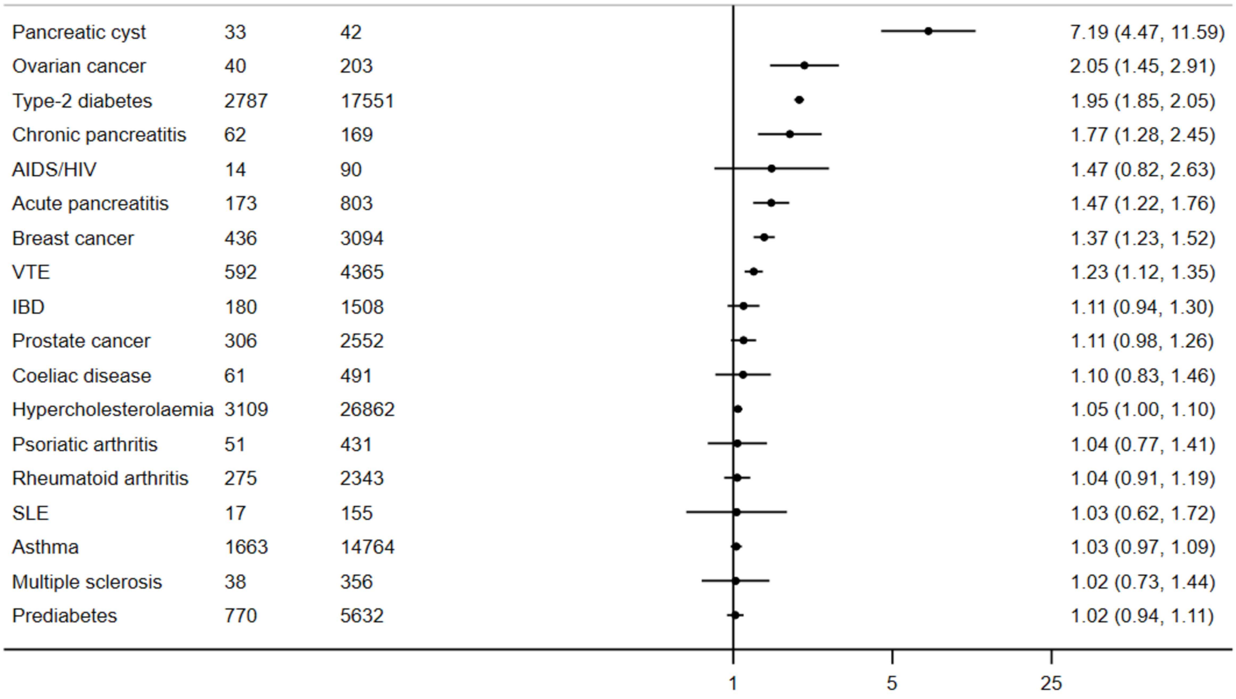

Tan PS, et al. Gut 2023; 72:512–521. doi: 10.1136/gutjnl-2021-326522

Figure S11 (a): Subgroup analysis by age - association of medications and risk of PDAC in age < 60 at index date

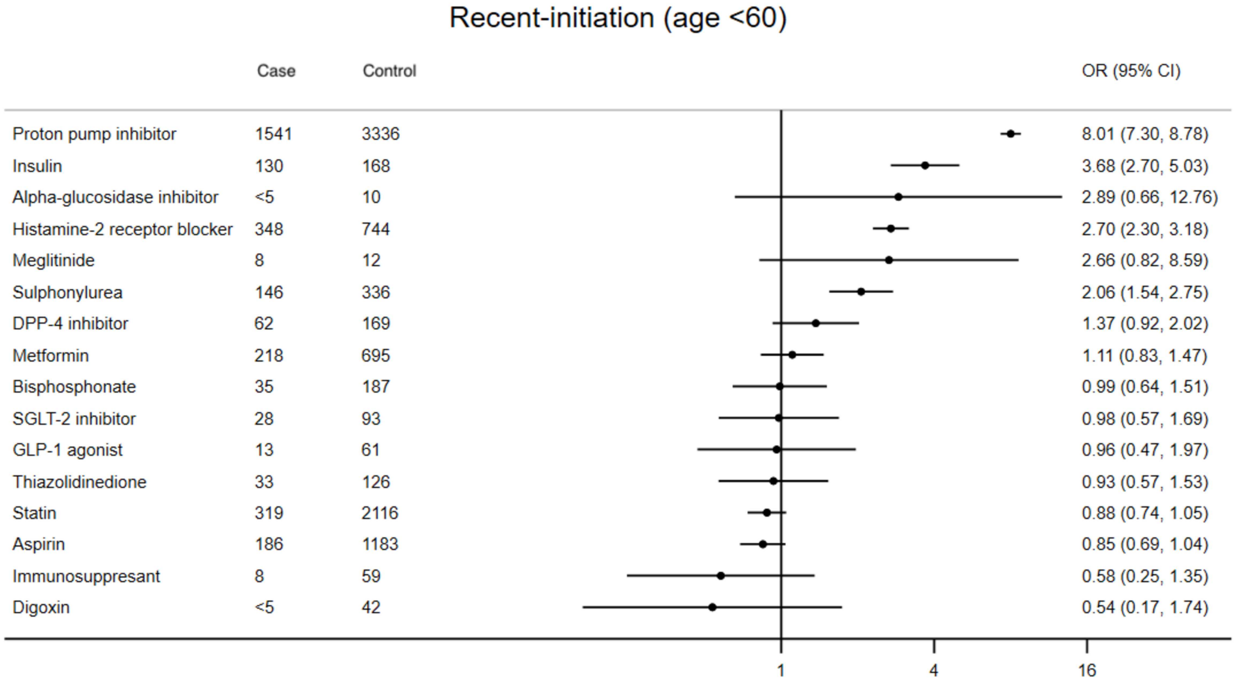

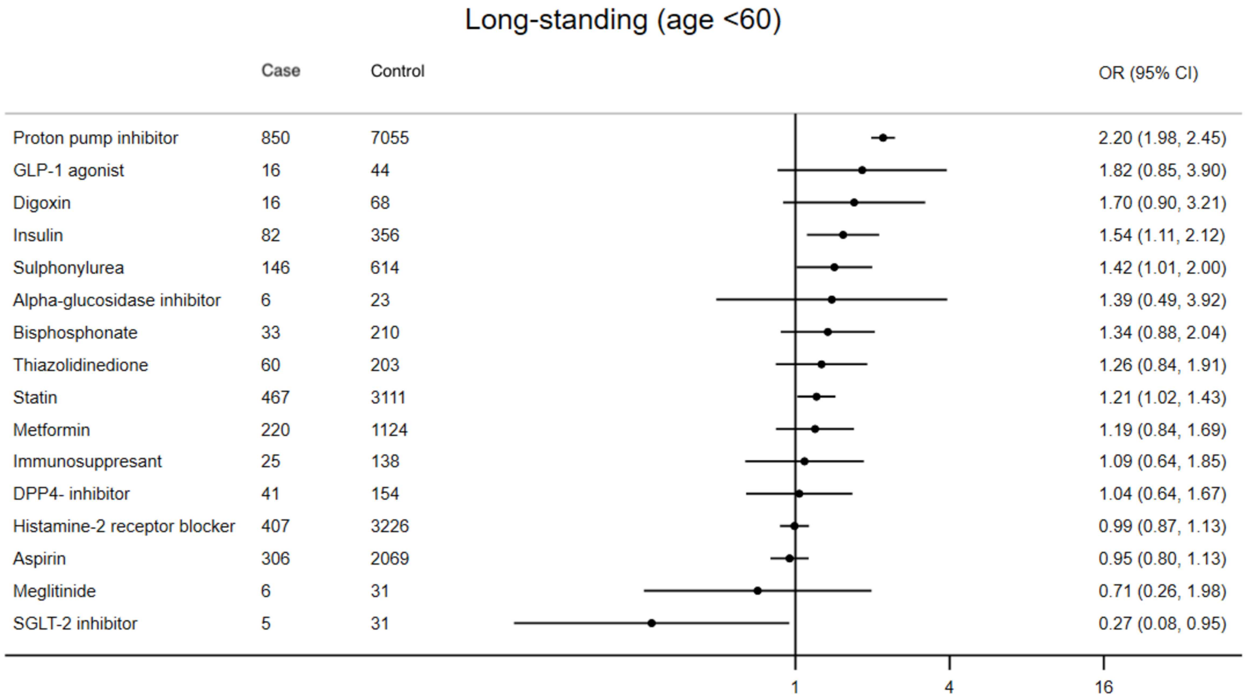

Tan PS, *et al. Gut* 2023; 72:512–521. doi: 10.1136/gutjnl-2021-326522

Figure S11 (b): Subgroup analysis by age - association of medications and risk of PDAC in age >= 60 at index date

Recent-initiation (age >=60)

	Case	Control	OR (95% CI)
Insulin	1050	1687	3.68 (3.35, 4.05)
Proton pump inhibitor	6574	26466	3.17 (3.05, 3.29)
Sulphonylurea	1579	3330	2.31 (2.13, 2.50)
Histamine-2 receptor blocker	1589	5905	2.09 (1.96, 2.23)
SGLT-2 inhibitor	133	451	1.61 (1.28, 2.01)
Metformin	2057	5883	1.54 (1.42, 1.66)
DPP-4 inhibitor	584	1941	1.46 (1.30, 1.63)
Thiazolidinedione	288	997	1.30 (1.11, 1.52)
Immunosuppresant	59	341	1.05 (0.78, 1.42)
Digoxin	505	3424	1.00 (0.91, 1.11)
Meglitinide	33	121	0.91 (0.59, 1.41)
Aspirin	2359	19298	0.88 (0.83, 0.93)
Alpha-glucosidase inhibitor	27	120	0.82 (0.51, 1.30)
GLP-1 agonist	54	273	0.80 (0.58, 1.11)
Statin	2844	24292	0.80 (0.76, 0.84)
Bisphosphonate	911	7877	0.79 (0.73, 0.85)

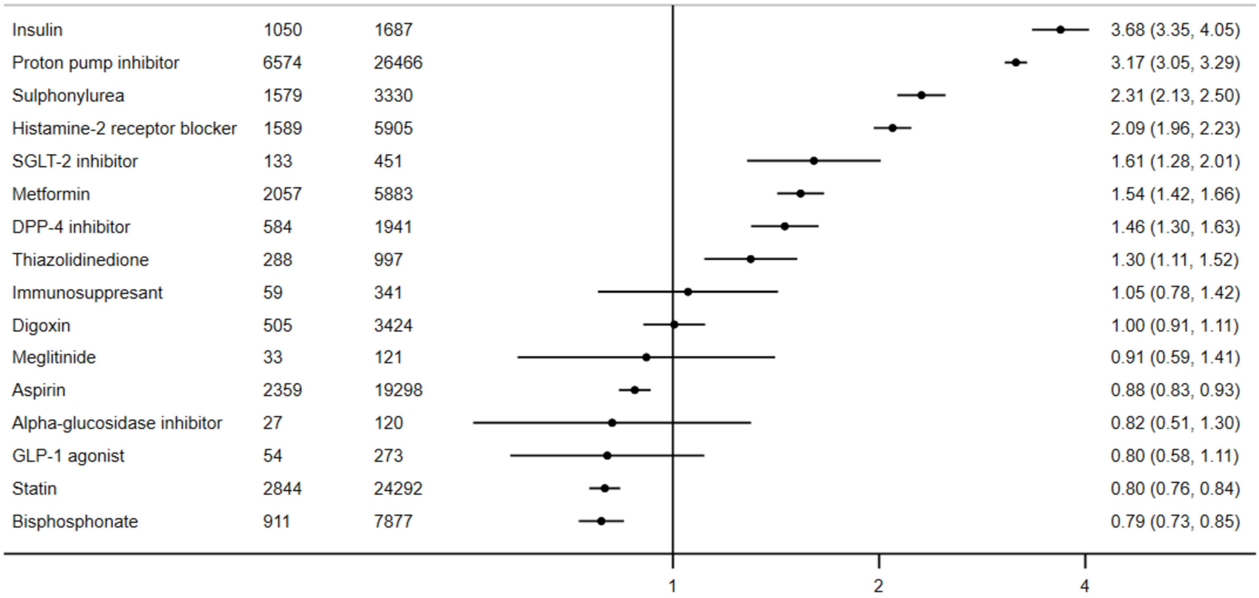

Long-standing (age >=60)

	Case	Control	OR (95% CI)
Insulin	722	3766	1.73 (1.57, 1.91)
Proton pump inhibitor	8402	72980	1.53 (1.47, 1.59)
SGLT-2 inhibitor	34	143	1.46 (0.97, 2.21)
Metformin	2648	16380	1.20 (1.10, 1.31)
Sulphonylurea	1934	11248	1.16 (1.06, 1.26)
Meglitinide	74	360	1.15 (0.88, 1.51)
DPP4- inhibitor	341	1740	1.15 (0.99, 1.32)
Immunosuppresant	138	1059	1.10 (0.91, 1.33)
Digoxin	916	6781	1.08 (1.00, 1.16)
Aspirin	8134	67100	1.03 (0.99, 1.07)
Histamine-2 receptor blocker	4182	35299	1.03 (0.99, 1.07)
Thiazolidinedione	571	3225	0.99 (0.88, 1.11)
GLP-1 agonist	78	404	0.96 (0.73, 1.26)
Bisphosphonate	1663	14171	0.96 (0.91, 1.02)
Statin	8310	70645	0.95 (0.91, 0.99)
Alpha-glucosidase inhibitor	121	697	0.89 (0.72, 1.10)

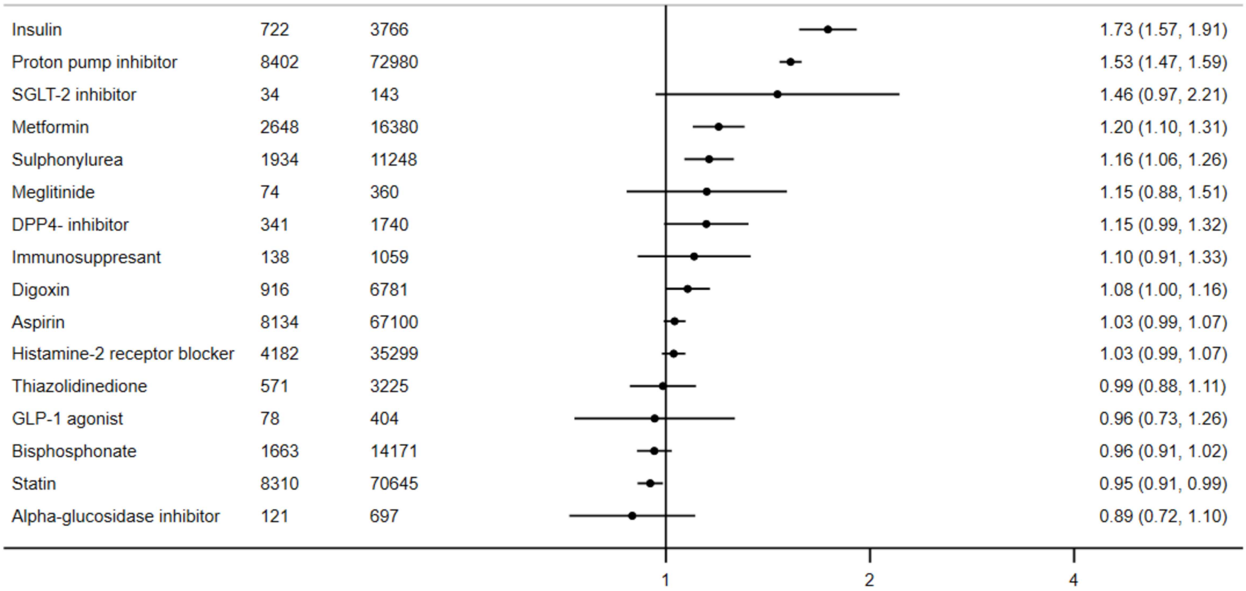

Tan PS, *et al. Gut* 2023; 72:512–521. doi: 10.1136/gutjnl-2021-326522

Figure S12 (a): Subgroup analysis by sex - association of medications and risk of PDAC in female

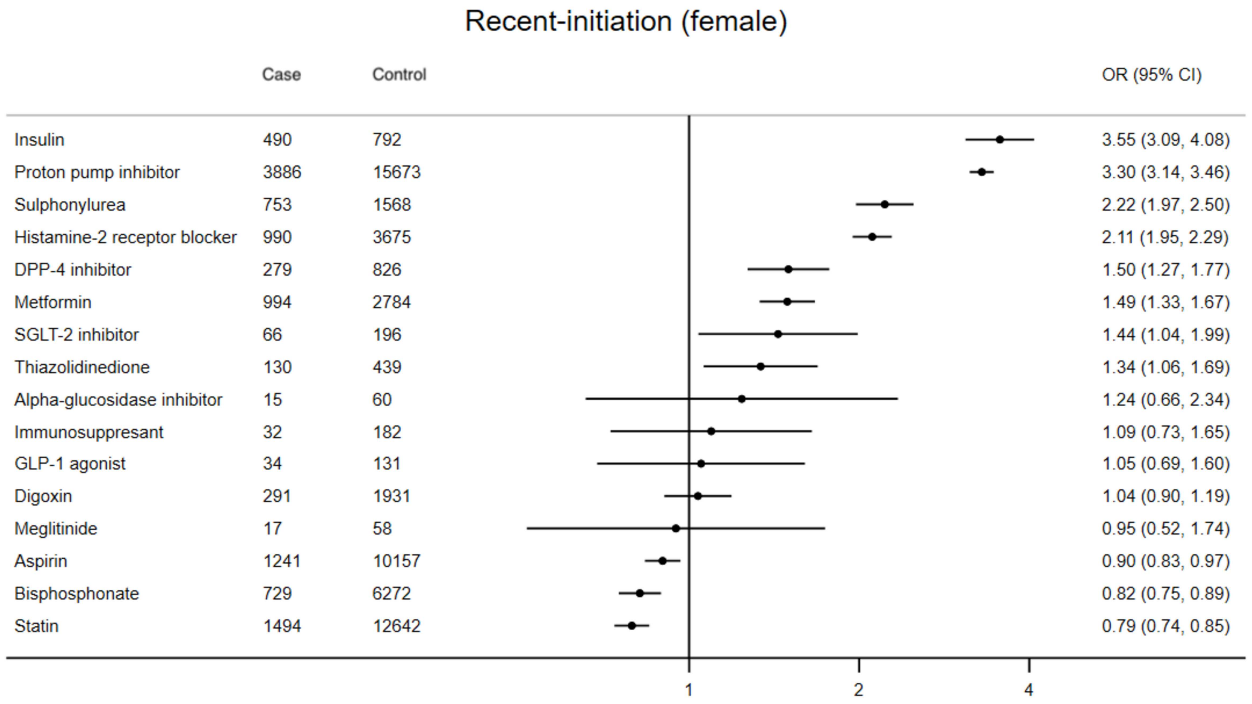

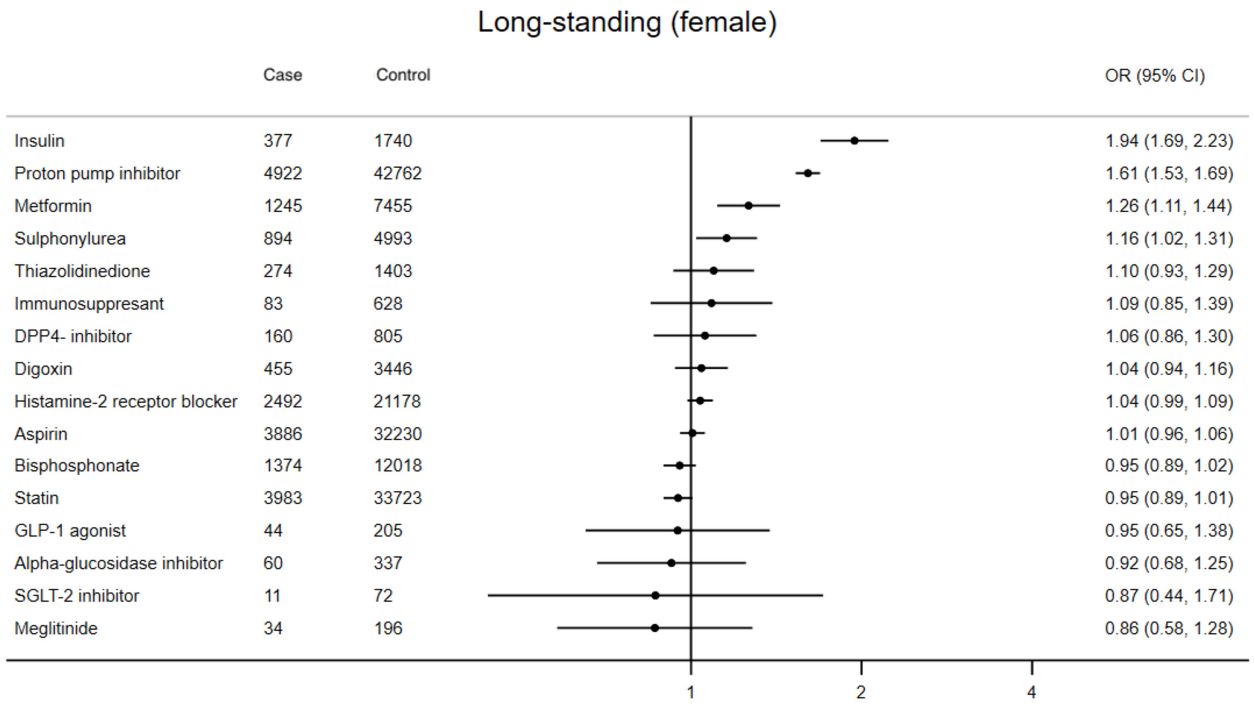

Figure S12 (b): Subgroup analysis by sex - association of medications and risk of PDAC in male

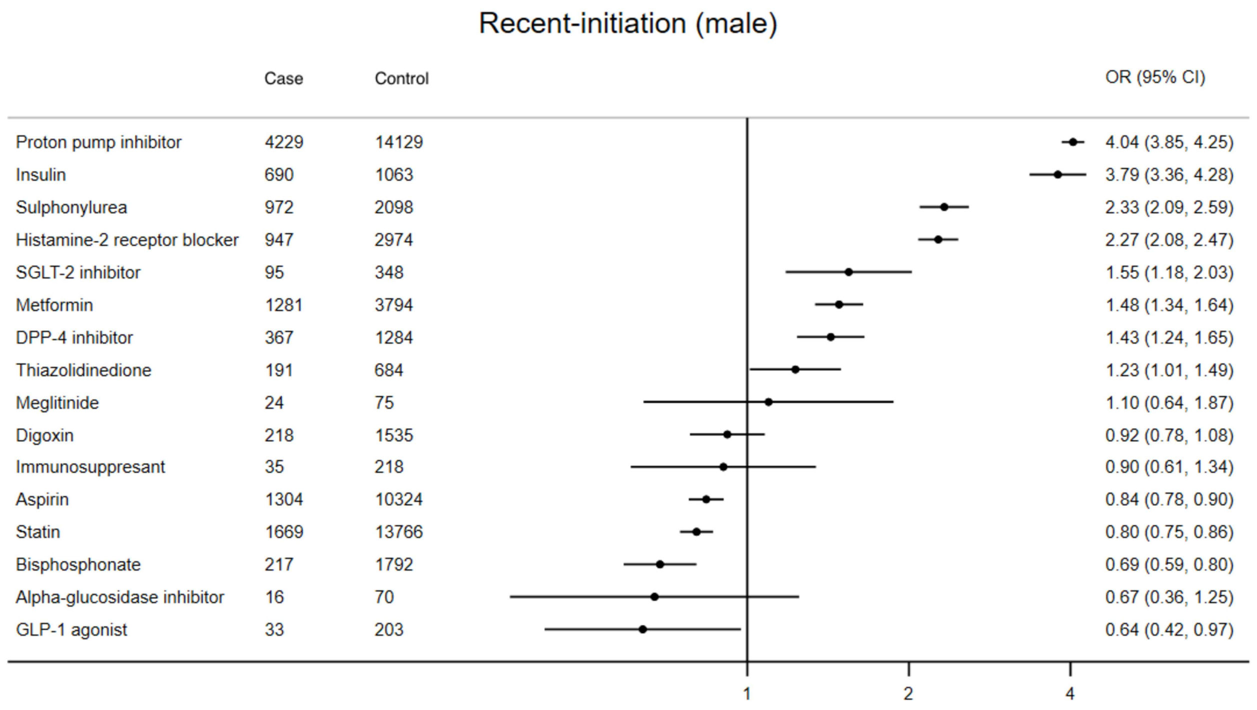

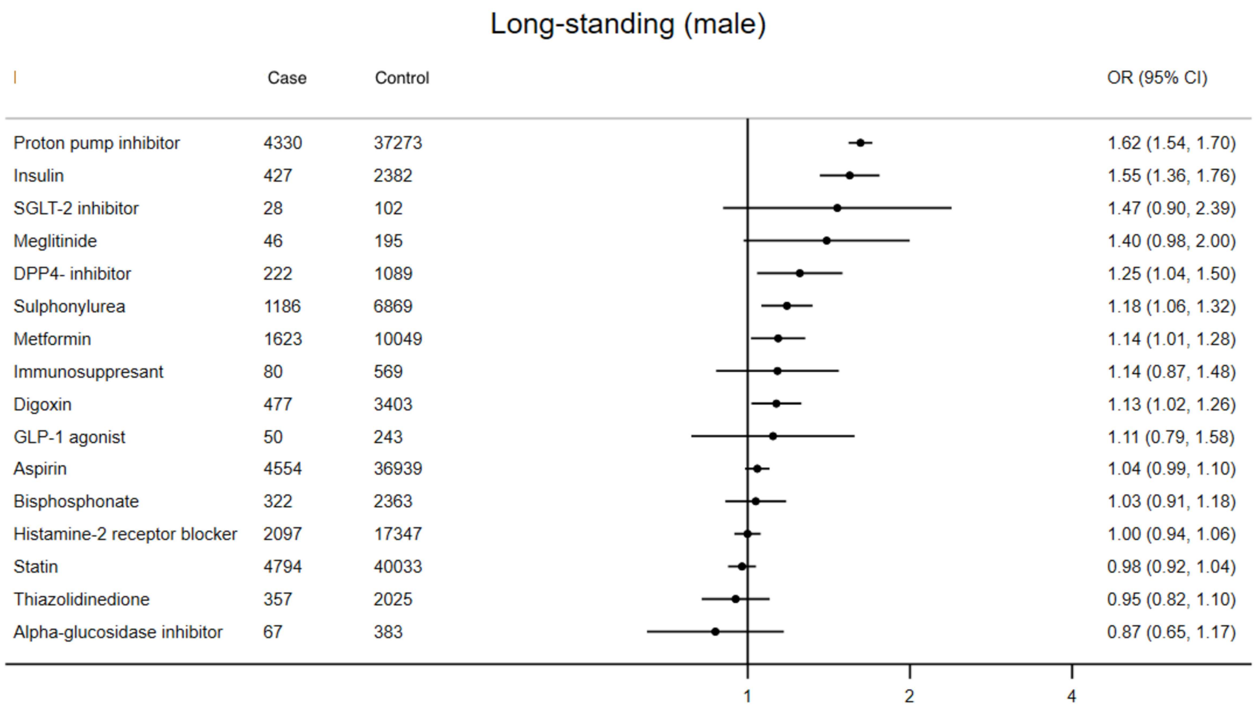

Figure S13 (a): Subgroup analysis year of diagnosis - association of medications and risk of PDAC in diagnosis year 2000-2010 (or matching year in controls)

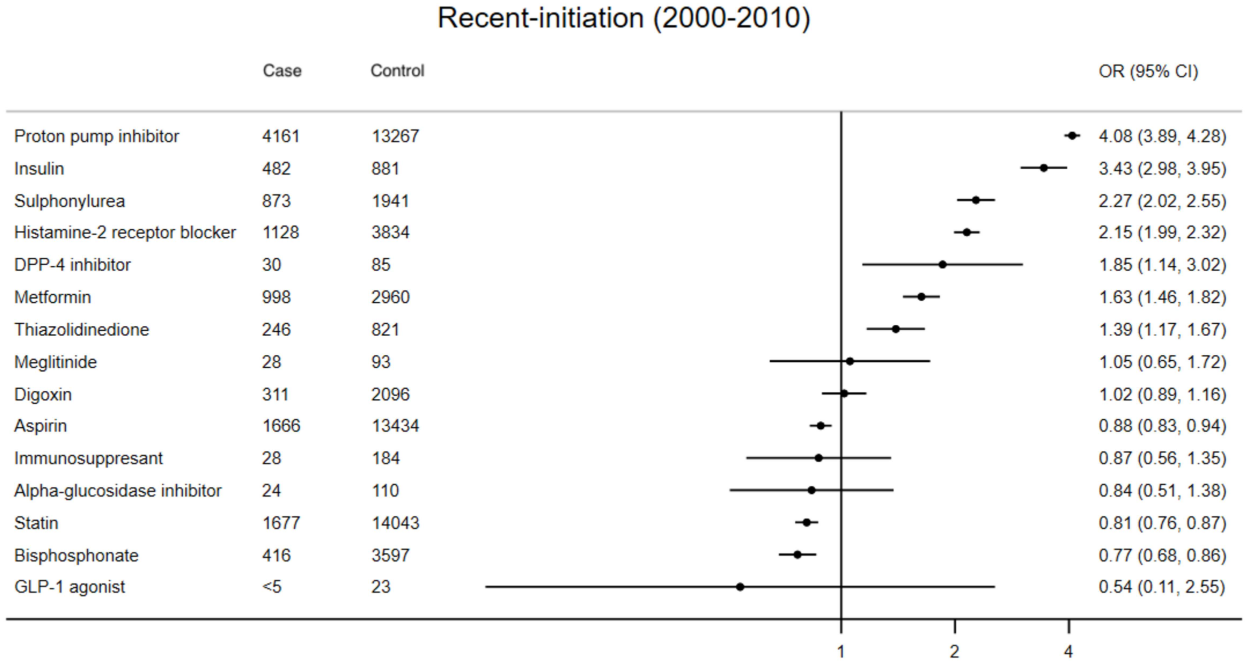

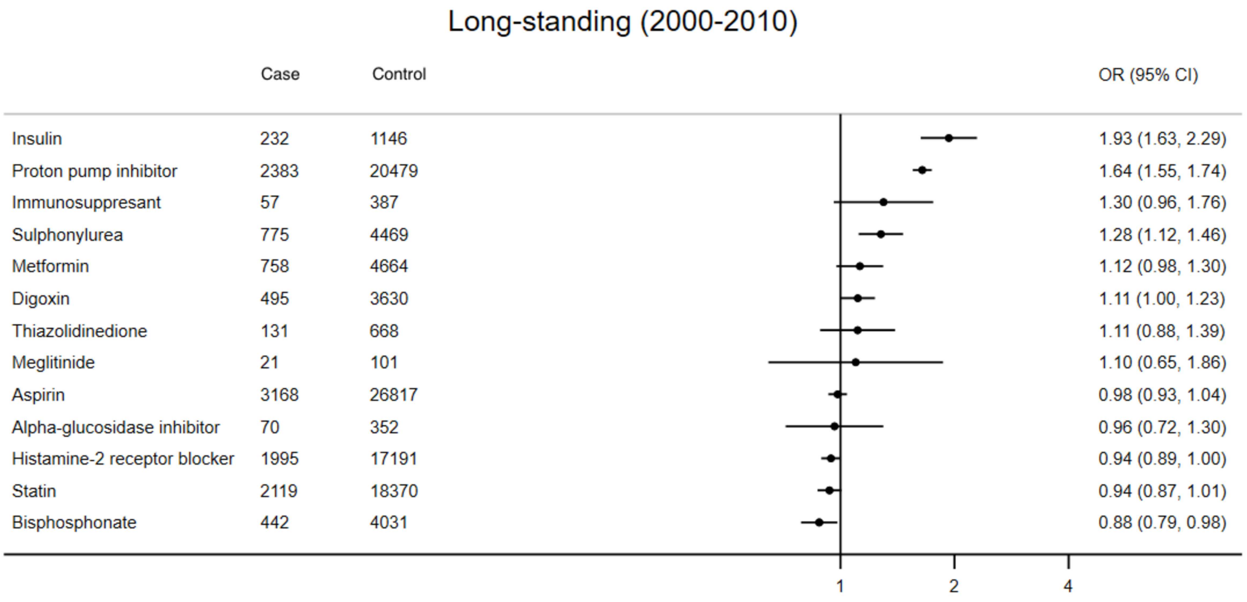

Figure S13 (b): Subgroup analysis year of diagnosis - association of medications and risk of PDAC in diagnosis year 2011-2020 (or matching year in controls)

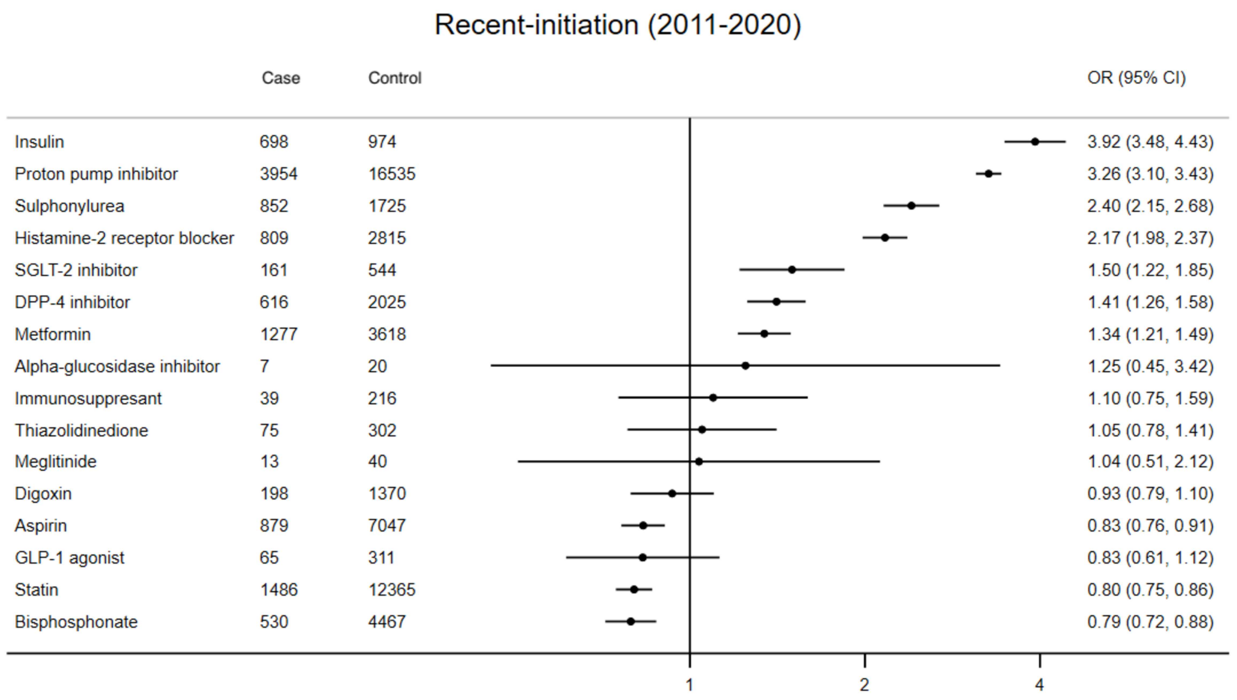

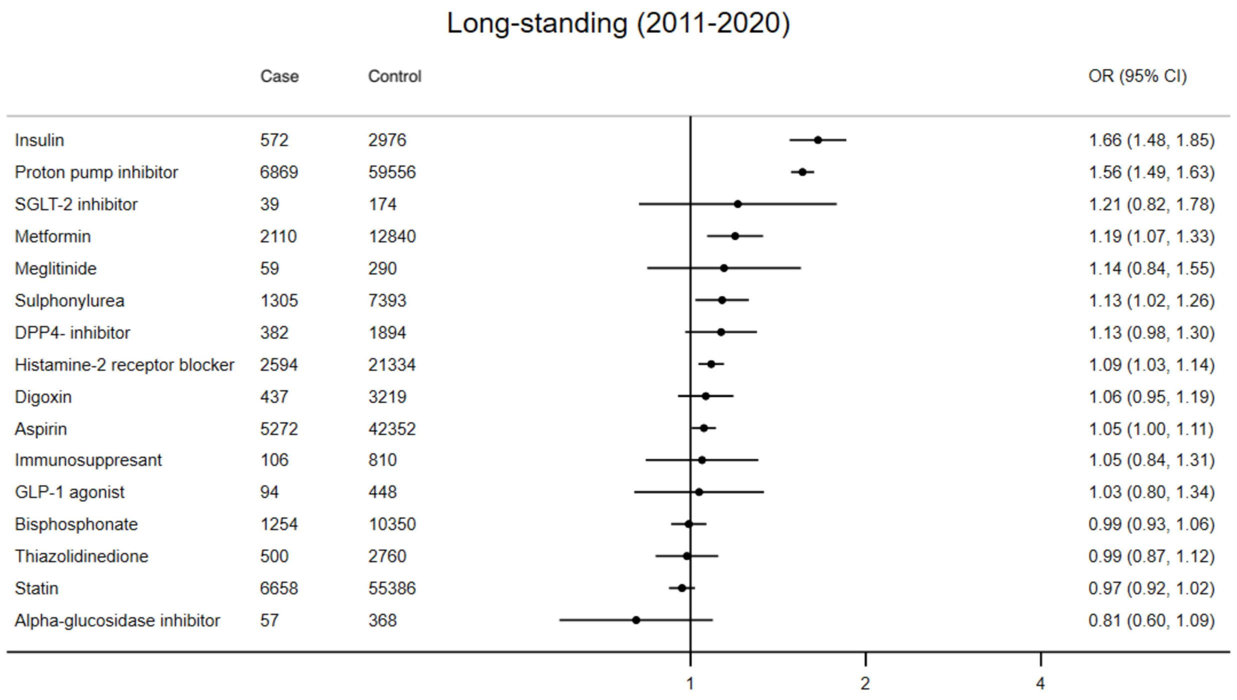

Tan PS, *et al. Gut* 2023; 72:512–521. doi: 10.1136/gutjnl-2021-326522

Figure S14 (a): Subgroup analysis by body mass index (BMI) - association of comorbidities and risk of PDAC in individuals with BMI <25kg/m^{2} *

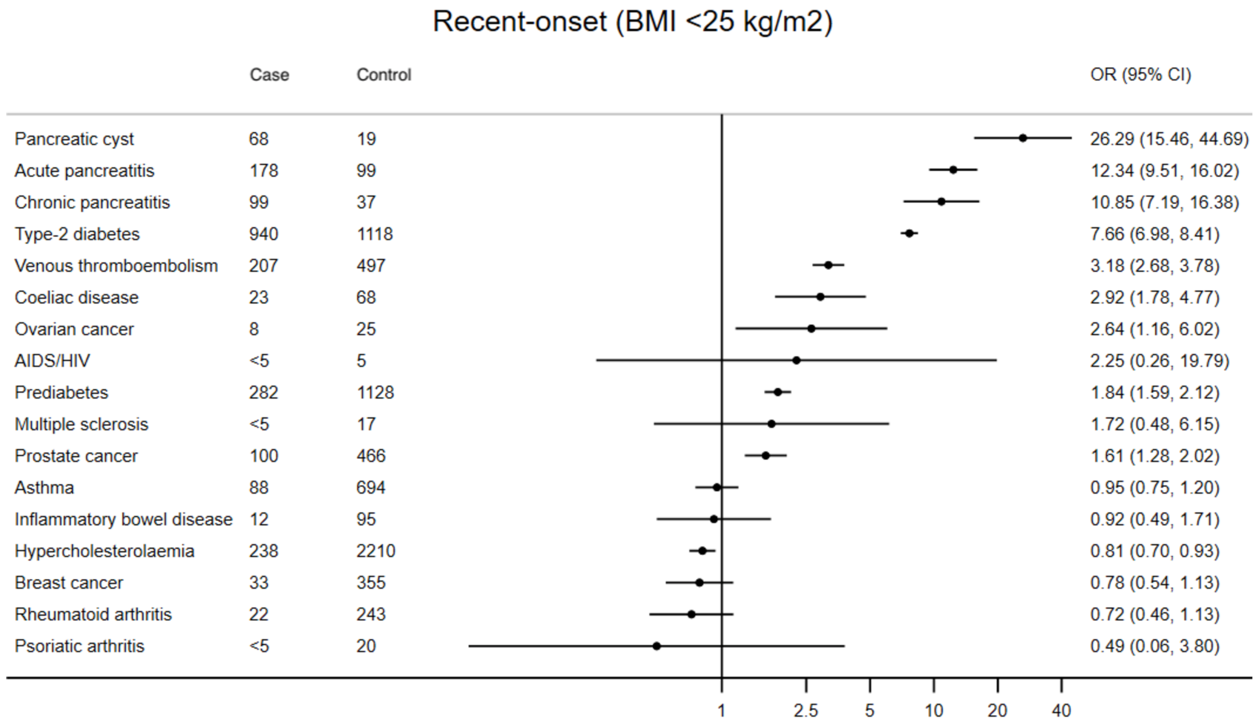

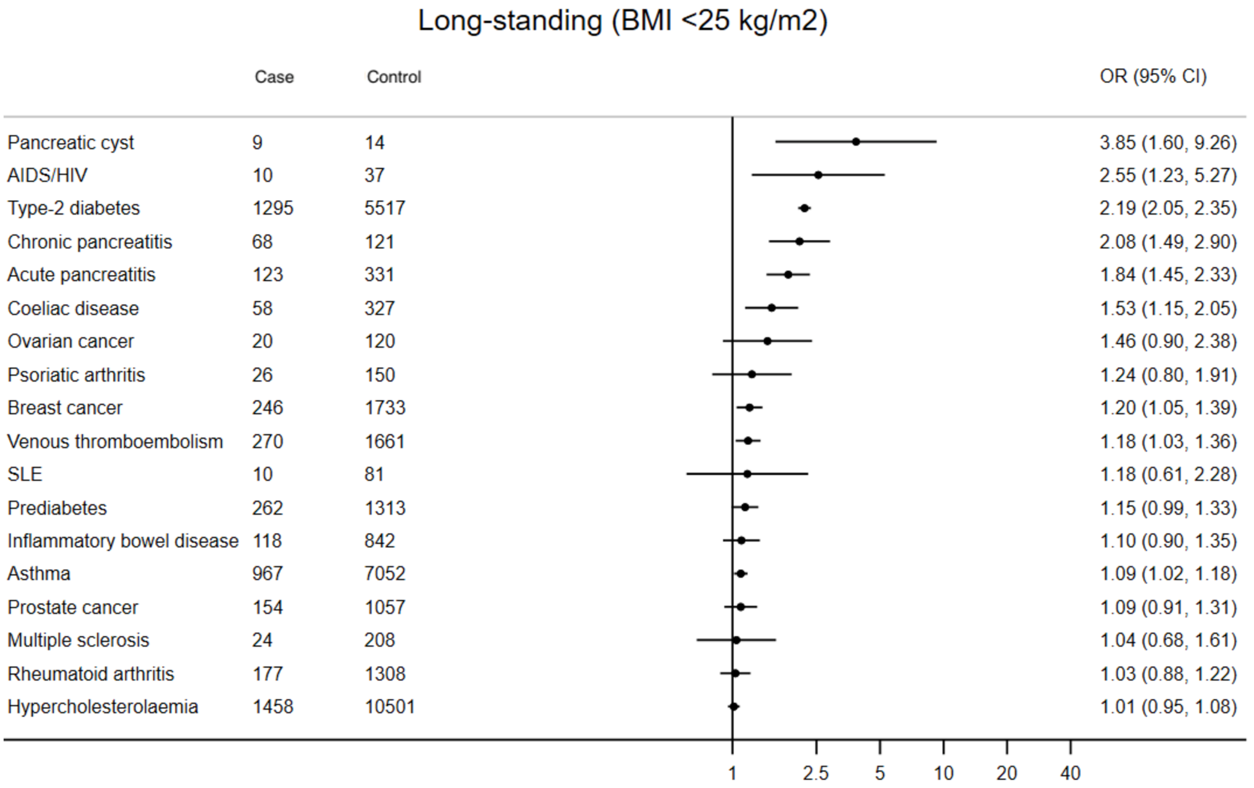

* Subgrouping variables were not matching factors in the case-control study design, hence, these subgroup analysis were performed without matching but instead analysed using mixed effects logistic regression using practice as a random-effect variable and additionally adjusted for other matching factors (age, sex, year of diagnosis).

Figure S14 (b): Subgroup analysis by body mass index (BMI) - association of comorbidities and risk of PDAC in individuals with BMI >=25kg/m^{2*}

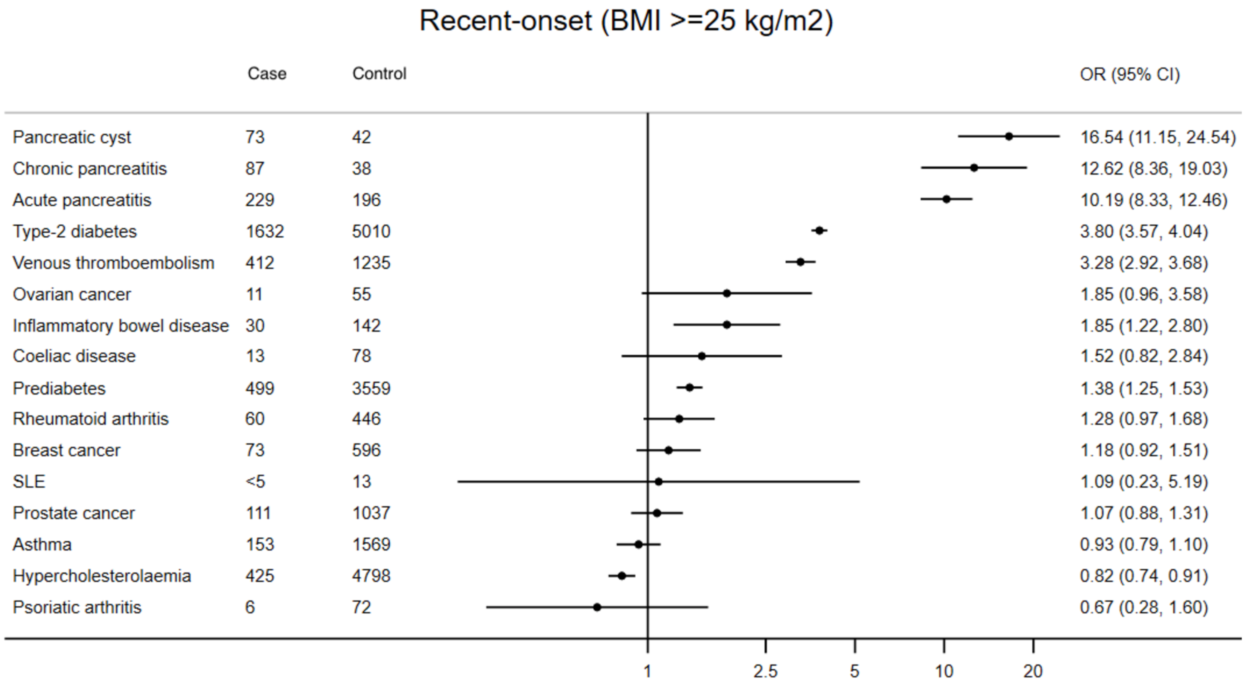

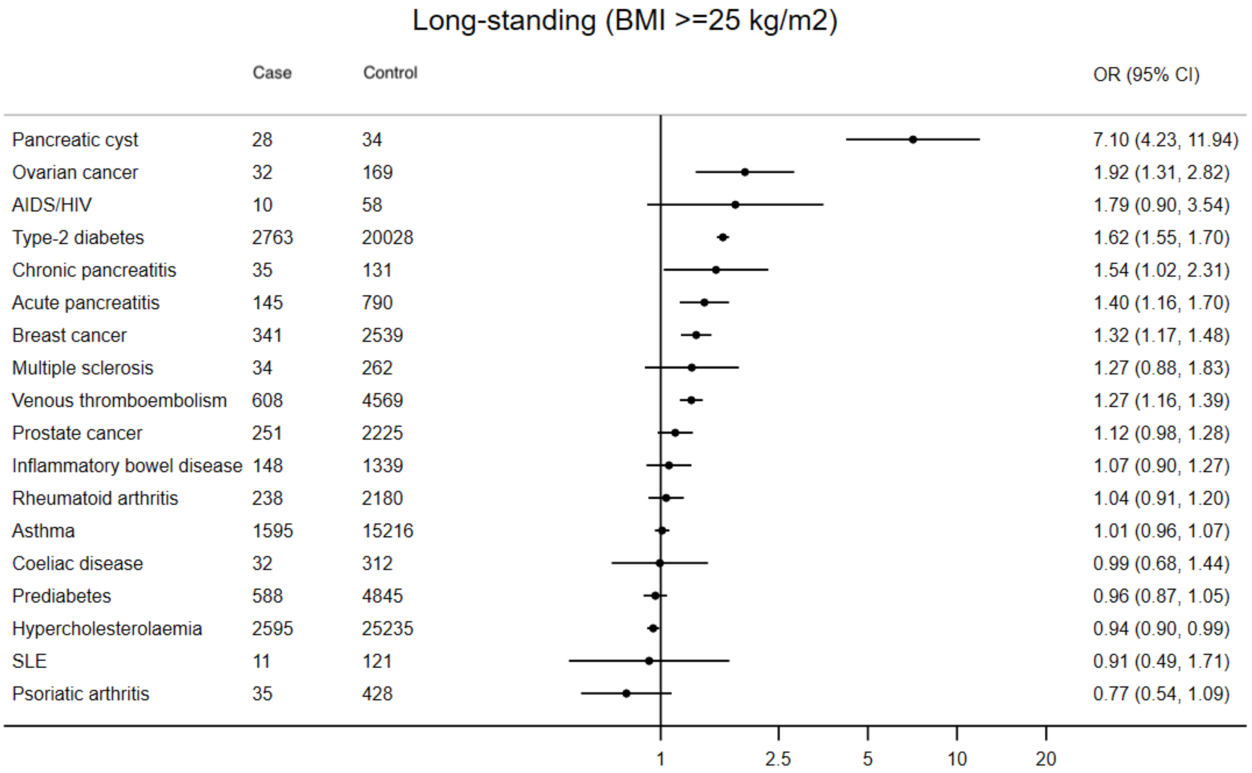

* Subgrouping variables were not matching factors in the case-control study design, hence, these subgroup analysis were performed without matching but instead analysed using mixed effects logistic regression using practice as a random-effect variable and additionally adjusted for other matching factors (age, sex, year of diagnosis).

Figure S15 (a): Subgroup analysis by smoking status - association of comorbidities and risk of PDAC in never smokers*

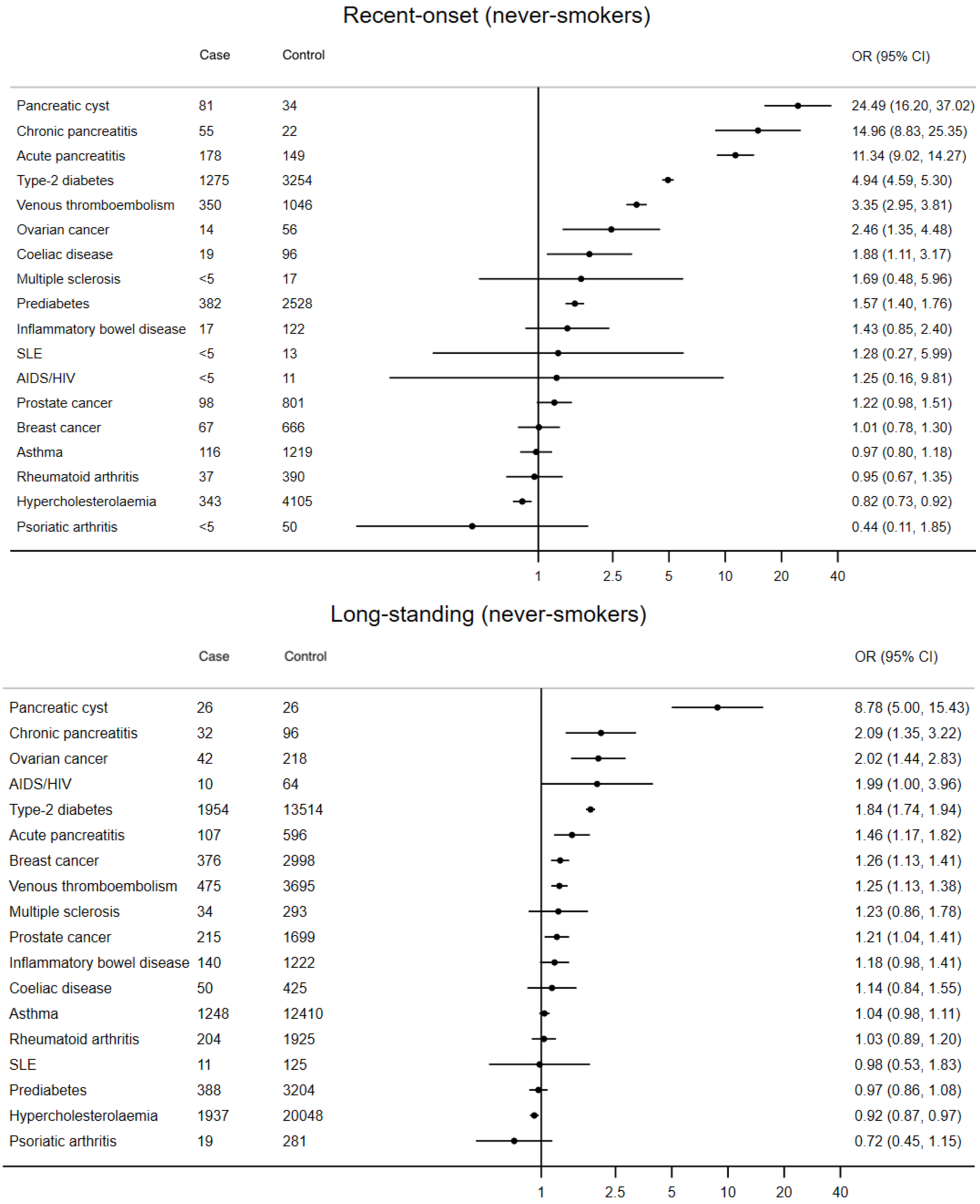

* Subgrouping variables were not matching factors in the case-control study design, hence, these subgroup analysis were performed without matching but instead analysed using mixed effects logistic regression using practice as a random-effect variable and additionally adjusted for other matching factors (age, sex, year of diagnosis).

Tan PS, *et al. Gut* 2023; 72:512–521. doi: 10.1136/gutjnl-2021-326522

Figure S15 (b): Subgroup analysis by smoking status - association of comorbidities and risk of PDAC in ever smokers (ex and current smokers)*

Recent-onset (ever-smokers)

	Case	Control	OR (95% CI)
Pancreatic cyst	66	30	13.46 (8.48, 21.35)
Acute pancreatitis	261	169	10.62 (8.64, 13.05)
Chronic pancreatitis	147	59	10.05 (7.23, 13.97)
Type-2 diabetes	1408	3108	4.70 (4.39, 5.04)
Venous thromboembolism	324	917	2.98 (2.61, 3.40)
Coeliac disease	20	64	2.37 (1.41, 3.97)
Ovarian cancer	7	29	1.91 (0.82, 4.42)
Prediabetes	423	2296	1.56 (1.40, 1.75)
Inflammatory bowel disease	29	135	1.42 (0.93, 2.17)
Prostate cancer	132	826	1.38 (1.14, 1.67)
Breast cancer	56	384	1.21 (0.91, 1.62)
Rheumatoid arthritis	47	372	0.99 (0.73, 1.36)
Asthma	156	1271	0.99 (0.83, 1.18)
Psoriatic arthritis	6	50	0.86 (0.35, 2.10)
AIDS/HIV	<5	8	0.83 (0.10, 6.92)
Hypercholesterolaemia	351	3404	0.79 (0.70, 0.89)
Multiple sclerosis	<5	16	0.52 (0.07, 4.03)

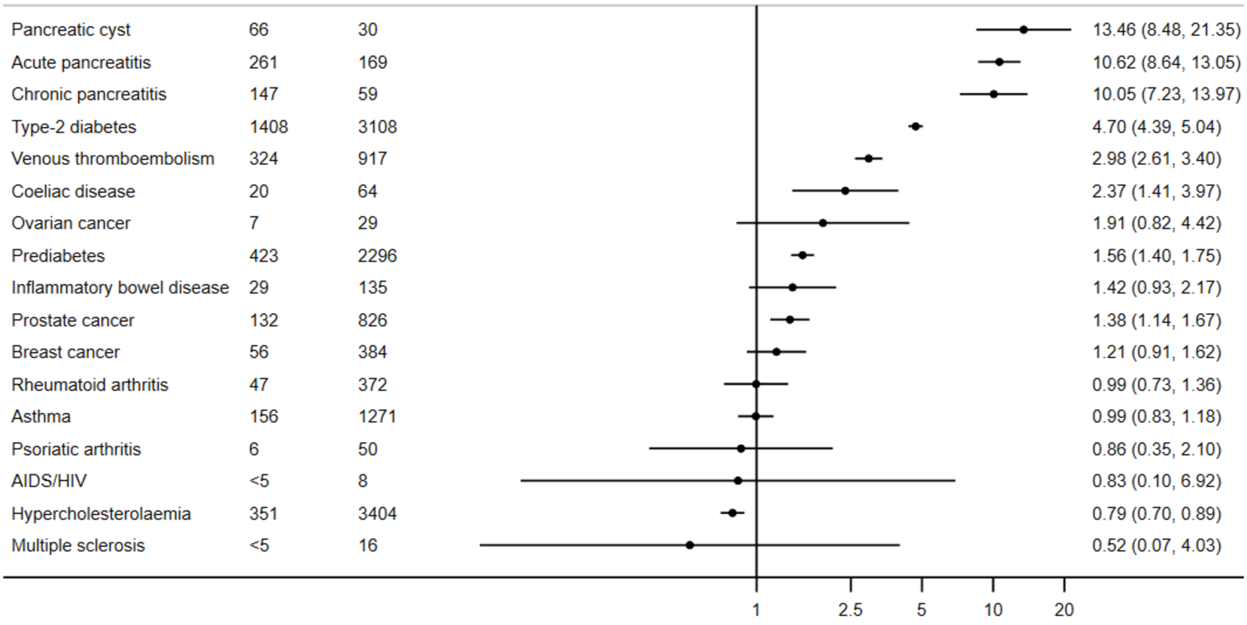

Long-standing (ever-smokers)

	Case	Control	OR (95% CI)
Pancreatic cyst	16	24	4.28 (2.20, 8.34)
AIDS/HIV	12	39	2.07 (1.07, 4.01)
Chronic pancreatitis	79	173	1.87 (1.39, 2.51)
Type-2 diabetes	2200	12698	1.86 (1.76, 1.96)
Acute pancreatitis	180	609	1.70 (1.41, 2.05)
Coeliac disease	50	257	1.50 (1.10, 2.05)
Ovarian cancer	16	92	1.44 (0.83, 2.48)
Breast cancer	255	1696	1.23 (1.07, 1.41)
Venous thromboembolism	457	3129	1.21 (1.09, 1.34)
Prostate cancer	220	1808	1.05 (0.91, 1.22)
Prediabetes	476	3114	1.05 (0.94, 1.16)
SLE	11	87	1.04 (0.55, 1.96)
Psoriatic arthritis	44	331	1.04 (0.75, 1.43)
Rheumatoid arthritis	238	1841	1.02 (0.89, 1.18)
Hypercholesterolaemia	2247	17272	1.02 (0.97, 1.07)
Asthma	1423	11218	1.01 (0.95, 1.07)
Multiple sclerosis	31	271	0.93 (0.64, 1.35)
Inflammatory bowel disease	139	1150	0.91 (0.76, 1.10)

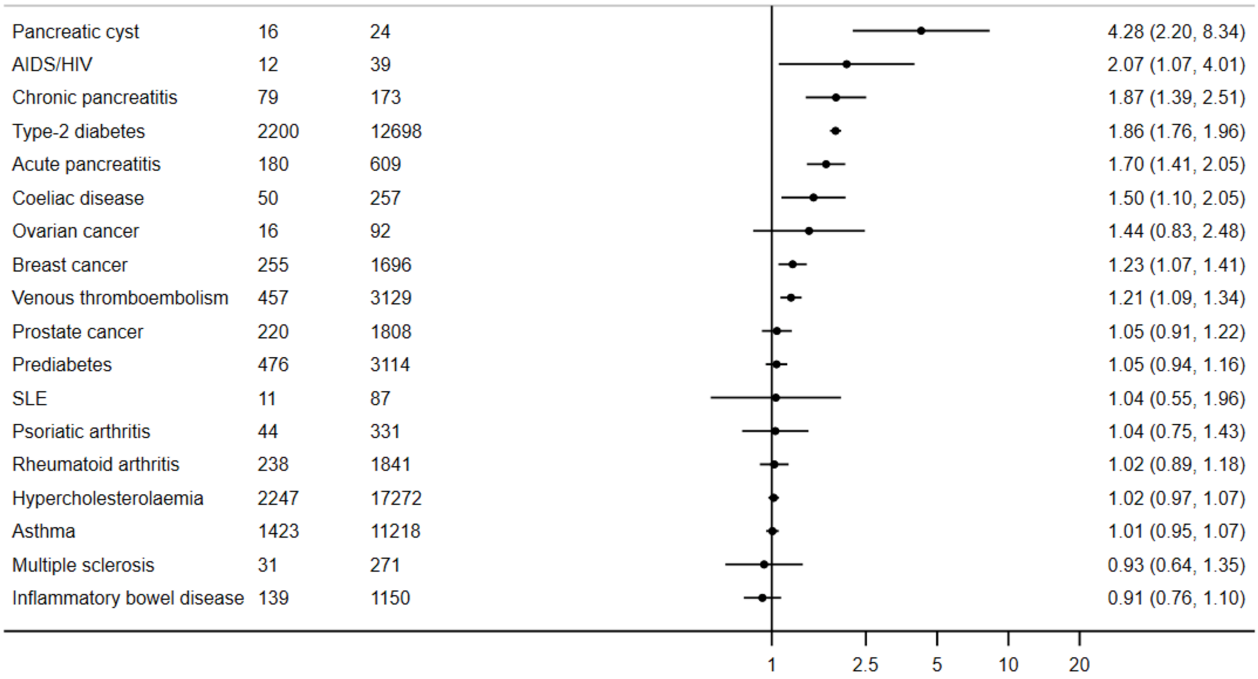

* Subgrouping variables were not matching factors in the case-control study design, hence, these subgroup analysis were performed without matching but instead analysed using mixed effects logistic regression using practice as a random-effect variable and additionally adjusted for other matching factors (age, sex, year of diagnosis).

Tan PS, *et al. Gut* 2023; 72:512–521. doi: 10.1136/gutjnl-2021-326522

Figure S16 (a): Subgroup analysis by type-2 diabetes status - association of comorbidities and risk of PDAC in individuals with type-2 diabetes*

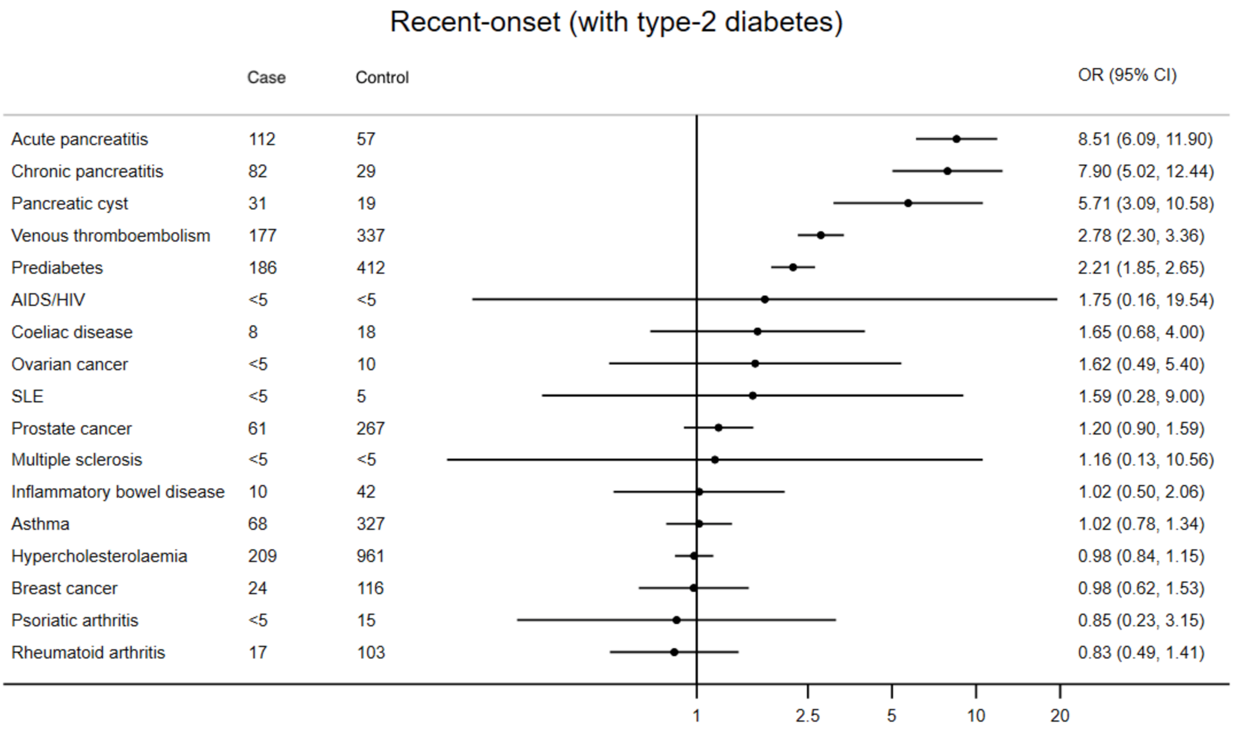

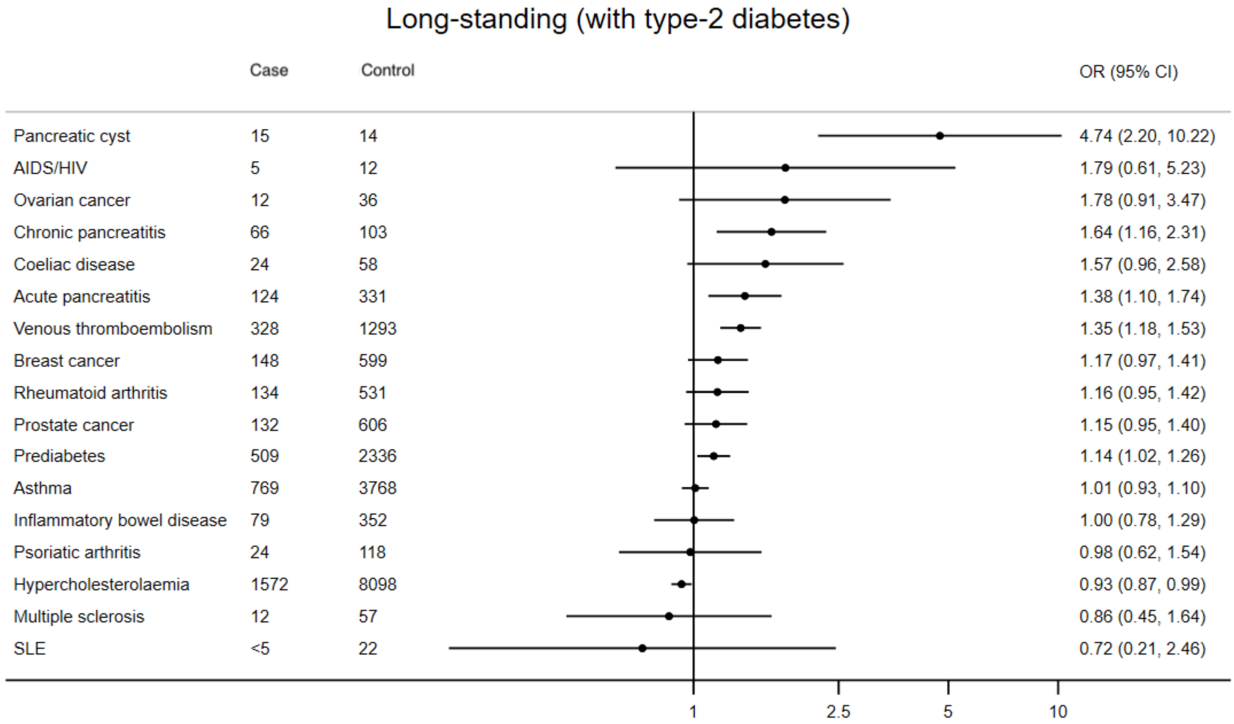

* Subgrouping variables were not matching factors in the case-control study design, hence, these subgroup analysis were performed without matching but instead analysed using mixed effects logistic regression using practice as a random-effect variable and additionally adjusted for other matching factors (age, sex, year of diagnosis).

Figure S16 (b): Subgroup analysis by type-2 diabetes status - association of comorbidities and risk of PDAC in individuals with no type-2 diabetes*

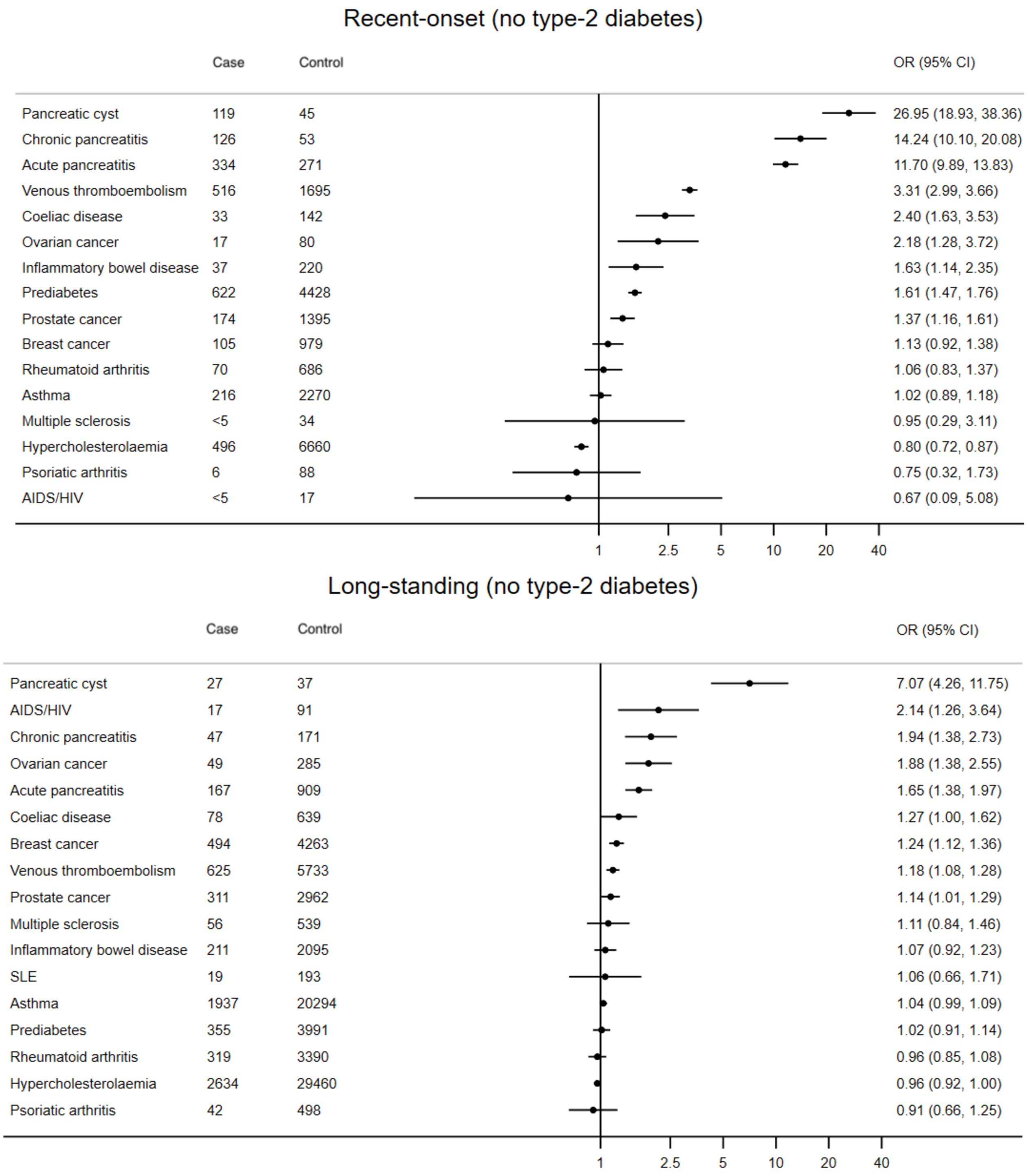

* Subgrouping variables were not matching factors in the case-control study design, hence, these subgroup analysis were performed without matching but instead analysed using mixed effects logistic regression using practice as a random-effect variable and additionally adjusted for other matching factors (age, sex, year of diagnosis).

Figure S17 (a): Subgroup analysis by body mass index (BMI) - association of medications and risk of PDAC in individuals with BMI <25kg/m^{2*}

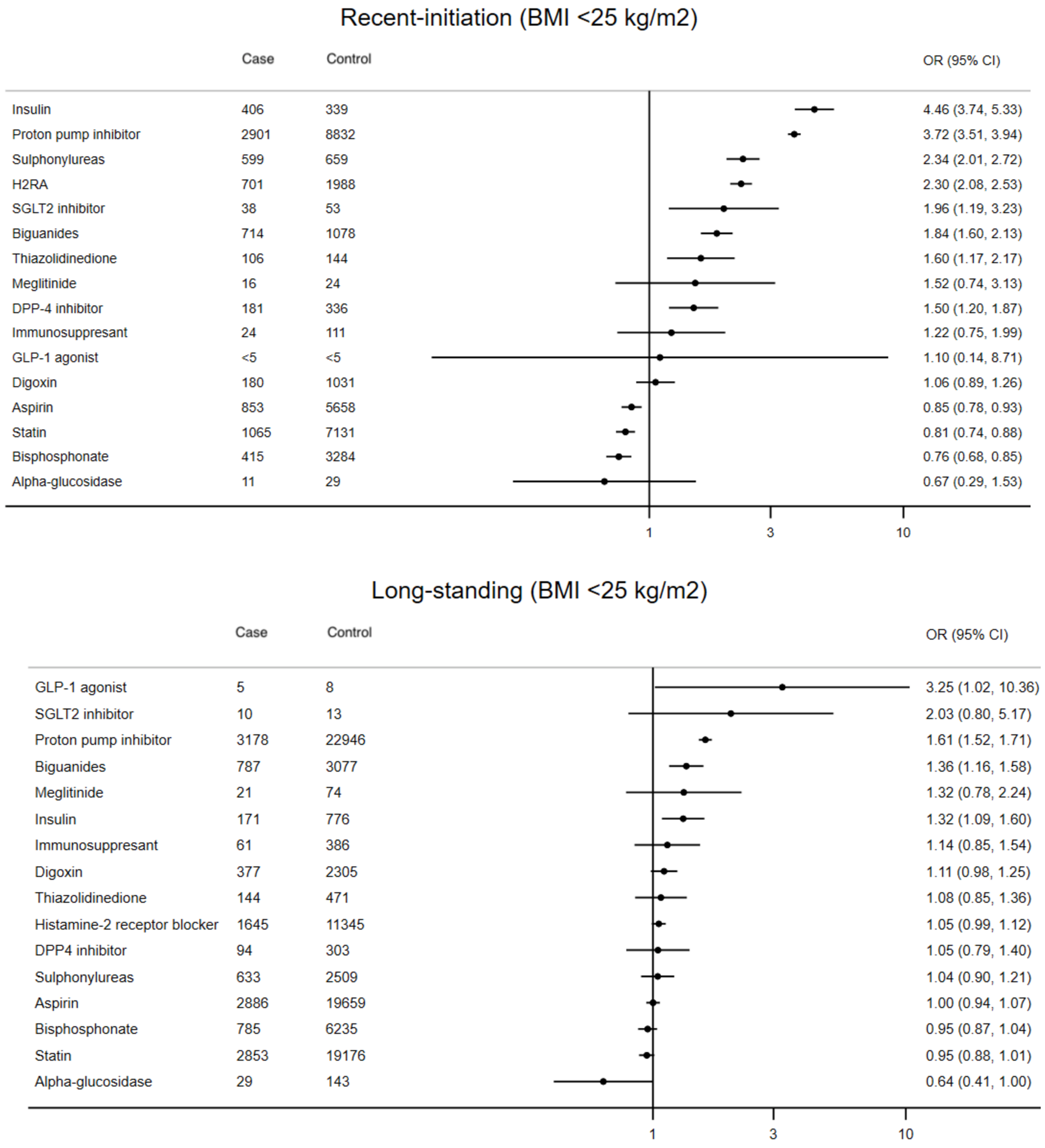

* Subgrouping variables were not matching factors in the case-control study design, hence, these subgroup analysis were performed without matching but instead analysed using mixed effects logistic regression using practice as a random-effect variable and additionally adjusted for other matching factors (age, sex, year of diagnosis).

Figure S17 (b): Subgroup analysis by body mass index (BMI) - association of medications and risk of PDAC in individuals with BMI >=25kg/m^{2*}

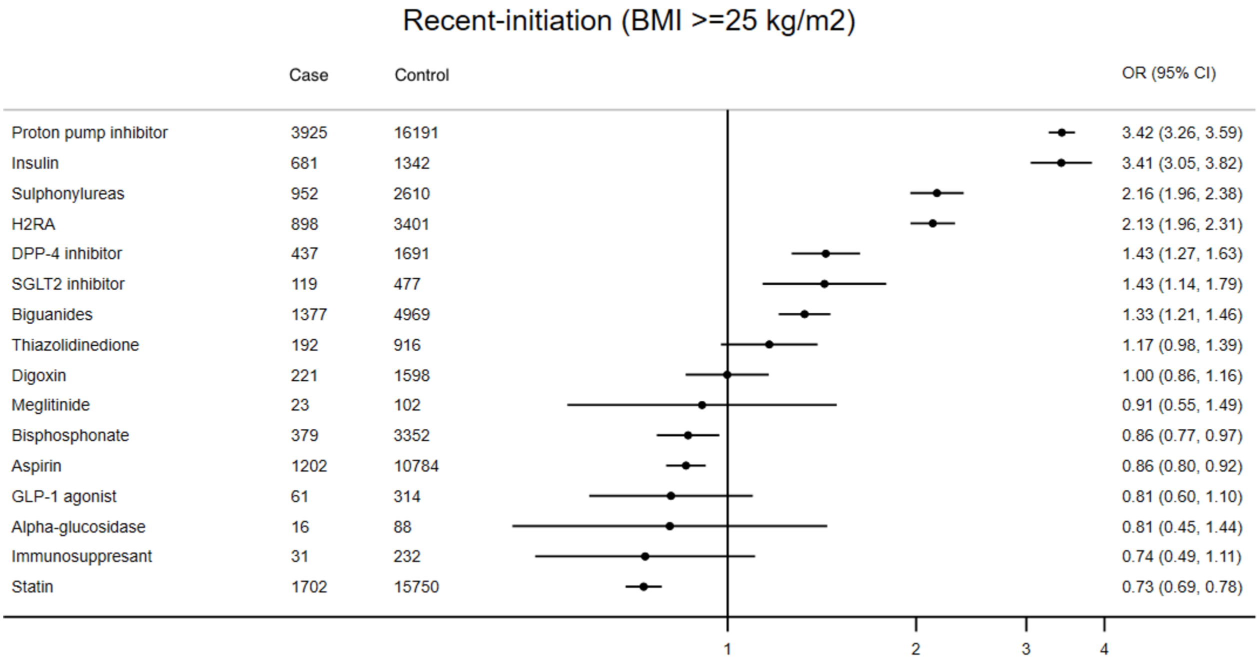

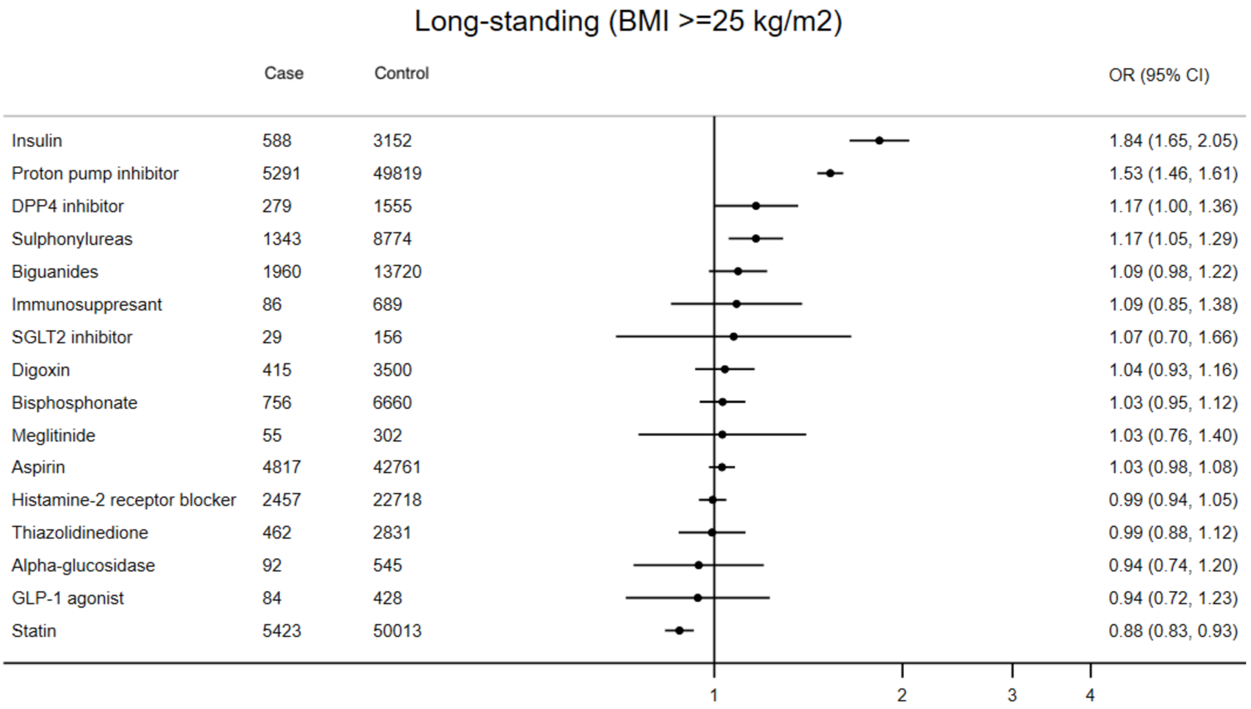

* Subgrouping variables were not matching factors in the case-control study design, hence, these subgroup analysis were performed without matching but instead analysed using mixed effects logistic regression using practice as a random-effect variable and additionally adjusted for other matching factors (age, sex, year of diagnosis).

Tan PS, *et al. Gut* 2023; 72:512–521. doi: 10.1136/gutjnl-2021-326522

Figure S18 (a): Subgroup analysis by smoking status - association of medications and risk of PDAC in never smokers*

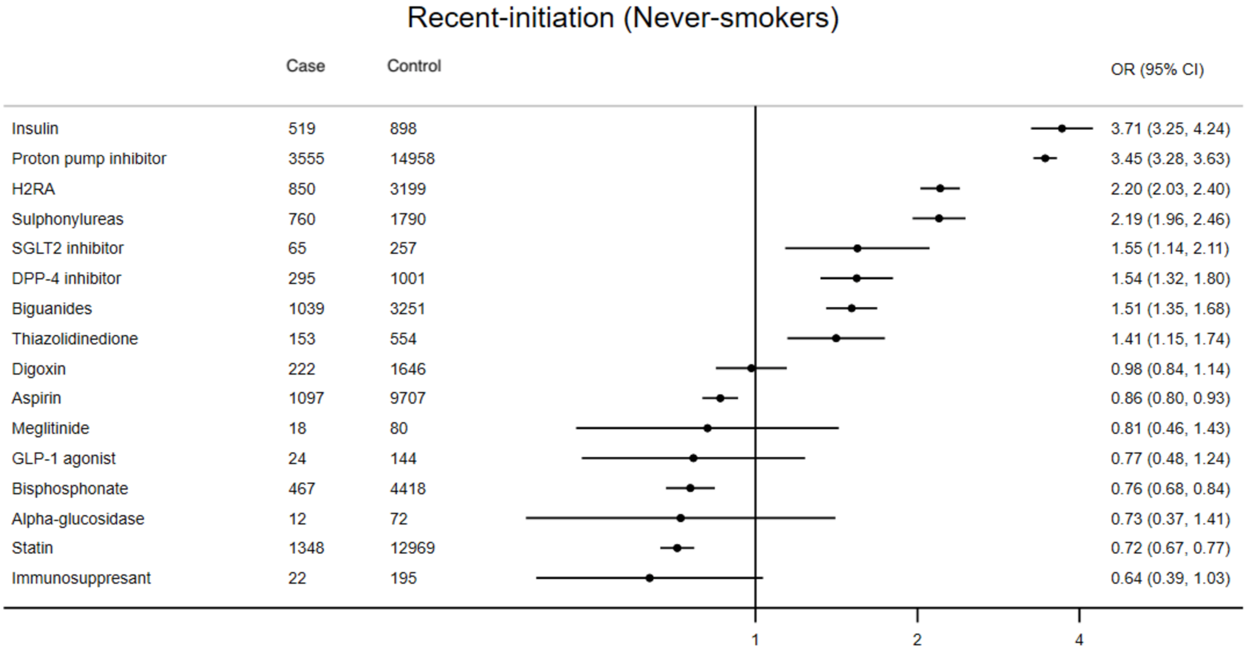

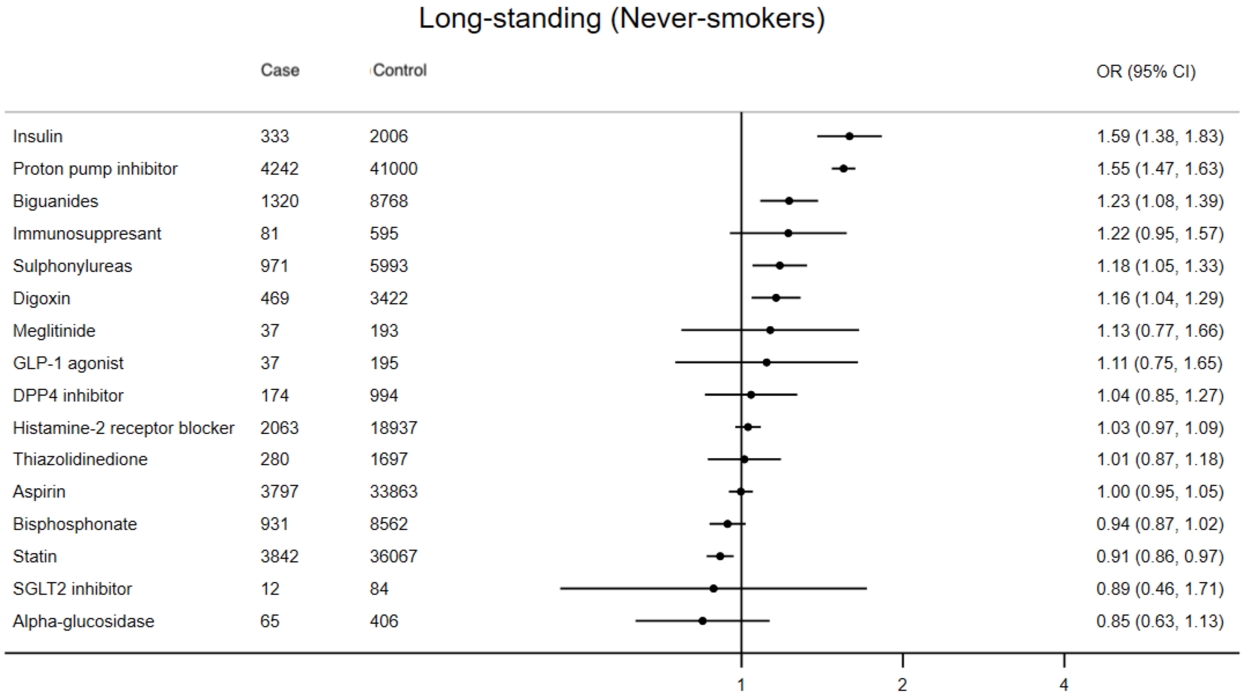

* Subgrouping variables were not matching factors in the case-control study design, hence, these subgroup analysis were performed without matching but instead analysed using mixed effects logistic regression using practice as a random-effect variable and additionally adjusted for other matching factors (age, sex, year of diagnosis).

Figure S18 (b): Subgroup analysis by smoking status - association of medications and risk of PDAC in ever smokers (ex and current smokers)*

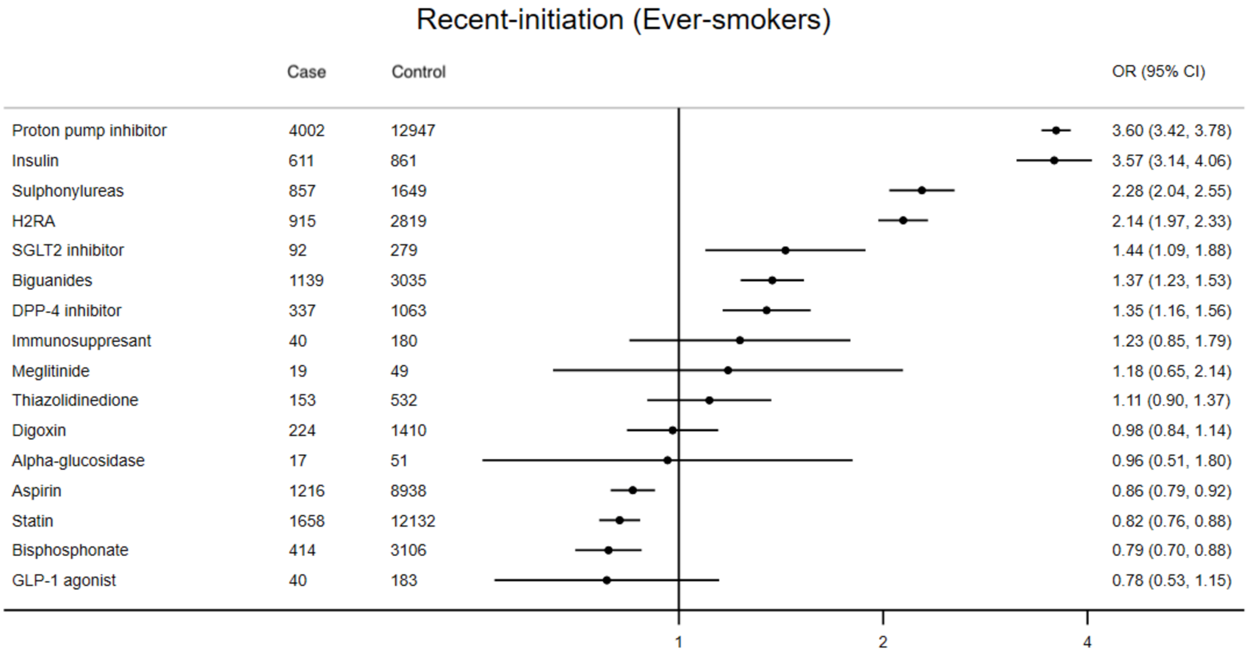

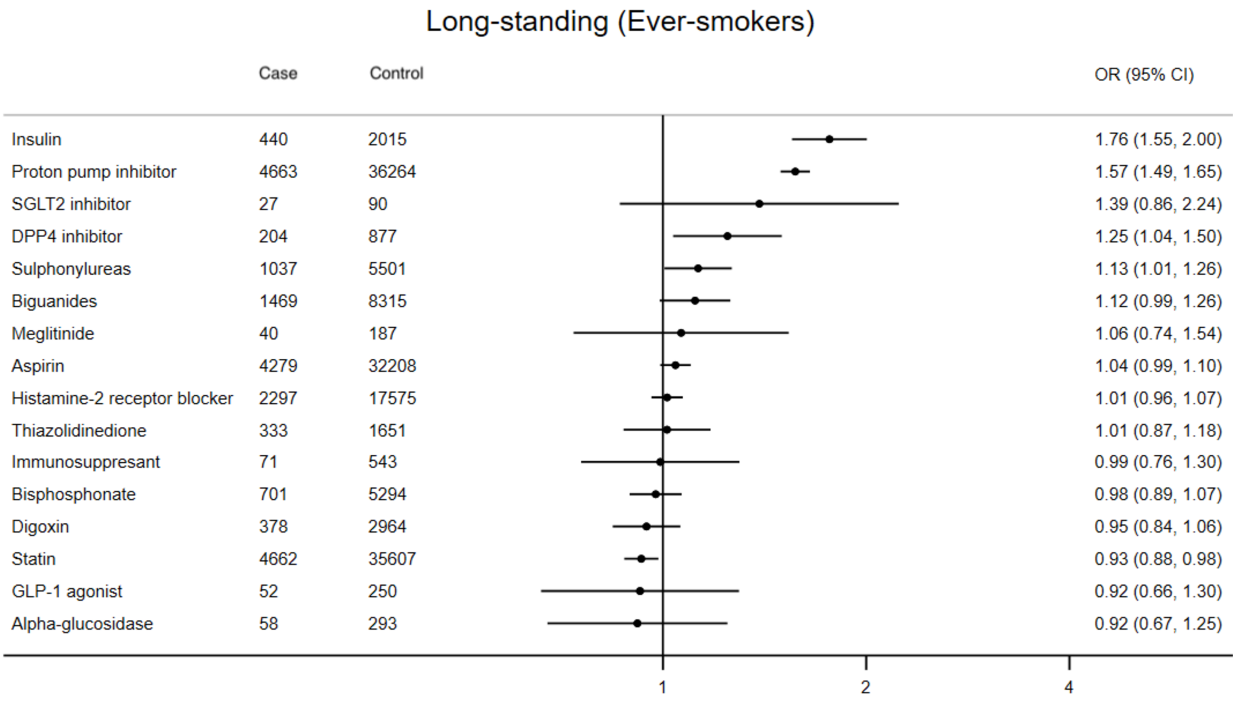

* Subgrouping variables were not matching factors in the case-control study design, hence, these subgroup analysis were performed without matching but instead analysed using mixed effects logistic regression using practice as a random-effect variable and additionally adjusted for other matching factors (age, sex, year of diagnosis).

Tan PS, *et al. Gut* 2023; 72:512–521. doi: 10.1136/gutjnl-2021-326522

Figure S19 (a): Subgroup analysis by type-2 diabetes status - association of medications and risk of PDAC in individuals with type-2 diabetes*

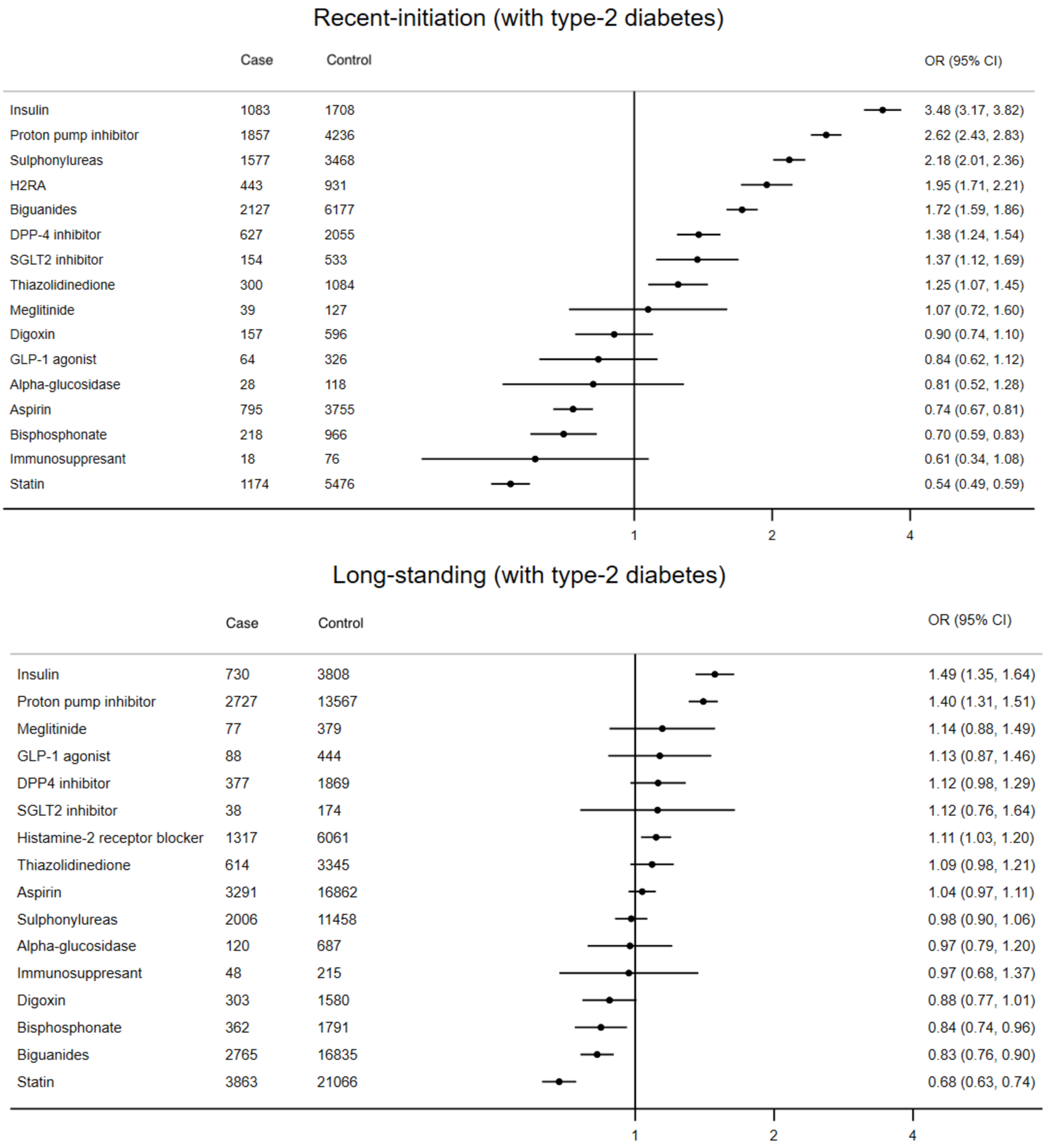

* Subgrouping variables were not matching factors in the case-control study design, hence, these subgroup analysis were performed without matching but instead analysed using mixed effects logistic regression using practice as a random-effect variable and additionally adjusted for other matching factors (age, sex, year of diagnosis).

Figure S19 (b): Subgroup analysis by type-2 diabetes status - association of medications and risk of PDAC in individuals with no type-2 diabetes*

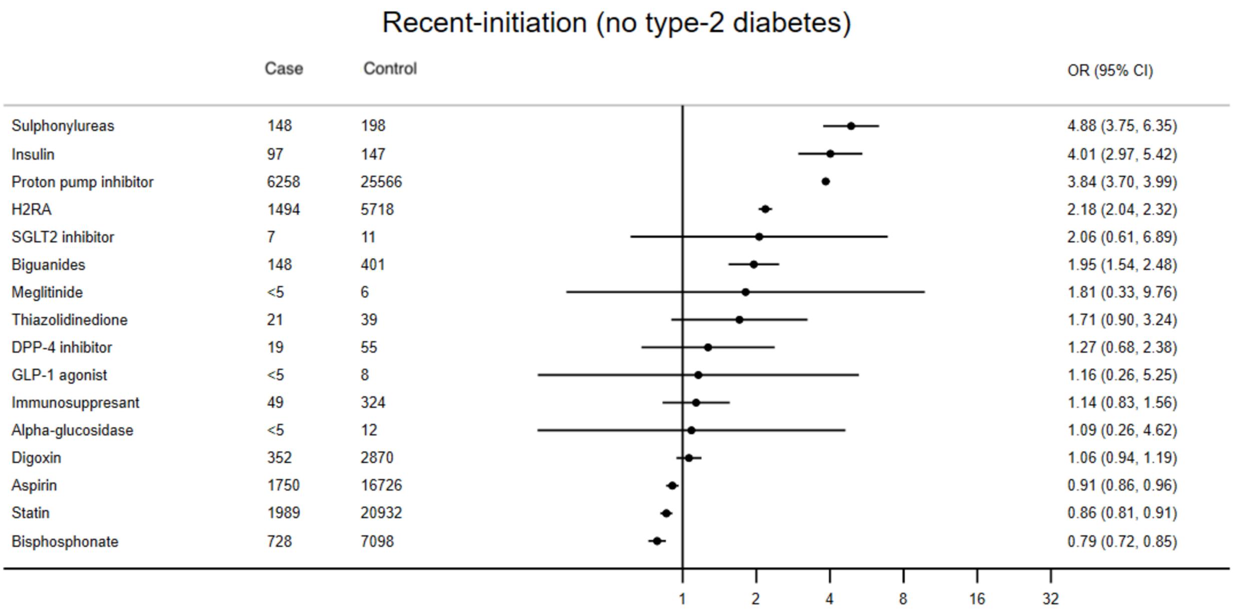

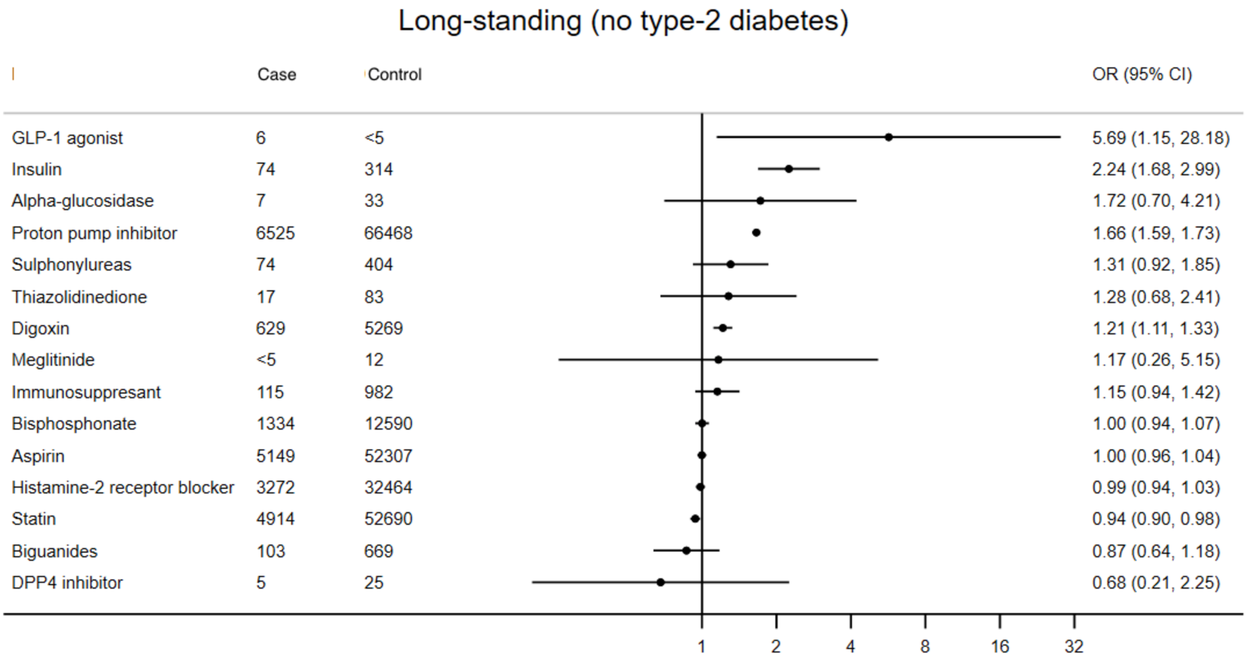

* Subgrouping variables were not matching factors in the case-control study design, hence, these subgroup analysis were performed without matching but instead analysed using mixed effects logistic regression using practice as a random-effect variable and additionally adjusted for other matching factors (age, sex, year of diagnosis).

Tan PS, *et al. Gut* 2023; 72:512–521. doi: 10.1136/gutjnl-2021-326522
